# Synthesis and Physicochemical Characterization of the Process-Related Impurities of Olmesartan Medoxomil. Do 5-(Biphenyl-2-yl)-1-triphenylmethyltetrazole Intermediates in Sartan Syntheses Exist?

**DOI:** 10.3390/molecules201219762

**Published:** 2015-12-01

**Authors:** Iwona Dams, Anna Ostaszewska, Maria Puchalska, Justyna Chmiel, Piotr Cmoch, Iwona Bujak, Agata Białońska, Wojciech J. Szczepek

**Affiliations:** 1 Pharmaceutical Research Institute, Rydygiera 8, Warsaw 01-793, Poland; a.ostaszewska@ifarm.eu (A.O.); m.puchalska@ifarm.eu (M.P.); j.chmiel@ifarm.eu (J.C.); piotr.cmoch@icho.edu.pl (P.C.); i.bujak@ifarm.eu (I.B.); w.szczepek@ifarm.eu (W.J.S.); 2 Institute of Organic Chemistry, Polish Academy of Sciences, Kasprzaka 44/52, 01-224 Warsaw, Poland; 3 Faculty of Chemistry, University of Wrocław, Joliot-Curie 14, 50-383 Wrocław, Poland; agata.bialonska@chem.uni.wroc.pl

**Keywords:** crystal structure, impurities, medoxomil, NMR spectroscopy, olmesartan, prodrugs, regioisomers, sartans, structure, synthesis

## Abstract

During the process development for multigram-scale synthesis of olmesartan medoxomil (OM), two principal regioisomeric process-related impurities were observed along with the final active pharmaceutical ingredient (API). The impurities were identified as *N*-1- and *N*-2-(5-methyl-2-oxo-1,3-dioxolen-4-yl)methyl derivatives of OM. Both compounds, of which *N*-2 isomer of olmesartan dimedoxomil is a novel impurity of OM, were synthesized and fully characterized by differential scanning calorimetry (DSC), infrared spectroscopy (IR), nuclear magnetic resonance spectroscopy (NMR) and high-resolution mass spectrometry/electrospray ionization (HRMS/ESI). Their ^1^H, ^13^C and ^15^N nuclear magnetic resonance signals were fully assigned. The molecular structures of *N*-triphenylmethylolmesartan ethyl (*N*-tritylolmesartan ethyl) and *N*-tritylolmesartan medoxomil, the key intermediates in OM synthesis, were solved and refined using single-crystal X-ray diffraction (SCXRD). The SCXRD study revealed that *N*-tritylated intermediates of OM exist exclusively as one of the two possible regioisomers. In molecular structures of these regioisomers, the trityl substituent is attached to the *N*-2 nitrogen atom of the tetrazole ring, and not to the *N*-1 nitrogen, as has been widely reported up to the present. This finding indicates that the reported structural formula of *N*-tritylolmesartan ethyl and *N*-tritylolmesartan medoxomil, as well as their systematic chemical names, must be revised. The careful analysis of literature spectroscopic data for other sartan intermediates and their analogs with 5-(biphenyl-2-yl)tetrazole moiety showed that they also exist exclusively as *N*-2-trityl regioisomers.

## 1. Introduction

**Scheme 1:**
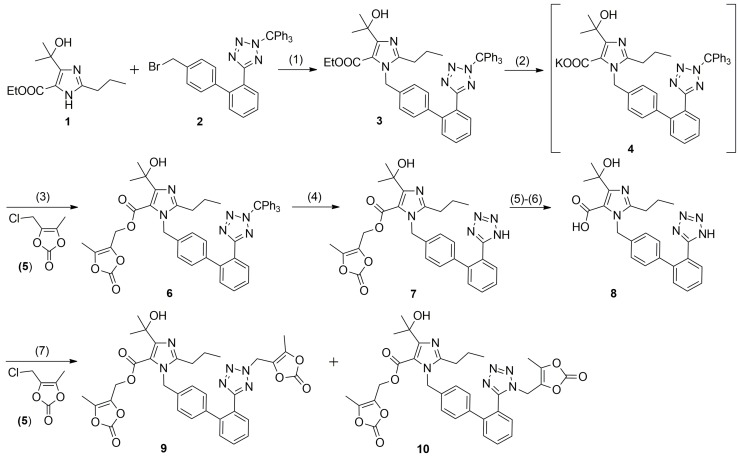
Synthesis of OM (**7**) and the impurities **9** and **10**. *Conditions:* (1) K_2_CO_3_, KI, DMF, 24 h at r.t., 83%; (2) KOH, DMF, 54–56 °C for 22 h; (3) K_2_CO_3_, KI, DMF, 22 h at r.t., 96%; (4) H_2_SO_4_-H_2_O, Me_2_CO, 50–55 °C for 2 h; 80%; (5) NaOH, MeOH, 24 h at r.t.; (6) AcOH, H_2_O; 96%; (7) K_2_CO_3_, KI, DMF, 22 h at r.t.

**Figure 1:**
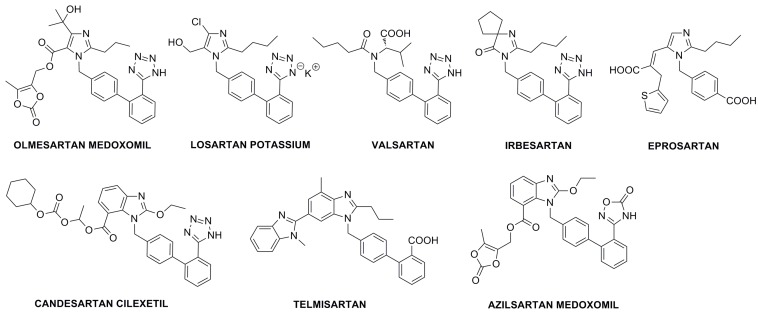
Structures of angiotensin II receptor blockers used as APIs in the treatment of hypertension.

Elevated blood pressure is one of the most important causes of death and disability worldwide, accounting for 7.6 million premature deaths and 92 million disability-adjusted life years annually [1]. Controlling blood pressure and prevention of its complications such as coronary heart disease, stroke, renal failure and eye damage are the main objectives for the treatment of hypertension [2,3]. In April 2002, the U.S. Food and Drug Administration (FDA) approved OM (**7**, Scheme 1) for the treatment of hypertension. The seventh in a growing class of antihypertensive agents known as the angiotensin II receptor blockers (Figure 1), the drug works by inhibiting effects of angiotensin II, a potent vasoconstrictor and one of the key contributors to cardiovascular and renal disease [4,5,6,7,8]. OM is a prodrug containing an ester moiety, which is rapidly and completely cleaved to release the active metabolite olmesartan (**8**) during absorption from the gastrointestinal tract. Potential advantages of this drug include once-daily dosing, an absence of significant adverse reactions, a well-tolerated side effect profile, and a cost-effective average wholesale price.

OM contains a biphenyl moiety substituted with an imidazol-1-ylmethyl and a tetrazol-5-yl group at C-4 and C-2′, respectively. Aside from OM, four other sartans used in clinical practice, *i.e.*, losartan, valsartan, irbesartan and candesartan, are 5-(biphenyl-2-yl)tetrazole derivatives. [2′-(*N*-Triphenylmethyltetrazol-5-yl)biphenyl-4-yl]methyl bromide [9] (**2**) is a common intermediate used in the synthesis of these sartan drugs. Despite its wide synthetic application, the available literature data and chemical databases are not in agreement on its chemical structure, giving formulas of the two bromides that differ substitution with trityl group at tetrazole *N*-1 (CAS 124750-51-2) and *N*-2 (CAS 133051-88-4). The similar structural dualism is observed for the *N*-tritylated intermediates **3** (CAS 144690-33-5 for *N*-1 tritylated and CAS 172875-59-1 for *N*-2 tritylated) and **6** (CAS 144690-92-6 for *N*-1 tritylated and CAS 1020157-01-0 for *N*-2 tritylated) in the synthesis of OM (**7**). As intermediates in active pharmaceutical ingredient (API) synthesis often afford numerous impurities affecting the quality of the final drug product, their structure explanation is essential for impurities identification and characterization. A complete physicochemical characterization, not only for an API, but also of its key synthetic intermediates, has recently become a requirement of both the U.S. FDA and the European Medicine Agency (EMA). Therefore, the real structure of the starting bromide **2** and the intermediate esters **3** and **6** should be unambiguously clarified.

The protection and deprotection of the tetrazole *N*-atom are essential steps during the synthesis of the sartan drugs containing 5-(biphenyl-2-yl)tetrazole moiety, and are often accompanied by side reactions and process-related impurities formation, influencing the high quality of the final active pharmaceutical ingredient (API). The available literature data indicate that tetrazolic acids of sartans, formed as a result of undesired tetrazole *N*-atom deprotection from trityl group, may exist as mixtures of 1*H*- and 2*H*- tautomeric forms that subsequently undergo adverse alkylation reactions to afford mixtures of *N*-1 and *N*-2-alkyl regioisomeric impurities. Alkylation of some sartan tetrazolic acids with primary alkyl halides, *i.e.*, methyl iodide, iodomethyl pivalate, medoxomil chloride, bromide **2**, or ethyl iodide, under different reaction conditions afforded mixtures of regioisomeric *N*-1- and *N*-2-alkyl derivatives [10,11,12]. It should be noted that Rádl *et al.* wrongly assigned the position of trityl group in their [2′-(1-trityl-1*H*-tetrazol-5-yl)biphenyl-4-yl]methyl substituent [11].

Recently, we have developed an improved, scalable, cost-effective and environment-friendly technology for the industrial-scale synthesis of OM (**7**, Scheme 1) based on the general route described by Yanagisawa *et al.* [13,14] During the process development, the two regioisomeric impurities **9** and **10** of OM were observed along with the final API samples on the levels 0.03%–0.18% and 0.02%–0.13%, respectively. The impurities were identified as *N*-1- and *N*-2-(5-methyl-2-oxo-1,3-dioxolen-4-yl)methyl derivatives of OM. To the best of our knowledge, the isomeric *N*-2-medoxomil impurity **9** is a new compound that has never been identified. The determination of a drug substance impurity profile, including potential degradation products and process-related impurities, is critical for the safety assessment of API and manufacturing process thereof. According to the guidelines issued by the International Conference on Harmonization (ICH) and European Pharmacopoeia it is mandatory to identify and characterize the impurities in a pharmaceutical product if present above the accepted limits of 0.1% [15,16]. Therefore, there was a need for a complete physicochemical characterization not only for OM API but also its regioisomeric impurities **9** and **10** and the key synthetic intermediates **3** and **6** to comply with current FDA and EMA requirements.

Herein, we discussed the origin of formation, synthesis, identification and characterization of both *N*-1- and *N*-2-(5-methyl-2-oxo-1,3-dioxolen-4-yl)methyl regioisomeric impurities **9** and **10** of OM (**7**), respectively. On the basis of analytical data (IR, NMR and SCXRD), we disproved the structural dualism of the tetrazole tritylated compounds **2**, **3** and **6**, existing in the literature data and chemical databases, and determined their real structure as *N*-2-tritylated tetrazole regioisomers. Additionally, the careful analysis of literature spectroscopic data for other tritylated sartan intermediates with 5-(biphenyl-2-yl)tetrazole moiety showed that they also exist exclusively as *N*-2-trityl derivatives.

## 2. Results and Discussion

### 2.1. Synthesis of Olmesartan Medoxomil *(**7**)* and N-1- and N-2-Medoxomil Impurities ***9*** and ***10***

The medoxomil ester **6** (Scheme 1) is synthesized in a one-pot process comprising hydrolysis of ethyl ester **3** with KOH and alkylation with medoxomil chloride (**5**) in *N*,*N*-dimethylformamide (DMF) or dimethyl sulfoxide (DMSO). Deprotection of tetrazole nitrogen from trityl group during basic hydrolysis of ethyl ester **3** to potassium salt **4** is very important, as dipotassium salt of olmesartan acid (**8**) resulted is immediately involved in the next alkylation step of a one-pot process giving a mixture of *N*-1- and *N*-2-medoxomil regioisomers **9** and **10**. The effective synthesis of intermediate **6** from the *in situ* prepared potassium salt **4** always requires the excess of medoxomil chloride **5**. The use of an equimolar amount of **5** does not prevent impurities formation, as the alkylation of tetrazole unit is faster than alkylation of carboxyl group [10].

The content of dipotassium salt of olmesartan acid (**8**) in the purified ester **6** samples, detected as acid **8**, ranged from 0.13% to 1.28% (RRT 0.47) as detected by HPLC (Table 1). However, in the samples of the ester **6** two other impurities on the levels 0.16%–0.67% (RRT 0.61, **9**) and 0.13%–1.06% (RRT 0.53, **10**) were also observed. The same compounds, on the comparable levels 0.15%–0.64% (RRT 2.22, **9**) and 0.12%–0.97% (RRT 1.86, **10**), were detected in the crude OM (**7**) samples as potential impurities of the final pharmaceutical substance (Table 2). The purification attempts indicated that removal of both impurities from the final product, below the level of 0.1% accepted by ICH guidelines, to achieve pharmaceutical grade purity OM could be problematic. Since the European and the United States Pharmacopoeias on OM also specify none of the detected impurities [17,18], both compounds **9** and **10** had to be identified, synthesized and characterized thoroughly.

**Table 1:** Synthesis and HPLC data of *N*-tritylolmesartan medoxomil (**6**) samples.

Example	Solvent	Purification Extraction/Crystallization	HPLC (RRT) (%)						Yield (%)
			0.47	0.53	0.61	0.88	0.98	1.00	
			8	10	9	4	3	6	
**1**	DMF	CH_2_Cl_2_/*i*-PrOH–H_2_O	0.13	**0.56**	**0.56**	‒	0.08	97.11	96
**2**	DMF	AcOEt/AcOEt–hexanes	0.16	**1.06**	**0.67**	0.06	0.01	97.08	68
**3**	DMSO	AcOEt/EtOH–H_2_O	1.28	**0.24**	**0.31**	0.02	0.01	97.21	86
**4**	DMF	CH_2_Cl_2_/Me_2_CO–H_2_O	0.16	**0.15**	**0.18**	0.03	0.04	98.88	87
**5**	DMF	CH_2_Cl_2_/MeCN–H_2_O	0.14	**0.13**	**0.16**	0.04	0.02	97.75	92

**Table 2:** Synthesis and HPLC data of olmesartan medoxomil (OM, **7**) samples.

Example	Solvent Acid	Work up	Crystallization	HPLC purity (RRT) (%)			Yield (%)
				1.00	1.86	2.22	
				7	10	9	
**1**	Me_2_CO	H_2_O/filtration	Crude	98.63	**0.51**	**0.64**	‒
	H_2_SO_4_–H_2_O	CH_2_Cl_2_/Na_2_CO_3_[aq]	Me_2_CO–AcOEt	99.20	**0.08**	**0.04**	80
**2**	Me_2_CO	H_2_O/filtration	Crude	97.97	**0.97**	**0.61**	‒
	H_2_SO_4_–H_2_O	CH_2_Cl_2_/Na_2_CO_3_[aq]	Me_2_CO	99.22	**0.10**	**0.08**	65
**3**	Me_2_CO	H_2_O/filtration	Crude	97.88	**0.21**	**0.34**	‒
	H_2_SO_4_–H_2_O	CH_2_Cl_2_/Na_2_CO_3_[aq]	THF–Me_2_CO–H_2_O	98.24	**0.13**	**0.18**	67
**4**	Me_2_CO	H_2_O/filtration	Crude	99.15	**0.12**	**0.15**	‒
	H_2_SO_4_–H_2_O	CH_2_Cl_2_/NaHCO_3_[aq]	Me_2_CO–H_2_O	99.47	**0.02**	**0.03**	86
**5**	Me_2_CO	H_2_O/filtration	Crude	99.23	**0.13**	**0.16**	‒
	H_2_SO_4_–H_2_O	CH_2_Cl_2_/Na_2_CO_3_[aq]	CH_3_CN–H_2_O	99.46	**0.02**	**0.03**	90

During a one-pot process of the medoxomil ester **6** synthesis, the dipotassium salt of olmesartan acid (**8**) detected by thin layer chromatography (TLC) in the reaction mixture after basic hydrolysis of the ethyl ester **3** almost disappeared in the next alkylation step with medoxomil chloride (**5**). This fact, in accordance with the available literature data on isomeric impurities of sartans [10,11,12], threw our suspicion to the possibility of dimedoxomil impurities formation by *O*- and *N*-alkylations of olmesartan acid (**8**). The synthesis of regioisomeric dimedoxomil impurities **9** and **10** was achieved according to the route described in the Scheme 1. Thus, OM (**7**) was subjected to basic hydrolysis with sodium hydroxide (NaOH) in methanol at ambient reaction conditions to afford after acidification the olmesartan acid (**8**) in 96% yield. The structure of **8** was confirmed by spectral analysis. Alkylation of the acid **8** with the excess of medoxomil chloride (**5**) in DMF in the presence of K_2_CO_3_ provided a mixture of dimedoxomil compounds **9** and **10** in the ratio of 42.56%:57.44% by HPLC. The isomers were separated and purified by column chromatography on silica gel with 50%–100% AcOEt/hexanes gradient elution to afford *N*-2-medoxomil derivative **9** (R_f_ = 0.75 for 30% MeOH/AcOEt) and *N*-1-medoxomil derivative **10** (R_f_ = 0.64 for 30% MeOH/AcOEt). The structure of regioisomers was established on the basis of spectroscopic methods.

Formation of two regioisomeric dimedoxomil derivatives of olmesartan (**8**) is in good agreement with previous observations that alkylation of compounds possessing 5-(biphenyl-2-yl)tetrazole moiety with primary alkyl halides under different reaction conditions gives the mixtures of two regioisomeric *N*-1- and *N*-2-alkyl derivatives. The observed ratio of *N*-1- to *N*-2-alkyl regioisomer was 75.4:24.6 for methyl (-CH_3_) [10], 75:25 for pivaloyloxymethyl (-CH_2_-OOC-*tert*-Bu) [10], 60.2:39.8 for [2'-(2-trityl-2*H*-tetrazol-5-yl)biphenyl-4-yl]methyl [11], 60:40 for medoxomil [10] and 47.3:52.7 for ethyl (-CH_2_CH_3_) [12]. In contradiction with these data was the result of the alkylation of olmesartan (**8**) with medoxomil chloride (**5**) in acetone, in the presence of K_2_CO_3_ and tetra-*n*-butylammonium bromide, described by Venkanna *et al.* [19] The authors presented the false information that the reaction leads to a single crystalline product with the yield 93% and they attributed the structure of *N*-1-medoxomil derivative to the prepared compound without any evidence. In our hands, the reproduction of the reaction according to Venkanna procedure gave a mixture of dimedoxomil derivatives with the ratio of *N*-1- to *N*-2-regioisomer 43.5:56.5 and acetone condensation products. The attempts to isolate and purify the *N*-1- (**10**) regioisomer by the Authors’ procedure were unsuccessful, affording a dark-brown oily mixture of *N*-2- (**9**) and *N*-1- (**10**) medoxomil derivatives and acetone condensation products.

As the contents of dimedoxomil isomers **9** and **10** in the purified medoxomil ester **6** and the crude OM (**7**) samples were on the comparable levels, we checked the stability of both impurities under detritylation of OM conditions. Thus, the pure isomers **9** and **10** were heated with a three-fold excess of H_2_SO_4_ in an acetone-water solution. After being stirred for 2 h, the reaction mixture contained the unreacted isomer **9** or **10** as the main product, and the trace amounts of OM (**7**) and the olmesartan acid (**8**). The experiments confirmed that both impurities **9** and **10** are hardly decomposable under acidic detritylation conditions, thus contaminating the crude final product.

### 2.2. Determination of the Structure of the Bromide ***2*** and the Intermediates ***3*** and ***6***

Alkylation of 5-phenyltetrazole with trityl chloride under alkaline conditions as well as with triphenylmethanol under acidic conditions should lead exclusively to the 5-phenyl-2-trityltetrazole [20,21]. Therefore, [2′-(*N*-triphenylmethyltetrazol-5-yl)biphenyl-4-yl]methyl bromide (**2**), the commercially available in kg scale intermediate to the syntheses of sartans, should be tritylated at the 2-position of tetrazole. The crystal structure of [2′-(*N*-triphenylmethyltetrazol-5-yl)biphenyl-4-yl]methyl bromide obtained according to the procedure given by Aldrich was solved using a single crystal grown from butan-2-one [22]. Although the structural formula shown in the paper is incorrect (tetrazole connected phenyl substituted with *p*-bromophenyl at *metha* position), the presented molecular structure unequivocally proves the presence of the trityl substituent on the nitrogen *N*-2 atom of tetrazole. Zhao *et al.* using the single crystals grown from ethyl methyl ketone and ethyl acetate confirmed the same position of trityl substituent in two polymorphs of **2** later [23]. The comparison of the ^1^H- and ^13^C-NMR data recorded for our [2′-(2-triphenylmethyltetrazol-5-yl)biphenyl-4-yl]methyl bromide (**2**) and bromides **2** described in the literature in many cases shows differences (see Supplementary Materials; Tables S1–S3, respectively); however, no documented case of a compound with the trityl substituent at the *N*-1-position of 5-(biphenyl-2-yl)tetrazole was found. Thus, the starting bromide **2** for syntheses of sartans is [2′-(2-triphenylmethyl-2*H*-tetrazol-5-yl)biphenyl-4-yl]methyl bromide.

**Figure 2:**
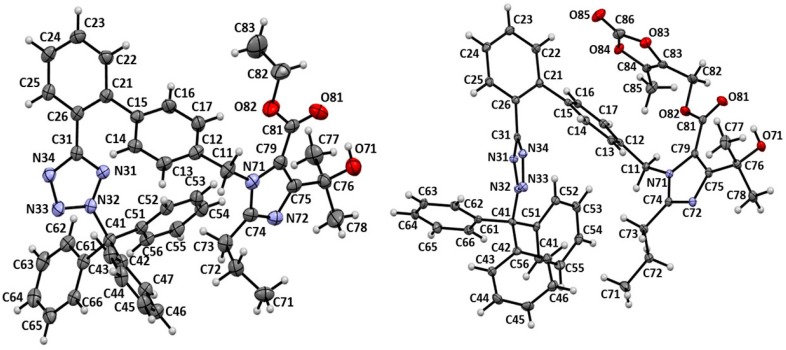
Molecular structure of *N*-tritylolmesartan ethyl (**3**, **left**) and *N*-tritylolmesartan medoxomil (**6**, **right**).

Treatment of **1** with the bromide **2** in the presence of K_2_CO_3_ and KI in DMF (Scheme 1) gave the ethyl ester **3**. The SCXRD measurement of this product showed that the molecule contains tetrazole ring tritylated at *N*-2 position (Figure 2, left). Thus, the intermediate compound **3** is ethyl 4-(1-hydroxy-1-methylethyl)-2-propyl-1-[2′-(2-triphenylmethyl-2*H*-tetrazol-5-yl)biphenyl-4-yl]methyl-1*H*-imidazole-5-carboxylate. The next intermediate in the synthesis of OM, *N*-tritylolmesartan medoxomil (**6**), is obtained in two steps from **3** (Scheme 1). Alkaline hydrolysis of **3** generates the potassium salt **4**. Treatment of the *in situ* formed salt **4** with **5** yields medoxomil ester **6**. As expected, its SCXRD also showed tritylation of the tetrazole at *N*-2 position and the correct product name is (5-methyl-2-oxo-1,3-dioxolen-4-yl)methyl 4-(1-hydroxy-1-methylethyl)-2-propyl-1-[2′-(2-triphenylmethyl-2*H*-tetrazol-5-yl)biphenyl-4-yl]-methyl-1*H*-imidazole-5-carboxylate (Figure 2, right). The results described above strongly prove retention of *N*-2 trityl position in the course of sartan synthesis. In fact, the trityl losartan obtained by condensing (2-butyl-4-chloro-1*H*-imidazol-5-yl)methanol with the bromide **2** in the presence of K_2_CO_3_ in DMF, reported in chemical databases as both *N*-1- (CAS 124751-00-4) and *N*-2-trityl (CAS 133909-99-6), is tritylated at *N*-2 of tetrazole, which was unambiguously proved by SCXRD [24].

The structures of the starting bromide **2** and the intermediates **3** and **6** in the synthesis of OM, as well as the impurities **9** and **10**, were confirmed on the basis of the detailed analysis of the ^1^H- and ^13^C-NMR results. A careful analysis of the ^1^H- and ^13^C-NMR chemical shifts and ^1^H/^13^C gradient selected HSQC and HMBC (g-HSQC and g-HMBC) correlations allowed to correctly assign ^1^H- and ^13^C-NMR signals. Identification of H-3′ protons of these compounds was done based on the analysis of the ^1^H-^13^C g-HMBC long-range ^1^H-^13^C correlations. It was essential from the viewpoint of tetrazole substitution. In the case of **2**, **3** and **6** H-3′ proton at *ca.* 7.80–8.00 ppm gave correlation with carbon at *ca.* 164 ppm, identifying tetrazole carbon signal as characteristic for 2,5-disubstituted tetrazoles. According to the literature data, their C-5 tetrazole (C-5T) carbon atom signal lies in the range of 159.0–167.8 ppm (except for 5-*tert*-butyl substituent) [10,25,26,27,28]. By contrast, C-5T carbon atom signal of 1,5-disubstituted tetrazoles (except for 5-*tert*-butyl, 5-(2-furyl) and 5-(2-thienyl) substituents) appears within the 150.2–156.6 ppm range [10,26,29,30]. The observed difference of the chemical shifts for the two isomeric compounds calculated as Δδ = δ_2,5-isomer_ − δ_1,5-isomer_ lies in the range of 10.6–12.0 ppm [26,28,29]. Moreover, it has been also shown that the ^13^C-NMR chemical shifts of tetrazole *N*-C_α_ (TN-C_α_) carbon atom of 2,5-disubstituted tetrazoles are higher compared to the corresponding resonances in the 1,5-disubstituted isomers [10,26,31,32]. Therefore, comparison of ^13^C-NMR data of **2**, **3**, **6**, **9** and **10** with those presented for 2,5- and 1,5-disubstituted tetrazoles allows unambiguously identifying and differentiating isomeric *N*-1- and *N*-2-substituted tetrazoles. The same rules were applied by Kubo *et al.* [10] in the structure assignment of the *N*-1- and *N*-2-alkyl isomeric products obtained by alkylation of candesartan derivative CV-11194 with methyl iodide, iodomethyl pivalate and medoxomil chloride.

### 2.3. Determination of the Structure of Dimedoxomil Impurities ***9*** and ***10***

The [M + H]^+^ values, *m*/*z* 671.2454 and 671.2463, obtained for both impurities **9** and **10** correspond to C_34_H_35_N_6_O_9_. The elemental composition responds to a dimedoxomil derivative of olmesartan (**8**). However, both isolated products **9** and **10** have different melting points, IR and ^1^H-NMR data than the unknown compound depicted by Venkanna *et al.* [19] as *N*-1-medoxomil impurity **10**. It may be assumed that the compound described by these Authors exists in a different crystalline form. Then the two samples of compound **10** may exhibit different melting points and IR data. However, the ^1^H-NMR spectra recorded in the same solvent should be the same or very similar. A careful comparison of the ^1^H-NMR data (300 MHz, CDCl_3_) recorded by the Authors for the supposed *N*-1-medoxomil compound with ours ^1^H-NMR data (200 MHz or 600 MHz in CDCl_3_) obtained for both dimedoxomil isomers **9** and **10** shows essential differences indicating that the compound synthesized by Venkanna *et al.* [19] is none of the *N*-1- and *N*-2-medoxomil regioisomers (Table 3). Without any reliable proof, the authors drew the structure of the supposed dimedoxomil compound by analogy to the incorrect structures of the starting bromide **2** and the intermediates, *i.e.*, ethyl ester **3** and medoxomil ester **6**, also presented in the paper. Since the structures of compounds **2**, **3** and **6** were reported as tritylated at *N*-1 position, the structure of the unknown compound was also determined as *N*-1-medoxomil substituted. It should be noticed that the structure of *N*-1-medoxomil compound **10** was disclosed in the patent literature [33,34], synthetic article [35] and analytical article [36]. However, synthetic procedures and physicochemical data (m.p., IR, ^1^H- and ^13^C-NMR) allowing preparation, identification and characterization of this compound were not revealed. It is striking that in all of these papers the structural formulas of *N*-tritylated compounds show trityl substituent at wrong *N*-1 position.

**Table 3:** ^1^H Chemical shifts (δ_H_/ppm) for the unknown compound [19] and compounds **9** and **10** of current work recorded in CDCl_3_ solution.

*N*-2-Substituted 9		Unknown Comp. [19]	*N*-1-Substituted 10	
600 MHz	200 MHz	300 MHz	200 MHz	600 MHz
0.98 (3H, t)	0.97 (3H, t)	0.96 (3H, t)	0.95 (3H, t)	0.95 (3H, t)
1.62 (6H, s)	1.62 (6H, s)	1.54 (6H, s)	1.61 (6H, s)	1.61 (6H, s)
1.74 (2H, m)	1.74 (2H, m)	1.66 (2H, m)	1.69 (2H, m)	1.70 (2H, m)
2.08 (3H, s)	2.08 (3H, s)	1.7–1.98 (6H, s)	1.89 (3H, s)	1.89 (3H, s)
2.19 (3H, s)	2.19 (3H, s)		2.13 (3H, s)	2.12 (3H, s)
2.72 (2H, t)	2.72 (2H, t)	2.55 (2H, t)	2.63 (2H, t)	2.63 (2H, t)
4.90 (2H, s)	4.90 (2H, s)	4.40 (2H, s)	4.66 (2H, s)	4.67 (2H, s)
5.44 (2H, s)	5.44 (2H, s)	4.92 (2H, s)	4.91 (2H, s)	4.91 (2H, s)
5.46 (2H, s)	5.45 (2H, s)	5.19 (2H, s)	5.41 (2H, s)	5.40 (2H, s)
	5.61 (1H, br, OH)		5.57 (1H, s, OH)	5.53 (1H, s, OH)
6.82 (2H)	6.82 (2H)	6.98 (2H, d)	6.87 (2H)	6.87 (2H)
7.11 (2H)	7.11 (2H)	7.28 (2H, dd)	7.12 (2H)	7.12 (2H)
7.44 (1H, dd)	7.41–7.61 (3H, m)	7.45–7.80 (4H, m)	7.50–7.77 (4H, m)	7.53 (1H, dd)
7.49 (1H, td)				7.58 (1H, td)
7.56 (1H, td)				7.63 (1H, dd)
7.84 (1H, dd)	7.84 (1H, dd)			7.72 (1H, td)

**Figure 3:**
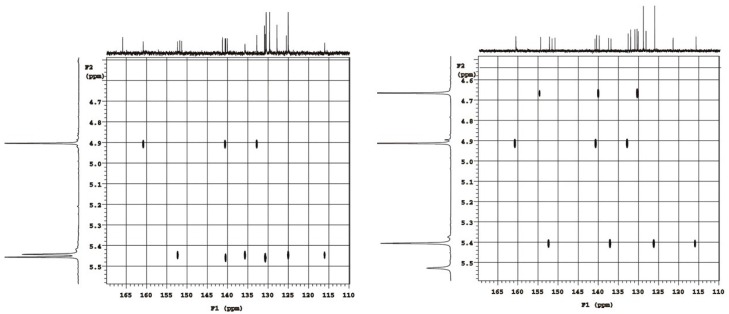
^1^H/^13^C g-HMBC NMR spectrum of *N*-2 substituted dimedoxomil derivative **9** in CDCl_3_ solution (**left**) and *N*-1 substituted dimedoxomil derivative **10** in CDCl_3_ solution (**right**).

The structures of **9** and **10** could be determined only on the basis of the detailed analysis of the multinuclear magnetic resonance results since in their case the attempts of obtaining crystals suitable for SCXRD measurements failed. For these compounds careful analysis of the ^1^H-, ^13^C-, ^15^N-NMR chemical shifts and ^1^H/^13^C (^1^H/^15^N) gradient selected HSQC and HMBC (g-HSQC and g-HMBC) correlations were done. Differentiation of **9** and **10** was possible using cross-peaks coming from ^1^H/^13^C g-HMBC experiment of methylene group of medoxomil substituent at the tetrazole ring. In the case of **9** methylene protons at 5.46 ppm (in CDCl_3_) correlate with carbons at 9.3, 130.7 and 140.5 ppm (Figure 3, left). It means that only three carbons of medoxomil can be identified. Similarly, in the case of **10** (Figure 3, right) methylene protons at 4.67 ppm (in CDCl_3_) correlate with medoxomil carbons at 8.9, 130.3 and 140.0 ppm. However, other additional correlation is observed in 2D *g*-HMBC experiment. The fourth cross-peak at 4.67 ppm/154.6 ppm identifies tetrazole carbon atom and allows **10** to assign the structure of 1,5-disubstituted isomer [10,26,29,30]. Thus, the impurity **9** should be 2,5-disubstituted tetrazole.

**Figure 4:**
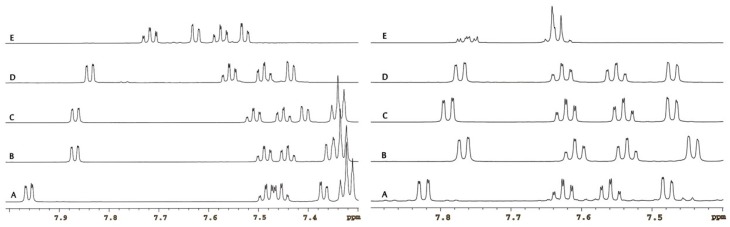
^1^H-NMR spectra of **2** (A), **3** (B), **6** (C), **9** (D) and **10** (E) in CDCl_3_ solutions at 7.3–8.0 ppm range (**left**) and in DMSO-*d*_6_ solutions at 7.4–7.9 ppm range (**right**).

Analyzing the results obtained, we have found another regularity for isomeric 2,5- and 1,5-disubstituted tetrazoles, with tetrazole ring at 2′-position of the biphenyl system. For these compounds most important are some aromatic ^1^H-NMR signals corresponding to the H-3′ and H-5′ protons. Comparison of the corresponding parts of ^1^H-NMR spectra in CDCl_3_ solution (Figure 4, left) and DMSO-*d*_6_ solution (Figure 4, right) indicates that compounds **2**, **3**, **6** and **9** should be structurally similar because they show the same sequence of signals. This is probably caused by very similar inductive effect of the trityl (benzyl-type) and medoxomil (allyl-type) substituents. In contrast, the ^1^H-NMR spectra for **10** differ significantly pointing to other tetrazole ring substitution by the medoxomil substituent.

According to another rule, proton N-C_α_H signal of alkyl group at tetrazole is more deshielded for 2,5-disubstituted isomer compared to its 1,5-disubstituted counterpart [10,26,30,37]. ^1^H-NMR chemical shifts of tetrazole medoxomil N-C_α_H_2_ of **9** are in DMSO at 5.90 ppm and in CDCl_3_ at 5.46 ppm, whereas those of **10** at 5.19 ppm and at 4.67 ppm, respectively. Thus, the rule is fulfilled and **9** is 2,5-disubstituted tetrazole, whereas **10** 1,5-disubstituted. Applying the above rule Kubo *et al.* [10] correctly assigned the ratio of *N*-1- to *N*-2-medoxomil isomer (δ 4.54 and δ 5.41, respectively, in CDCl_3_) in the isolated mixture obtained by alkylation of candesartan derivative CV-11194 with medoxomil chloride (**5**) in DMF in the presence of K_2_CO_3_. The same rule allowed them to assign the ratio of *N*-1- to *N*-2-substituted isomer (δ 5.39 and δ 6.36, respectively, in CDCl_3_) in a mixture of *N*-pivaloyloxymethyl derivatives of CV-11194.

Finally, incomplete sets of the ^15^N chemical signals for **9** and **10** in DMSO coming from ^1^H-^15^N g-HMBC spectra are fully in accordance with data presented earlier for different 2,5- and 1,5-disubstituted tetrazoles [26] (see Table 4).

Based on the above-mentioned results it is possible to state that compounds **2**, **3**, **6** and **9** are 2,5-disubstituted tetrazoles possessing trityl/medoxomil group localized at tetrazole *N*-2 atom. Thus, they belong to the 2*H* tetrazole series. Beyond any doubt, compound **10** is 1,5-disubstituted tetrazole possessing medoxomil group localized at tetrazole *N*-1 atom and it belongs to the 1*H* tetrazole series.

**Table 4:** Comparison of ^15^N-NMR data for some 2,5- and 1,5-disubstituted tetrazoles.

**2,5-Disubstituted Tetrazoles**			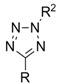			
**Lit. Comp.**	**R**	**R^2^**	***N*-2**	***N*-1**	***N*-3**	***N*-4**
[26] ^a^	Ph-	Me-	−102.1	−79.5	+3.2	−51.8
[26] ^a^	Me-, CP- PhCH_2_- PhCH_2_CH_2_-	Me-	from −105.1 to −99.9	from −81.0 to −71.8	from +1.2 to +6.0	from −53.5 to −46.2
**9 ^b^**	***o*-subst. Ph-**	**Med**	**−99.2**	**−76.7**	**0.0**	**–**
**1,5-Disubstituted Tetrazoles**			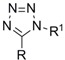			
**Lit. Comp.**	**R**	**R^1^**	***N*-1**	***N*-4**	***N*-2**	***N*-3**
[26] ^a^	Ph-	Me-	−156.2	−50.4	−5.3	+11.4
[26] ^a^	Me-, CP- PhCH_2_- PhCH_2_CH_2_-	Me-	from −154.2 to −151.2	from −58.9 to −44.1	from −7.7 to −2.4	from +9.2 to +14.6
**10 ^b^**	***o*-subst. Ph-**	**Med**	**−149.4**	**–**	**−11.1**	**–**

^a^ in CDCl_3_, relative to external CH_3_NO_2_; ^b^ in DMSO-*d*_6_, relative to CH_3_NO_2_. Me—methyl, Ph—phenyl, CP—cyclopropyl, Med—medoxomil.

## 3. Experimental Section

### 3.1. General Information

Ethyl 4-(1-hydroxy-1-methylethyl)-2-propylimidazole-5-carboxylate (**1**; 97.0%), and 4-chloromethyl-5-methyl-1,3-dioxolen-2-one (medoxomil chloride, **5**; 92.9%) were purchased from Shanghai FWD Chemicals Ltd. [2′-(2-Triphenylmethyl-2*H*-tetrazol-5-yl)biphenyl-4-yl]methyl bromide (**2**; 96.79%) marketed as *N*-(triphenylmethyl)-5-[4′-(bromomethylbiphenyl)-2-yl]tetrazole, *i.e.*, without specified trityl position, was purchased from Huanggang Luban Pharmaceutical Co. Ltd. (Huanggang, China). Potassium dihydrogen phosphate (V) (≥99.5%, for HPLC), phosphoric acid (V) (85 wt % in H_2_O), acetonitrile and tetrahydrofuran of HPLC grade were purchased from Sigma-Aldrich (Munich, Germany) and POCh S.A. (Gliwice, Poland) chemical companies. Deionized water was prepared using MilliQ plus purification system (Millipore, Bradford, PA, USA). Potassium bromide (FT-IR grade), deuterated chloroform and deuterated dimethylsulfoxide were purchased from Merck KGaA (Darmstad, Germany). The course of all reactions and the purity of products were checked by thin-layer chromatography (TLC). Analytical TLC was performed on silica gel DC-Alufolien Kieselgel 60 F_254_ (Merck KGaA, Darmstad, Germany), with mixtures of hexanes, ethyl acetate, dichloromethane and methanol, in various ratios as developing systems. Compounds were visualized by UV light (λ = 254 nm). Column chromatography was carried out on silica gel (Kieselgel 60, 40–63 μm, 230–400 mesh, Merck) with mixtures of ethyl acetate, methanol and dichloromethane in varying ratios as eluents.

### 3.2. Melting Point

Melting points were determined from DSC thermograms performed on a Mettler Toledo DSC822E differential scanning calorimeter (Mettler-Toledo, Columbus, OH, USA).

### 3.3. FT-IR Spectroscopy

FT-IR spectra were taken for KBr pellets on a Nicolet Impact 410 FT-IR spectrophotometer (Thermo Fisher Scientific, Waltham, MA, USA).

### 3.4. Mass Spectrometry

HRMS spectra were recorded on an AMD 604 Inectra Gmbh (AMD Inectra GmbH, Harpstedt, Germany) and a Mariner PE Biosystem ESI-TOF (PerSeptive Biosystems/Applied Biosystems, Waltham, MA, USA) spectrometers.

### 3.5. X-ray Analysis

The X-ray diffraction data for ethyl 4-(1-hydroxy-1-methylethyl)-2-propyl-1-[2′-(2-triphenylmethyl-2*H*-tetrazol-5-yl)biphenyl-4-yl]methyl-1*H*-imidazole-5-carboxylate (**3**) and (5-methyl-2-oxo-1,3-dioxolen-4-yl)methyl 4-(1-hydroxy-1-methylethyl)-2-propyl-1-[2′-(2-triphenylmethyl-2*H*-tetrazol-5-yl)biphenyl-4-yl]methyl-1*H*-imidazole-5-carboxylate (**6**) were collected on an Oxford Diffraction X-calibur (Oxford Diffraction: Wrocław, Poland) with Ruby detector (Mo-Kα radiation; λ = 0.71073 Å). Monocrystals of **3** and **6**, suitable for the XRD experiment, were obtained from acetone-methanol and acetone solution, respectively. The data were collected at 100 K using an Oxford Cryosystem device (Table 5). Data reduction and analysis were carried out with the CrysAlisPro program [38]. The space group was determined using the XPREP program [39]. Structures were solved by direct methods using the SHELXS program and refined using all *F*^2^ data, as implemented by the SHELXL program [40]. In **3**, the imidazole unit reveals two positioned disorder (in ratio of 0.92:0.08) by rotation of 180° around the C11–N71 bond (CH_2_-imidazole N1 bond). In **6**, hydrogen atoms bonded to methyl groups (C85, C100 and C120 *i.e.*, methyl of medoxomil group and two methyls of acetone) are disordered over two positions. The ratio was refined assuming that the sum of both components of the disorder is equal to 1. Non-hydrogen atoms were refined with anisotropic displacement parameters. SIMU, SADI and ISOR restraints were applied for the disordered atom. All H atoms were found in Δρ maps or placed at calculated positions. Before the last cycle of refinement, all H atoms were fixed and were allowed to ride on their parent atoms. For clarity, the minor component of the disordered imidazole units in **3**, and the acetone molecule in **6** solvate, are omitted in Figure 2 and Figure 3, respectively. Crystal structures of **3** and **6** were visualized with Mercury CDS 3.3 software [41]. Crystallographic data for **3** (CCDC 1059380) and **6** acetone solvate (CCDC 1059381) have been deposited with the Cambridge Crystallographic Data Centre. Copies of this information may be obtained free of charge from the Director, CCDC, 12 UNION Road, Cambridge 1EZ. UK (fax: +44-1223-336033; e-mail: deposit@ccdc.cam.ac.uk or http://www.ccdc.cam.ac.uk.

**Table 5:** Summary of crystallographic data and structure refinement for **3** and **6** acetone solvate.

Identification Code	3	6 Acetone Solvate
Chemical formula	C_45_H_44_N_6_O_3_	C_48_H_44_N_6_O_6_·C_3_H_6_O
Molecular weight	716.86 g/mol	858.97 g/mol
Temperature	100(2) K	100(2) K
Wavelength	0.71073 Å	0.71073 Å
Crystal system, space group	Triclinic, P-1	Orthorhombic, *Pca*2_1_
Unit cell dimensions	*a* = 9.531(2) Å	*a* = 13.493(3) Å
	*b* = 10.196(3) Å	*b* = 11.100(3) Å
	*c* = 20.049(3) Å	*c* = 29.124(4) Å
	α = 77.46(3) °	α = 90°
	β = 80.09(3) °	β = 90°
	γ = 78.98(3) °	γ = 90°
Volume	1849.4(8) Å^3^	4362.0(16) Å^3^
*Z*, Calculated density	2, 1.287 mg/m^3^	4, 1.308 mg/m^3^
Absorbtion coefficient	0.082	0.088
*F*(000)	760	1816
Crystal size	0.41 × 0.32 × 0.16 mm	0.71 × 0.49 × 0.20 mm
Theta range for data collection	2.826°–28.857°	2.798°–28.929°
Limiting indices	−12 ≤ *h* ≤ 10	−18 ≤ *h* ≤ 16
	−13 ≤ *k* ≤ 13	−9 ≤ *k* ≤ 15
	−25 ≤ *l* ≤ 26	−38 ≤ *l* ≤ 27
Reflections collected/unique	13,774/8397	13,935/7992
Completeness to Th_max_	0.867	0.887
Absorption correction	None	None
Maximum and minimum transmission	0.987, 0.967 (shelx estimated)	0.983, 0.940 (shelx estimated)
Refinement method	the full-matrix least-squares method using all *F*^2^ data	the full-matrix least-squares method using all *F*^2^ data
Data/restraints/parameters	8397/643/188	7992/577/1
Goodness-of-fit on *F*^2^	1.028	1.046
Final *R*1/*wR2* indices [*I* > 2sigma(*I*)]	0.057/0.110	0.037/0.077
*R1/wR2* indices (all data)	0.093/0.129	0.044/0.081
Largest diff. peak and hole	0.301 and −0.300 e/Å^3^	0.213 and −0.247 e/Å^3^

### 3.6. NMR Spectroscopy

The NMR spectra of all the compounds studied were measured in CDCl_3_ or DMSO-*d*_6_ solutions with a Varian-NMR-vnmrs600 (Varian Inc., Palo Alto, CA, USA) (at 298 K) equipped with a 600 MHz PFG Auto XID (^1^H/^15^N-^31^P 5 mm) indirect probehead. Standard experimental conditions and standard Varian programs (ChemPack 4.1) were used. To assign the structures under consideration, the following 1D and 2D experiments were employed: the 1D selective NOESY and 2D:COSY, ^1^H-^13^C HSQC and ^1^H-^13^C HMBC (in gradient version). The ^15^N-NMR chemical shifts were obtained on a basis of the 2D ^1^H-^15^N gradient selected HMBC experiment, optimized for ^n^*J*(N-H) = 6 Hz. The ^1^H- and ^13^C-NMR chemical shifts were given relative to the TMS signal at 0.0 ppm, whereas neat nitromethane at 0.0 ppm was used as standard for ^15^N-NMR chemical shifts. Concentration of all solutions used for measurements was about 20–30 mg of compounds in 0.6 mL of solvent.

### 3.7. High Performance Liquid Chromatography

Analytical HPLC were performed on a Waters Alliance 2695 system (Waters Chromatography Division, Milford, MA, USA) equipped with a Waters W2489 dual λ absorbance detector, a Waters W2690/5 quaternary pump, autosampler, 100 μL syringe, degasser and column oven. The data were analyzed using the Empower 2 software package (built 2154, Waters Company, Milford, MA, USA).

#### 3.7.1. ***N***-Tritylolmesartan Medoxomil (**6**) Purity Determination

A new method was developed to estimate the purity of *N*-tritylolmesartan medoxomil (**6**) samples. The chromatographic separations were performed on a Kinetex C18 100A (150 × 4.6 mm i.d., particle size 2.6 μm) analytical column manufactured by Phenomenex (Phenomenex Inc., Torrance, CA, USA). The column oven temperature was set at 30 °C and autosampler was kept at 5 °C. All chromatographic runs were carried out in a gradient elution mode, t (min)/B (%): 0/5; 3.5/40; 5/40; 13.5/75; 16/80; 19/95; 25/95; 26/5; 35/5, at a flow rate of 1.0 mL/min. Separations were achieved using a mobile phase consisting of eluent A (0.1% H_3_PO_4_) and a mixture of CH_3_CN–THF (80:20, *v*/*v*) as an eluent B. UV detection was performed at 250 nm and the injection volume was set as 10 μL. Individual stock solutions of standards and samples were prepared in acetonitrile at the concentration of 0.5 mg/mL.

#### 3.7.2. Olmesartan Medoxomil (OM, **7**) Purity Determination

**Figure 5:**
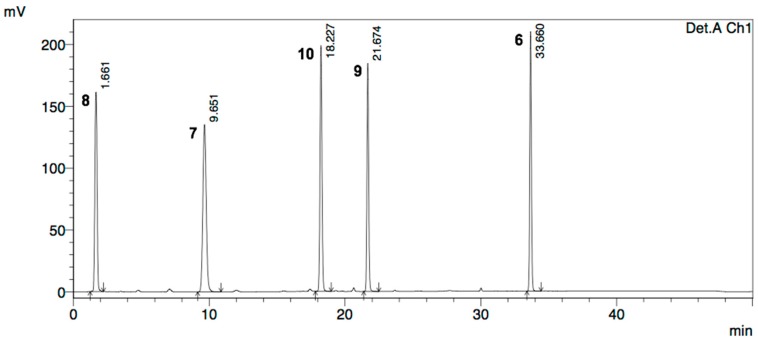
Chromatogram of a reference standard solution containing OM (**7**) and the impurities **6**, **8**, **9**, and **10** at the concentration of 0.1 mg/mL.

Determination of dimedoxomil impurities **9** and **10** in OM (**7**) samples was performed using a method described by pharmacopoeia monographs [17,18]. The method was validated as per the ICH Q2(R1) guidelines, for the parameters like system suitability, specificity, linearity, precision, accuracy, limit of detection (LOD) and limit of quantitation (LOQ) [42,43]. A typical HPLC chromatogram of a reference standard solution comprising the medoxomil ester (**6**), OM (**7**), olmesartan acid (**8**) and regioisomieric impurities **9** and **10** at the concentration of 0.1 mg/mL, used for analyses of OM batches is given in the Figure 5. The chromatographic separations were carried out on a Symmetry C8 (100 × 4.6 mm i.d., particle size 3.5 μm) analytical column manufactured by Waters (Waters Corporation, Milford, MA, USA). The column oven temperature was set at 40 °C and autosampler was kept at 20 °C. All chromatographic runs were carried out in gradient elution mode, t (min)/B (%): 0/25; 10/25; 35/100; 45/100; 46/100; 50/25, at a flow rate of 1.0 mL/min. Separations were achieved using a mobile phase consisting of an eluent A [KH_2_PO_4_ buffer (2.04 g/L, pH 3.4):CH_3_CN, 8:2, *v*/*v*] and an eluent B [KH_2_PO_4_ buffer (2.04 g/L, pH 3.4):CH_3_CN, 2:8, *v*/*v*]. UV detection was performed at 250 nm and the injection volume was set as 10 μL. Individual stock solutions of standards and samples were prepared in acetonitrile at the concentration of 1.0 mg/mL.

### 3.8. Syntheses

The synthesis of OM (**7**) and olmesartan (**8**) from commercially available substrates **1**, **2** and **5** is shown in Scheme 1. The detailed descriptions of the preparation of compounds **3** and **6**–**8** as well as their physical and spectroscopic data are provided in the Supplementary Materials.

#### 3.8.1. (5-Methyl-2-oxo-1,3-dioxolen-4-yl)methyl 4-(1-hydroxy-1-methylethyl)-2-propyl-1-{2′-[2-(5-me-thyl-2-oxo-1,3-dioxolen-4-yl)methyl-2*H*-tetrazol-5-yl]biphenyl-4-yl}methyl-1*H*-imidazole-5-carboxylate (**9**) and (5-methyl-2-oxo-1,3-dioxolen-4-yl)methyl 4-(1-hydroxy-1-methylethyl)-2-propyl-1-{2′-[1-(5-methyl-2-oxo-1,3-dioxolen-4-yl)methyl-1*H*-tetrazol-5-yl]biphenyl-4-yl}methyl-1*H*-imidazole-5-carboxylate (**10**)

K_2_CO_3_ (1.95 g, 14.11 mmol, 2.1 eq), KI (0.67 g, 4.03 mmol, 0.6 eq) and medoxomil chloride (**5**) (2.20 g, 14.78 mmol, 2.2 eq) were added to a suspension of olmesartan (**8**, 3.00 g, 6.72 mmol, 1.0 eq) in DMF (60 mL). After being stirred at room temperature for 22 h, TLC analysis (30% MeOH/AcOEt) indicated the disappearance of the starting material **8** and formation of two reaction products characterized by similar, but different from OM (**7**), polarity. The reaction mixture was diluted with H_2_O (50 mL) and the products were extracted with AcOEt (2 × 50 mL). The combined organic phases were dried over anhydrous MgSO_4_, filtered and concentrated under reduced pressure to give an oily residue. The crude mixture of products (6.36 g, according to the HPLC analysis **9**:**10**—42.56%:57.44%) was purified by column chromatography over silica gel with 50%–100% AcOEt/hexanes gradient elution to afford *N*-2-medoxomil derivative **9** (glassy oil containing ethyl acetate, 0.82 g, R_f_ = 0.76 for 10% MeOH/AcOEt), a mixture of *N*-2- **9** and *N*-1-medoxomil **10** derivatives (2.09 g) and *N*-1-medoxomil derivative **10** (1.36 g, R_f_ = 0.70 for 10% MeOH/AcOEt). Total reaction yield was 95%.

**9**: FT-IR (KBr) ν: 3438, 2968, 2933, 2874, 1828, 1738, 1708, 1675, 1529, 1463, 1433, 1392, 1308, 1227, 1131, 1033, 1066, 765 cm^−1^.

^1^H-NMR (CDCl_3_, 600 MHz) δ: 7.84 (1H, dd, *J* = 7.7 and 1.0 Hz, biphenyl H-3′), 7.56 (1H, td, *J* = 7.5 Hz and 1.4 Hz, biphenyl H-5′), 7.49 (1H, td, *J* = 7.6 and 1.3 Hz, biphenyl H-4′), 7.44 (1H, dd, *J* = 7.7 and 0.9 Hz, biphenyl H-6′), 7.11 (2H, BB′ of AA′BB′ system, biphenyl H-2 and H-6), 6.82 (2H, AA′ of AA′BB′ system, biphenyl H-3 and H-5), 5.46 (2H, s, tetrazole -CH_2_-N<), 5.44 (2H, s, imidazole >N-CH_2_-), 4.90 (2H, s, -CH_2_O-), 2.72 (2H, t, *J* = 7.2 Hz, -CH_2_CH_2_CH_3_), 2.19 (3H, s, medoxomil CH_3_-5 at N-2 of tetrazole), 2.08 (3H, s, medoxomil CH_3_-5 of ester group), 1.74 (2H, m, -CH_2_CH_2_CH_3_), 1.62 (6H, s, -C(OH)(CH_3_)_2_), 0.98 (3H, t, *J* = 7.2 Hz, -CH_2_CH_2_CH_3_).

^13^C-NMR (CDCl_3_, 150 MHz) δ: 165.9 (tetrazole C-5), 160.8 (>C=O), 160.0 (imidazole C-4), 152.3 (imidazole C-2), 151.8 (medoxomil >C=O of ester group), 151.3 (medoxomil >C=O at N-2 of tetrazole), 141.3 (biphenyl C-1′), 140.6 (medoxomil C-4 or C-5 of ester group), 140.5 (medoxomil C-4 or C-5 at N-2 of tetrazole), 140.0 (biphenyl C-1), 135.7 (biphenyl C-4), 132.8 (medoxomil C-4 or C-5 of ester group), 130.8 (biphenyl C-6′), 130.7 (medoxomil C-4 or C-5 at N-2), 130.4 (biphenyl C-5′), 129.6 (2C, biphenyl C-2 and C-6), 127.8 (biphenyl C-4′), 125.5 (biphenyl C-2′), 125.0 (2C, biphenyl C-3 and C-5), 116.0 (imidazole C-5), 70.4 (-C(OH)(CH_3_)_2_), 54.0 (-CH_2_O-), 49.2 (imidazole >N-CH_2_-), 45.1 (tetrazole -CH_2_-N<), 29.2 (2C, -C(OH)(CH_3_)_2_), 29.1 (-CH_2_CH_2_CH_3_), 21.4 (-CH_2_CH_2_CH_3_), 13.8 (-CH_2_CH_2_CH_3_), 9.3 (medoxomil CH_3_-5 at N-2 of tetrazole), 9.3 (medoxomil CH_3_-5 of ester group).

^1^H-NMR (DMSO-*d*_6_, 600 MHz) δ: 7.77 (1H, dd, *J* = 7.8 and 1.2 Hz, biphenyl H-3′), 7.63 (1H, td, *J* = 7.5 and 1.2 Hz, biphenyl H-5′), 7.55 (1H, td, *J* = 7.5 and 1.2 Hz, biphenyl H-4′), 7.47 (1H, dd, *J* = 7.8 and 1.2 Hz, biphenyl H-6′), 7.05 (2H, BB′ of AA′BB′ system, biphenyl H-2 and H-6), 6.84 (2H, AA′ of AA′BB′ system, biphenyl H-3 and H-5), 5.90 (2H, s, tetrazole -CH_2_-N<), 5.42 (2H, s, imidazole >N-CH_2_-), 5.21 (1H, s, -OH), 5.07 (2H, s, -CH_2_O-), 2.65 (2H, t, *J* = 7.2 Hz, -CH_2_CH_2_CH_3_), 2.17 (3H, s, medoxomil CH_3_-5 at N-2 of tetrazole), 2.08 (3H, s, medoxomil CH_3_-5 of ester group), 1.63 (2H, m, -CH_2_CH_2_CH_3_), 1.48 (6H, s, -C(OH)(CH_3_)_2_), 0.90 (3H, t, *J* = 7.2 Hz, -CH_2_CH_2_CH_3_).

^13^C-NMR (DMSO-*d*_6_, 150 MHz) δ: 164.7 (tetrazole C-5), 160.7 (>C=O), 157.6 (imidazole C-4), 151.7 (medoxomil >C=O of ester group), 151.5 (medoxomil >C=O at N-2 of tetrazole), 151.0 (imidazole C-2), 140.9 (biphenyl C-1′), 140.4 (medoxomil C-4 or C-5 of ester group), 140.4 (medoxomil C-4 or C-5 at N-2 of tetrazole), 139.0 (biphenyl C-1), 136.2 (biphenyl C-4), 132.8 (medoxomil C-4 or C-5 of ester group), 131.2 (medoxomil C-4 or C-5 at N-2 of tetrazole), 130.8 (biphenyl C-6′), 130.5 (biphenyl C-5′), 130.3 (biphenyl C-3′), 129.1 (2C, biphenyl C-2 and C-6), 127.8 (biphenyl C-4′), 125.4 (biphenyl C-2′), 125.2 (2C, biphenyl C-3 and C-5), 116.1 (imidazole C-5), 69.6 (-C(OH)(CH_3_)_2_), 54.1 (-CH_2_O-), 48.0 (imidazole >N-CH_2_-), 44.9 (tetrazole -CH_2_-N<), 29.7 (2C, -C(OH)(CH_3_)_2_), 28.3 (-CH_2_CH_2_CH_3_), 20.6 (-CH_2_CH_2_CH_3_), 13.6 (-CH_2_CH_2_CH_3_), 8.7 (medoxomil CH_3_-5 of ester group), 8.6 (medoxomil CH_3_-5 at N-2 of tetrazole).

^15^N-NMR (DMSO-*d*_6_, 60 MHz) δ: 0.0 (tetrazole N-3), −76.7 (tetrazole N-1), −99.2 (tetrazole N-2), −120.0 (imidazole N-3), −211.6 (imidazole N-1).

HRMS (ESI) *m*/*z* 671.2454 (calcd. for C_34_H_35_N_6_O_9_ [M + H]^+^ 671.2466).

**10:** mp 143.84–151.62 °C (AcOEt/hexanes), peak 147.57 °C, heating rate 10.00 °C/min (white crystals).

Lit. mp 126–130 °C (acetone) [19].

FT-IR (pellets, KBr) ν: 3383, 3028, 2967, 2935, 2875, 1836, 1821, 1808, 1743, 1676, 1533, 1467, 1397, 1371, 1313, 1230, 1192, 1151, 1085, 1003, 957, 769.

Lit. IR (KBr) ν: 3385, 2931, 2875, 1816, 1742, 1531 [19].

^1^H-NMR (CDCl_3_, 600 MHz) δ: 7.72 (1H, td, *J* = 7.6 and 1.4 Hz, biphenyl H-5′), 7.63 (1H, dd, *J* = 7.8 and 0.8 Hz, biphenyl H-6′), 7.58 (1H, td, *J* = 7.5 and 1.2 Hz, biphenyl H-4′), 7.53 (1H, dd, *J* = 7.7 and 1.1 Hz, biphenyl H-3′), 7.12 (2H, BB′ of AA′BB′ system, biphenyl H-2 and H-6), 6.87 (2H, AA′ of AA′BB′ system, biphenyl H-3 and H-5), 5.53 (1H, s, -OH), 5.40 (2H, s, imidazole >N-CH_2_-), 4.91 (2H, s, -CH_2_O-), 4.67 (2H, s, tetrazole -CH_2_-N<), 2.63 (2H, t, *J* = 7.2 Hz, -CH_2_CH_2_CH_3_), 2.12 (3H, s, medoxomil CH_3_-5 of ester group), 1.89 (3H, s, medoxomil CH_3_-5 at N-1 of tetrazole, 1.70 (2H, m, -CH_2_CH_2_CH_3_), 1.61 (6H, s, -C(OH)(CH_3_)_2_), 0.95 (3H, t, *J* = 7.2 Hz, -CH_2_CH_2_CH_3_).

^13^C-NMR (150 MHz, CDCl_3_) δ: 160.7 (>C=O), 160.6 (imidazole C-4), 154.6 (tetrazole C-5), 152.4 (imidazole C-2), 151.7 (medoxomil >C=O of ester group), 151.0 (medoxomil >C=O at N-1 of tetrazole), 141.0 (biphenyl C-1′), 140.6 (medoxomil C-4 or C-5 of ester group), 140.0 (medoxomil C-4 or C-5 at N-1 of tetrazole), 137.7 (biphenyl C-1), 137.1 (biphenyl C-4), 132.8 (medoxomil C-4 or C-5 of ester group), 132.2 (biphenyl C-5′), 131.1 (biphenyl C-3′), 130.6 (biphenyl C-6′), 130.3 (medoxomil C-4 or C-5 at N-1 of tetrazole), 129.0 (2C, biphenyl C-2 and C-6), 128.4 (biphenyl C-4′), 126.2 (2C, biphenyl C-3 and C-5), 121.6 (biphenyl C-2′), 115.9 (imidazole C-5), 70.4 (-C(OH)(CH_3_)_2_), 53.9 (-CH_2_O-), 48.9 (imidazole >N-CH_2_-), 39.8 (tetrazole -CH_2_-N<), 29.2 (-CH_2_CH_2_CH_3_), 29.1 (2C, -C(OH)(CH_3_)_2_), 21.2 (-CH_2_CH_2_CH_3_), 13.8 (-CH_2_CH_2_CH_3_), 9.3 (medoxomil CH_3_-5 of ester group), 8.9 (medoxomil CH_3_-5 at N-1 of tetrazole).

^1^H-NMR (DMSO-*d*_6_, 600 MHz) δ: 7.76 (1H, m, Σ_J_ ≈ 16.8 Hz, biphenyl H-5'), 7.64 (3H, m, biphenyl H-3′, H-4′ and H-6′), 7.04 (2H, BB′ of AA′BB′ system, biphenyl H-2 and H-6), 6.85 (2H, AA′ of AA′BB′ system, biphenyl H-3 and H-5), 5.40 (2H, s, imidazole >N-CH_2_-), 5.21 (1H, s, -OH), 5.19 (2H, s, tetrazole -CH_2_-N<), 5.04 (2H, s, -CH_2_O-), 2.58 (2H, t, *J* = 7.2 Hz, -CH_2_CH_2_CH_3_), 2.08 (3H, s, medoxomil CH_3_-5 of ester group), 1.78 (3H, s, medoxomil CH_3_-5 at N-1 of tetrazole), 1.56 (2H, m, -CH_2_CH_2_CH_3_), 1.47 (6H, s, -C(OH)(CH_3_)_2_), 0.86 (3H, t, *J* = 7.2 Hz, -CH_2_CH_2_CH_3_).

^13^C-NMR (DMSO-*d*_6_, 150 MHz) δ: 160.7 (>C=O), 157.6 (imidazole C-4), 154.0 (tetrazole C-5), 151.6 (medoxomil >C=O of medoxomil ester), 151.2 (medoxomil >C=O at N-1 of tetrazole), 151.0 (imidazole C-2), 141.1 (biphenyl C-1′), 140.4 (medoxomil C-4 or C-5 of ester group), 139.7 (medoxomil C-4 or C-5 at N-1 of tetrazole), 137.2 (biphenyl C-1), 137.1 (biphenyl C-4), 132.8 (medoxomil C-4 or C-5 of ester group), 131.8 (biphenyl C-5′), 131.0 (2C, biphenyl C-3′ and C-6′), 130.7 (medoxomil C-4 or C-5 at N-1 of tetrazole), 128.5 (2C, biphenyl C-2 and C-6), 128.0 (biphenyl C-4′), 125.6 (2C, biphenyl C-3 and C-5), 121.6 (biphenyl C-2′), 116.2 (imidazole C-5), 69.6 (-C(OH)(CH_3_)_2_), 54.1 (-CH_2_O-), 47.9 (imidazole >N-CH_2_-), 39.8 (tetrazole -CH_2_-N<), 29.6 (2C, -C(OH)(CH_3_)_2_), 28.2 (-CH_2_CH_2_CH_3_), 20.5 (-CH_2_CH_2_CH_3_), 13.5 (-CH_2_CH_2_CH_3_), 8.7 (medoxomil CH_3_-5 of ester group), 8.2 (medoxomil CH_3_-5 at N-1 of tetrazole).

^15^N-NMR (DMSO-*d*_6_, 60 MHz) δ: −11.1 (tetrazole N-2), −119.6 (imidazole N-3), −149.4 (tetrazole N-1), −212.3 (imidazole N-1).

HRMS (ESI) *m/z* 671.2463 (calcd. for C_34_H_35_N_6_O_9_ [M + H]^+^ 671.2466).

#### 3.8.2. Reaction of Olmesartan (**8**) with Medoxomil Chloride (**5**) According to the Procedure Described by Venkanna *et al*.

K_2_CO_3_ (2.23 g, 16.17 mmol, 1.95 eq), *n*-Bu_4_NBr (cat.) and medoxomil chloride **5** (2.71 g, 18.24 mmol, 2.0 eq) were added to a solution of olmesartan (**8**, 3.7 g, 8.29 mmol, 1.0 eq) in Me_2_CO (40 mL). After heating at reflux for 12 h, TLC analysis (30% MeOH/AcOEt) indicated the disappearance of the starting material **8** and formation of two reaction products characterized by similar, but different from OM (**7**) polarity. The reaction mixture was cooled to room temperature and filtered. The resulting solid was washed with Me_2_CO (10 mL). The combined filtrate and washings were concentrated under reduced pressure, and the residue was portioned between AcOEt (50 mL) and H_2_O (50 mL). The acetate layer was dried over anhydrous Na_2_SO_4_, filtered and concentrated under reduced pressure to give a dark-brown oily residue (6.64 g, according to the HPLC analysis **9**:**10**—56.5%:43.5%). An unsuccessful attempt to purify the crude mixture of *N*-2- (**9**) and *N*-1- (**10**) medoxomil derivatives and acetone condensation products by column chromatography on silica gel with 10% MeOH/CH_2_Cl_2_ elution was made. A dark-brown oily starting material was recovered. After the addition of Me_2_CO, no solid of dimedoxomil derivatives precipitated out of solution.

## 4. Conclusions

Two principal process-related impurities of olmesartan medoxomil API (OM, **7**) formed from the olmesartan acid (**8**) were described. The impurities were identified as isomeric *N*-2- (**9**) and *N*-1- (**10**) (5-methyl-2-oxo-1,3-dioxolen-4-yl)methyl derivatives of OM. The *N*-2-medoxomil impurity **9** is a new compound that has never been synthesized, identified and characterized. The structure of regioisomeric *N*-1-medoxomil impurity **10** was disclosed in the literature. However, either there were no synthetic procedures and physicochemical data, or presented data do not prove the revealed structure. Thus, both compounds **9** and **10** were synthesized, separated and fully characterized. The structures of the impurities, the substrate **2** and the intermediates **3** and **6** in the synthesis of OM were determined by crystallographic and/or spectroscopic methods. The careful analysis of ours and reported analytical data (m.p., IR, NMR and SCXRD) allowed disproving the structural dualism of the tetrazole tritylated compounds **2**, **3** and **6**, existing in the literature data and chemical databases, and undoubtedly established their exact structure as *N*-2-tritylated tetrazole regioisomers. The analysis also evidently shows that the tritylated intermediates of other sartans with 2-(tetrazol-5-yl)biphenyl moiety possess triphenylmethyl substituent at tetrazole *N*-2 position.

## Supplementary Materials

The detailed descriptions of the preparation of **3** and **6**–**8** as well as the spectral data (IR, ^1^H-NMR, ^13^C-NMR, HRMS) and DSC thermograms of the starting bromide **2** and compounds synthesized **3** and **6**–**8** are provided in the Supporting Materials. Supplementary materials can be accessed at: http://www.mdpi.com/1420-3049/20/12/19762/s1.
