# Supplementary material for: Synthesis and Physicochemical Characterization of the Process-Related Impurities of Olmesartan Medoxomil. Do 5-(Biphenyl-2-yl)-1-triphenylmethyltetrazole Intermediates in Sartan Syntheses Exist?
Source: Molecules. 2015 Dec 1;20(12):21346–63. doi: 10.3390/molecules201219762 (PMC6332230; doi:10.3390/molecules201219762)
Supplement: Supplementary file 1 [file molecules-20-19762-s001.pdf]

Supplementary Materials: Synthesis and Physicochemical Characterization of the Process-Related Impurities of Olmesartan Medoxomil. Do 5-(Biphenyl-2-yl)-1-triphenylmethyltetrazole Intermediates in Sartan Syntheses Exist?

Iwona Dams, Anna Ostaszewska, Maria Puchalska, Justyna Chmiel, Piotr Cmoch, Iwona Bujak, Agata Białońska and Wojciech J. Szczepek

Table of contents

	Page
Syntheses	S1
Table S1	S2
Table S2	S2
Table S3	S3
Spectral data (NMR, IR, HRMS) and DSC thermograms of the bromide **2** and compounds synthesized	S10
^{1}H-NMR spectra of compounds **2**, **3**, **6**, **7**, **9** and **10** in $CDCl_3$ solutions	S10
^{1}H-NMR spectra of compounds **2**, **3** and **6-10** in DMSO-d_6 solutions	S18
^{13}C-NMR spectra of compounds **2**, **3**, **6**, **7**, **9** and **10** in $CDCl_3$ solutions	S25
^{13}C-NMR spectra of compounds **2**, **3** and **6–10** in DMSO-d_6 solutions	S31
^{1}H/^{13}C g-HMBC NMR spectra of dimedoxomil derivatives **9** and **10**	S38
^{1}H/^{15}N g-HMBC NMR spectra of dimedoxomil derivatives **9** and **10**	S40
IR spectra of compounds **2**, **3** and **6–10**	S42
HRMS spectra of compounds **3** and **6–10**	S49
DSC thermograms of compounds **2**, **3**, **6–8** and **10**	S55
Abbreviations used in the text	S61
References	S61

Syntheses

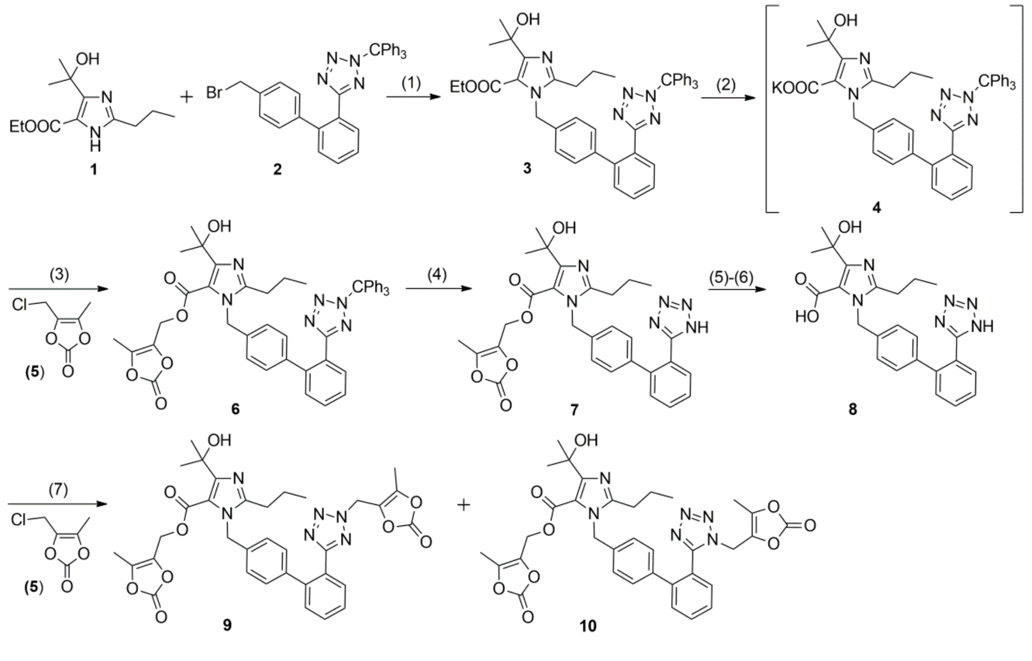

Scheme S1. Synthesis of OM (**7**) and the impurities **9** and **10**: (1) K_2CO_3, KI, DMF, 24 h at r.t., 83%; (2) KOH, DMF, 54–56 °C for 22 h; (3) K_2CO_3, KI, DMF, 22 h at r.t., 96%; (4) H_2SO_4–H_2O, Me_2CO, 50–55 °C for 2 h; 80%; (5) NaOH, MeOH, 24 h at r.t.; (6) AcOH, H_2O; 96%; (7) K_2CO_3, KI, DMF, 22 h at r.t.

[2′-(2-Triphenylmethyl-2*H*-tetrazol-5-yl)biphenyl-4-yl]methyl bromide (2)

The IR and NMR spectra, as well as DSC thermogram, were recorded for commercial sample of the bromide **2**.

M.p. 155.10–160.57 °C, peak 157.73 °C, heating rate 10.00 °C/min (white crystals). Lit. m.p.: 150–151 [1], 141.0–142.3 [2], 140–142 [3], 137.8 [4], 137–138 [5], 136–138 [6,7], 135–138 [8–10], 135–137 [11,12], 134–137 [13], 129.5–133.0 [14].

FT-IR (KBr) ν: 3445, 3054, 3028, 1489, 1463, 1445, 1431, 1406, 1230, 1204, 1186, 1156, 1026, 1006, 768, 749, 697, 678, 640, 609 cm^{-1}.

^{1}H-NMR ($CDCl_3$, 600 MHz) δ: 7.96 (1H, dd, *J* = 7.8 and 1.2 Hz, biphenyl H-3′), 7.48 (1H, td, *J* = 7.5 and 1.2 Hz, biphenyl H-5′), 7.45 (1H, td, *J* = 7.5 and 1.2 Hz, biphenyl H-4′), 7.37 (1H, dd, *J* = 7.8 and 1.2 Hz, biphenyl H-6′), 7.32 (3H, m, *para*-H of -CPh_3), 7.25 (6H, m, *meta*-H of -CPh_3), 7.11 (2H, AA′ of AA′BB′ system, biphenyl H-3 and H-5), 7.08 (2H, BB′ of AA′BB′ system, biphenyl H-2 and H-6), 6.90 (6H, m, *ortho*-H of -CPh_3), 4.37 (2H, s, -CH_2Br). Lit. ^{1}H-NMR data in $CDCl_3$ are presented in Table S1.

Table S1. Literature ^{1}H-NMR data for [2′-(*N*-triphenylmethyltetrazol-5-yl)biphenyl-4-yl]methyl bromides (**2**) in $CDCl_3$ compared with data from current work (CW).

CW, 600 MHz	Lit. [2]	Lit. [4]	Lit. [6], 400 MHz	Lit. [15], 300 MHz
**4.37** (2H, s)	**4.37** (2H, s)	**4.40** (2H, s)	**4.53** (2H, s)	**4.52** (3H, s)
6.90 (6H, m)	6.88–6.91 (7H, m)		7.02–7.04 (6H, m)	7.13–7.32 (15H, m)
7.08 (2H)	7.09–7.10 (3H, m)		7.20 (2H, d)	
7.11 (2H)		7.15 (2H, d)	7.28 (2H, d)	
7.25 (6H, m)	7.24–7.38 (10H, m)	7.30–7.70 (21H, m)	7.33–7.53 (10H, m)	7.26 (2H, d)
7.32 (3H, m)				7.39 (2H, d)
7.37 (1H, dd)				7.6–7.8 (4H, m)
7.45 (1H ,td)	7.43–7.51 (2H, m)		7.58–7.66 (2H, m)	
7.48 (1H, td)				
7.96 (1H, dd)	7.95–7.97 (1H, m)		8.01 (1H, d)	

^{1}H-NMR (DMSO-d_6, 600 MHz) δ: 7.82 (1H, dd, *J* = 7.2 and 1.2 Hz, biphenyl H-3′), 7.63 (1H, td, *J* = 7.8 and 1.2 Hz, biphenyl H-5'), 7.56 (1H, td, *J* = 7.8 and 1.2 Hz, biphenyl H-4′), 7.48 (1H, dd, *J* = 7.2 and 1.2 Hz, biphenyl H-6′), 7.38 (3H, m, *para*-H of -CPh_3), 7.34 (6H, m, *meta*-H of -CPh_3), 7.29 (2H, AA′ of AA′BB′ system, biphenyl H-3 and H-5), 7.06 (2H, BB′ of AA′BB′ system, biphenyl H-2 and H-6), 6.85 (6H, m, *ortho*-H of -CPh_3). Lit. ^{1}H-NMR data in DMSO-d_6 are presented in Table S2.

Table S2. Literature ^{1}H-NMR data for [2′-(*N*-triphenylmethyltetrazol-5-yl)biphenyl-4-yl]methyl bromides (**2**) in DMSO-d_6 compared with data from current work (CW).

CW, 600 MHz	Lit. [1], 300 MHz	Lit. [16], 400 MHz	Lit. [17], 400 MHz
**4.65** (2H, s)	**4.48** (2H, s)	**4.65** (2H, s)	**4.61** (2H, s)
6.85 (6H, m)	7.00–7.84 (23H, m)	6.84 (6H, d)	6.80 (6H, d)
7.06 (2H)		7.06 (2H, d)	7.01 (2H, d)
7.29 (2H)		7.28 (2H, d)	7.24 (2H, d)
7.34 (6H, m)		7.32–7.38 (9H, m)	7.28–7.35 (9H, m)
7.38 (3H, m)			
7.48 (1H, dd)		7.42–7.64 (3H, m)	7.43–7.45 (1H, dd)
7.56 (1H, td)			7.50–7.56 (1H, td)
7.63 (1H, td)			7.58–7.60 (1H, td)
7.82 (1H, dd)		7.78–7.84 (1H, m)	7.77–7.79 (1H, dd)

^{13}C-NMR ($CDCl_3$, 150 MHz) δ: 163.8 (tetrazole C-5), 141.5 (biphenyl C-1′), 141.3 (biphenyl C-1), 141.1 (3C, *ipso*-C of -CPh_3), 136.2 (biphenyl C-4), 130.6 (biphenyl C-6′), 130.3 (6C, *ortho*-C of -CPh_3), 130.3 (biphenyl C-3′), 130.0 (biphenyl C-5′), 129.6 (2C, biphenyl C-2 and C-6), 128.5 (2C, biphenyl C-3 and C-5), 128.2 (3C, *para*-C of -CPh_3), 127.7 (biphenyl C-4), 127.6 (6C, *meta*-C of -CPh_3), 126.3 (biphenyl C-2′), 83.0 (-CPh_3), 33.2 (-CH_2Br). Lit. ^{13}C-NMR data in $CDCl_3$ are presented in Table S3.

^{13}C-NMR (150 MHz, DMSO-d_6) δ: 163.3 (tetrazole C-5), 141.0 (biphenyl C-1′), 140.7 (3C, *ipso*-C of -CPh_3), 140.1 (biphenyl C-1), 136.6 (biphenyl C-4), 130.6 (biphenyl C-6′), 130.5 (biphenyl C-5′), 130.3 (biphenyl C-3′), 129.6 (6C, *ortho*-C of -CPh_3), 129.1 (2C, biphenyl C-2 and C-6), 129.0 (2C, biphenyl C-3 and C-5), 128.3 (3C, *para*-C of -CPh_3 group), 127.9 (biphenyl C-4′), 127.8 (6C, *meta*-C of -CPh_3), 125.6 (biphenyl C-2′), 82.3 (-CPh_3), 34.0 (-CH_2Br). Lit. ^{13}C-NMR data in DMSO-d_6 are presented in Table S3.

Table S3. Literature ^{13}C-NMR data for [2′-(*N*-triphenylmethyltetrazol-5-yl)biphenyl-4-yl]-methyl bromides (**2**) in $CDCl_3$ and DMSO-d_6 compared with data from current work (CW).

		**$CDCl_3$**			**DMSO-d_6**	
**CW 150 MHz**	**Lit. [2]**	**Lit. [6] 160 MHz**	**Lit. [15]**	**Lit. [4]**	**CW 150 MHz**	**Lit. [1] 75 MHz**
33.2	33.1	33.2	33.5	38.2	34.0	38.0
**83.0**	**82.9**	**83.0**	**95.3**	**62.1**	**82.3**	**64.6**
126.3	126.2	127.7	118.0	126.2	125.6	111.9
127.6	127.4	127.9	126.2	127.9	127.8	127.4
127.7	127.5	128.2	128.0	128.2	127.9	127.4
128.2	127.5	128.5	128.1	128.9	128.3	127.9
128.5	127.6	129.4	128.3	129.6	129.0	128.2
129.6	127.8	129.6	128.7	131.0	129.1	128.4
130.0	128.1	130.0	129.0	135.9	129.6	129.0
130.3	128.1	130.2	129.4	136.2	130.3	129.2
130.3	128.4	130.3	129.4	137.8	130.5	129.4
130.6	129.5	130.6	135.2	138.0	130.6	129.5
136.2	129.8	138.2	135.4	143.2	136.6	135.1
141.1	130.1	141.1	136.8		140.1	135.4
141.3	130.2	**163.8**	141.8		140.7	136.0
141.5	130.2		142.5		141.0	136.8
**163.8**	130.3		**163.6**		**163.3**	141.0
	130.5					144.0
	136.1					151.4
	141.0					
	141.1					
	141.2					
	141.4					
	146.8					
	**163.7**					

Ethyl 4-(1-hydroxy-1-methylethyl)-2-propyl-1-[2′-(2-triphenylmethyl-2*H*-tetrazol-5-yl)-biphenyl-4-yl]methyl-1*H*-imidazole-5-carboxylate (3)

The bromide **2** (58.08 g, 104.18 mmol, 1.0 eq), K_2CO_3 (18.0 g, 130.23 mmol, 1.25 eq) and KI (0.87 g, 5.21 mmol, 0.05 eq) were added to a solution of the ethyl ester **1** (25.03 g, 104.18 mmol, 1.0 eq) in DMF (230 mL). The reaction mixture was vigorously stirred at room temperature for 24 h. H_2O (345 mL) was added dropwise and the resulting suspension was allowed to cool to room

temperature while stirring. The solid precipitated was filtered off and washed with H_2O (230 mL). The wet-cake was macerated in Me_2CO (220 mL) under reflux for 30 min. The mixture was allowed to cool to room temperature. The solid was filtered off, washed with Me_2CO (50 mL) and dried in air at room temperature to afford the ethyl ester **3** (62.06 g, 83%).

M.p. 168.37–171.86 °C, peak 168.70 °C, heating rate 10.00 °C/min (white crystals). Lit. m.p.: 167–168 °C (diisopropyl ether, dec.) [18], 165–166 °C (diisopropyl ether-hexane, dec.) [19], 165–169 °C (water) [20,21], 161 °C (isopropanol) [22], 164–167 °C (N,N-dimethylacetamide-water) [23].

FT-IR (KBr) ν: 3401, 3055, 2962, 2934, 2873, 1737, 1701, 1665, 1604, 1525, 1492, 1470, 1446, 1409, 1376, 1290, 1177, 1142, 1056, 1033, 757, 746, 699, 640 cm^{-1}. Lit. IR ν: (KBr) 3407, 3056, 2977, 2935, 1961, 1702, 1666, 1603, 1470, 1290, 1177, 1033, 881, 756, 699 cm^{-1} [22]; (KBr) 3403, 3088, 3055, 3026, 1778, 1701, 1666, 1524, 1492, 1469, 1446, 1409, 1396, 1376, 1335, 1176, 1142, 1055, 1032, 928, 778, 746 cm^{-1} [23]; 1666, 1525, 1291, 1177, 881, 756, 699, 640 cm^{-1} [20,21].

^{1}H-NMR ($CDCl_3$, 600 MHz) δ: 7.87 (1H, dd, *J* = 7.8 and 1.2 Hz, biphenyl H-3′), 7.49 (1H, td, *J* = 7.8 and 1.2 Hz, biphenyl H-5′), 7.44 (1H, td, *J* = 7.5 and 1.2 Hz, biphenyl H-4′), 7.36 (1H, dd, *J* = 7.2 and 1.2 Hz, biphenyl H-6′), 7.34 (3H, m, *para*-H of $-CPh_3$), 7.26 (6H, *meta*-H of $-CPh_3$), 7.10 (2H, BB' of AA′BB′ system, biphenyl H-2 and H-6), 6.96 (6H, m, *ortho*-H of $-CPh_3$), 6.72 (2H, AA′ of AA′BB′ system, biphenyl H-3 and H-5), 5.80 (1H, s, -OH), 5.35 (2H, s, >N-CH_2-), 4.13 (2H, q, *J* = 7.2 Hz, $-OC\underline{H}_2CH_3$), 2.51 (2H, t, *J* = 7.2 Hz, $-C\underline{H}_2CH_2CH_3$), 1.67 (2H, m, $-CH_2C\underline{H}_2CH_3$), 1.65 (6H, s, $-C(OH)(C\underline{H}_3)_2$), 1.08 (3H, t, *J* = 7.2 Hz, $-OCH_2C\underline{H}_3$), 0.88 (3H, t, *J* = 7.2 Hz, $-CH_2CH_2C\underline{H}_3$).

Lit. ^{1}H-NMR ($CDCl_3$) δ: (400 MHz) 7.85–7.88 (1H, m), 7.26–7.47 (12H, m), 7.08–7.11 (2H, m), 6.94–6.97 (6H, m), 6.07–6.74 (2H, m), 5.35 (2H, s), 4.07–4.17 (2H, q, *J* = 13.6 Hz), 2.48–2.55 (2H, t, *J* = 14.4 Hz), 1.70–1.82 (2H, m), 1.64 (6H, s), 1.04–1.11 (3H, t, *J* = 13.6 Hz), 0.84–0.91 (3H, t, *J* = 13.6 Hz) [22]; 7.8–8.1 (1H, m), 6.7–7.61 (22H, m), 5.78 (1H, s), 5.38 (2H, s), 4.12 (2H, q), 2.52 (2H, t), 1.64 (6H, s), 1.5–1.8 (2H, m), 1.08 (3H, t), 0.88 (3H, t) [24]; 7.8–8.1 (1H, m), 6.7–7.6 (22H, m), 5.78 (1H, s), 5.38 (2H, s), 4.12 (2H, q, *J* = 7.0 Hz), 2.52 (2H, t, *J* = 8.0 Hz), 1.64 (6H, s), 1.5-1.8 (2H, m), 1.08 (3H, t, *J* = 7.0 Hz), 0.88 (3H, t, *J* = 7.0 Hz) [18].

^{1}H-NMR (DMSO-*d*$_6$, 600 MHz) δ: 7.77 (1H, dd, *J* = 7.2 and 1.2 Hz, biphenyl H-3′), 7.61 (1H, td, *J* = 7.8 and 1.2 Hz, biphenyl H-5′), 7.54 (1H, td, *J* = 7.5 and 1.2 Hz, biphenyl H-4′), 7.44 (1H, brd d, *J* = 7.2 Hz, biphenyl H-6′), 7.38 (3H, m, *para*-H of $-CPh_3$), 7.33 (6H, m, *meta*-H of $-CPh_3$), 7.06 (2H, BB′ of AA′BB′ system, biphenyl H-2 and H-6), 6.90 (6H, m, *ortho*-H of $-CPh_3$), 6.84 (2H, AA′ of AA′BB′ system, biphenyl H-3 and H-5), 5.42 (1H, s, -OH), 5.41 (2H, s, >N-CH_2-), 4.08 (2H, q, *J* = 7.2 Hz, $-OC\underline{H}_2CH_3$), 2.47 (2H, t, *J* = 7.2 Hz, $-C\underline{H}_2CH_2CH_3$), 1.55 (2H, m, $-CH_2C\underline{H}_2CH_3$), 1.50 (6H, s, $-C(OH)(C\underline{H}_3)_2$), 1.00 (3H, t, *J* = 7.2 Hz, $-OCH_2C\underline{H}_3$), 0.79 (3H, t, *J* = 7.2 Hz, $-CH_2CH_2C\underline{H}_3$).

Lit. ^{1}H-NMR (DMSO-*d*$_6$, 300 MHz) δ: 6.83–7.75 (23H, m), 5.41 (3H, d), 4.06 (2H, q), 2.45 (2H, d), 1.52 (8H, m), 0.99 (3H, t), 0.77 (3H, t) [23].

^{13}C-NMR ($CDCl_3$, 150 MHz) δ: 164.1 (tetrazole C-5), 161.6 (>C=O), 158.7 (imidazole C-4), 151.3 (imidazole C-2), 141.5 (biphenyl C-1′), 141.3 (3C, *ipso*-C of $-CPh_3$), 140.4 (biphenyl C-1), 135.6 (biphenyl C-4), 130.7 (biphenyl C-6′), 130.4 (biphenyl C-3′), 130.2 (6C, *ortho*-C of $-CPh_3$), 129.9 (biphenyl C-5′), 129.7 (2C, biphenyl C-2 and C-6), 128.3 (3C, *para*-C of $-CPh_3$), 127.6 (6C, *meta*-C of $-CPh_3$), 127.6 (biphenyl C-4′), 126.3 (biphenyl C-2′), 124.8 (2C, biphenyl C-3 and C-5), 116.9 (imidazole C-5), 82.9 ($-CPh_3$), 70.3 ($-\underline{C}(OH)(CH_3)_2$), 61.2 ($-O\underline{C}H_2CH_3$), 48.8 (>N-$\underline{C}H_2$-), 29.4 (2C, $-C(OH)(\underline{C}H_3)_2$), 29.3 ($-\underline{C}H_2CH_2CH_3$), 21.3 ($-CH_2\underline{C}H_2CH_3$), 13.9 ($-OCH_2\underline{C}H_3$), 13.8 ($-CH_2CH_2\underline{C}H_3$).

^{13}C-NMR (DMSO-*d*$_6$, 150 MHz) δ: 163.5 (tetrazole C-5), 161.5 (>C=O), 157.0 (imidazole C-4), 150.5 (imidazole C-2), 141.1 (biphenyl C-1′), 140.8 (3C, *ipso*-C of $-CPh_3$), 139.0 (biphenyl C-1), 136.3 (biphenyl C-4), 130.6 (biphenyl C-6′), 130.5 (biphenyl C-5′), 130.3 (biphenyl C-3′), 129.5 (6C, *ortho*-C of

$-CPh_3$), 129.2 (2C, biphenyl C-2 and C-6), 128.3 (3C, *para*-C of $-CPh_3$), 127.8 (6C, *meta*-C of $-CPh_3$), 127.7 (biphenyl C-4′), 125.7 (biphenyl C-2′), 125.3 (2C, biphenyl C-3 and C-5), 116.8 (imidazole C-5), 82.2 ($-CPh_3$), 69.6 ($-\underline{C}(OH)(CH_3)_2$), 60.8 ($-O\underline{C}H_2CH_3$), 47.9 ($>N-\underline{C}H_2-$), 29.7 (2C, $-C(OH)(\underline{C}H_3)_2$), 28.2 ($-\underline{C}H_2CH_2CH_3$), 20.4 ($-CH_2\underline{C}H_2CH_3$), 13.6 ($-CH_2CH_2\underline{C}H_3$), 13.5 ($-OCH_2\underline{C}H_3$).

HRMS (ESI) *m*/*z* 717.3550 (calcd. for $C_{45}H_{45}N_6O_3$ $[M + H]^+$ 717.3553).

Crystal data for 3: $C_{45}H_{44}N_6O_3$, M = 716.86, triclinic, P-1, a = 9.531(2) Å, b = 10.196(3) Å, c = 20.049(3) Å, α = 77.46(3)°, β = 80.09(3)°, γ = 78.98(3)°, V = 1849.4(8) $Å^3$, Z = 2, D_c = 1.287 $Mg{\cdot}m^{-3}$, T = 100(2) K, R = 0.066, wR = 0.144 [5777 reflections with $I > 2\sigma(I)$] for 497 variables. CCDC 1059380.

(5-Methyl-2-oxo-1,3-dioxolen-4-yl)methyl 4-(1-hydroxy-1-methylethyl)-2-propyl-1-[2′-(2-triphenylmethyl-2*H*-tetrazol-5-yl)biphenyl-4-yl]methyl-1*H*-imidazole-5-carboxylate (6)

KOH (7.72 g, 137.50 mmol, 1.55 eq) was added to a suspension of the ethyl ester **3** (63.59 g, 88.71 mmol, 1.0 eq) in DMF (254 mL). After heating at 54–56 °C for 22 h, TLC analysis (50% AcOEt/hexanes, R_f = 0.48 for **3**) indicated the disappearance of the starting material. The reaction mixture was cooled to 40 °C. K_2CO_3 (12.87 g, 93.15 mmol, 1.05 eq) and KI (4.42 g, 26.61 mmol, 0.3 eq) were added to a solution of potassium salt **4**, followed by dropwise addition of the chloride **5** (26.35 g, 177.42 mmol, 2.0 eq). After being stirred at room temperature for 20 h, TLC analysis (30% MeOH/AcOEt, R_f = 0.34 for **4**) indicated the disappearance of the potassium salt **4.** The reaction mixture was diluted with CH_2Cl_2 (200 mL), chilled H_2O (400 mL) and the resulting layers were separated. The aqueous layer was extracted with CH_2Cl_2 (2 × 150 mL). The combined organic phases were washed with brine (400 mL), dried over anhydrous $MgSO_4$ (50 g), filtered and concentrated by evaporation under reduced pressure to give the crude **6** as a light brown oil. A mixture of *i*-PrOH–H_2O (2:1; 300 mL) was added slowly to the oily residue. The resulting mixture was stirred at room temperature for 30 min. and then filtered. The solid obtained was washed with a mixture of *i*-PrOH–H_2O (2:1; 60 mL) and dried in air at room temperature to give the medoxomil ester **6** (68.06 g, 96% yield). HPLC purity: **2** (0.05%), **3** (0.08%), **6** (97.11%), **8** (0.13%), **9** (0.56%), **10** (0.56%) and the sum of other impurities (1.51%).

M.p. 99.86–106.59 °C, peak 103.13 °C, heating rate 10.00 °C/min (white crystals). Lit. m.p.: 104–106 °C (diisopropyl ether) [23], 103–104 °C (acetonitrile) [25], 102–104 °C (ethyl acetate-diisopropyl ether) [19], 98–100 °C (diisopropyl ether, dec.) [18].

FT-IR (KBr) ν: 3395, 3059, 2969, 2929, 2873, 1818, 1804, 1737, 1679, 1527, 1493, 1467, 1446, 1435, 1394, 1308, 1283, 1231, 1185, 1146, 1058, 1004, 770, 762, 749, 698 cm^{-1}. Lit. IR ν: (KBr) 3398, 3059, 3027, 2873, 1819, 1805, 1737, 1707, 1527, 1492, 1465, 1393, 1357, 1256, 1170, 1094, 1004, 726, 678 cm^{-1} [23]; 3408, 1818, 1805, 1741, 1681, 1529, 1147, 1003, 699 cm^{-1} [26]; (KBr) 3420, 1825, 1738, 1707, 1678 cm^{-1} [19]; (KBr) 3408, 1819 cm^{-1} [25].

^1H-NMR ($CDCl_3$, 600 MHz) δ: 7.87 (1H, dd, J = 7.8 and 1.2 Hz, biphenyl H-3′), 7.51 (1H, td, J = 7.8 and 1.2 Hz, biphenyl H-5′), 7.45 (1H, td, J = 7.5 and 1.2 Hz, biphenyl H-4′), 7.41 (1H, dd, J = 7.2 and 1.2 Hz, biphenyl H-6′), 7.34 (3H, m, *para*-H of $-CPh_3$), 7.27 (6H, m, *meta*-H of $-CPh_3$), 7.10 (2H, BB′ of AA′BB′ system, biphenyl H-2 and H-6), 6.97 (6H, m, *ortho*-H of $-CPh_3$), 6.69 (2H, AA′ of AA′BB′ system, biphenyl H-3 and H-5), 5.59 (1H, s, -OH), 5.30 (2H, s, $>N-CH_2-$), 4.71 (2H, s, $-CH_2O-$), 2.54 (2H, t, J = 7.2 Hz, $-C\underline{H_2}CH_2CH_3$), 1.97 (3H, s, medoxomil CH_3-5), 1.69 (2H, m, $-CH_2C\underline{H_2}CH_3$), 1.63 (6H, s, $-C(OH)(C\underline{H_3})_2$), 0.90 (3H, t, J = 7.2 Hz, $-CH_2CH_2C\underline{H_3}$).

Lit. ^1H-NMR ($CDCl_3$) δ: 7.87 (1H, d, J = 7.5 Hz), 6.90–7.52 (20H, m), 6.68 (2H, d, J = 7.5 Hz), 5.61 (1H, s), 5.30 (2H, s), 4.70 (2H, s), 2.54 (2H, t, J = 8.0 Hz), 1.97 (3H, s), 1.6–1.75 (2H, m), 1.62 (6H, s), 0.89 (3H, t, J = 7.5 Hz) [18,19]; (200 MHz) 7.87 (1H, d), 6.90–7.52 (20H, m), 6.68 (2H, d), 5.61 (1H, s), 5.30 (2H, s),

4.70 (2H, s), 2.54 (2H, t), 1.97 (3H, s), 1.6–1.75 (2H, m), 1.62 (6H, s), 0.87 (3H, t) [24]; 7.23–7.82 (15H, m), 6.98 (2H, d, *J* = 8.2 Hz), 6.82 (4H, d, *J* = 8.2 Hz), 6.75 (2H, d, *J* = 8.2 Hz), 5.31 (2H, s), 5.22 (1H, s), 5.02 (2H, s), 2.30–2.60 (2H, m), 2.0 (3H, s), 1.56 (6H, s), 1.41–1.60 (2H, m), 0.85 (3H, t, *J* = 7.2 Hz) [25].

^{1}H-NMR (DMSO-d_6, 600 MHz) δ: 7.79 (1H, dd, *J* = 7.2 and 1.2 Hz, biphenyl H-3′), 7.62 (1H, td, *J* = 7.8 and 1.2 Hz, biphenyl H-5′), 7.54 (1H, td, *J* = 7.5 and 1.2 Hz, biphenyl H-4′), 7.47 (1H, brd d, *J* = 7.2 Hz, biphenyl H-6′), 7.38 (3H, m, *para*-H of -CPh_3), 7.32 (6H, m, *meta*-H of -CPh_3), 7.02 (2H, BB′ of AA′BB′ system, biphenyl H-2 and H-6), 6.88 (6H, m, *ortho*-H of -CPh_3), 6.78 (2H, AA′ of AA′BB′ system, biphenyl H-3 and H-5), 5.36 (2H, s, >N-CH_2-), 5.26 (1H, s, -OH), 5.01 (2H, s, -CH_2O-), 2.44 (2H, t, *J* = 7.2 Hz, -$C\underline{H}_2CH_2CH_3$), 2.03 (3H, s, medoxomil CH_3-5), 1.54 (2H, m, -$CH_2C\underline{H}_2CH_3$), 1.51 (6H, s, -$C(OH)(C\underline{H}_3)_2$), 0.76 (3H, t, *J* = 7.2 Hz, -$CH_2CH_2C\underline{H}_3$).

Lit. ^{1}H-NMR (DMSO-d_6) δ: (400 MHz) 7.78 (1H, d, *J* = 6.8 Hz), 7.63 (1H, m), 7.54 (1H, t, *J* = 6.8 and 7.6 Hz), 7.47 (1H, d, *J* = 7.6 Hz), 7.35 (9H, m), 7.01 (2H, d, *J* = 8.4 Hz), 6.88 (6H, d, *J* = 6.6 Hz), 6.77 (2H, d, *J* = 8.0 Hz), 5.36 (2H, s), 5.26 (1H, s), 5.0 (2H, s), 2.50 (2H, t, *J* = 1.6 and 1.6 Hz), 2.02 (3H, s), 1.53 (2H, m), 1.50 (6H, s), 0.75 (3H, t, *J* = 7.2 and 7.6 Hz) [27]; (300 MHz) 7.29–7.78 (13H, m), 7.02 (2H, d), 6.87 (6H, d), 6.77 (2H, d), 5.35 (2H, s), 5.25 (1H, s), 5.00 (2H, s), 2.43 (2H, t), 2.02 (3H, s), 1.54 (2H, m), 1.49 (6H, m), 0.75 (3H, t), [23].

^{13}C-NMR ($CDCl_3$, 150 MHz) δ: 164.2 (tetrazole C-5), 160.7 (>C=O), 160.5 (imidazole C-4), 152.1 (imidazole C-2), 151.8 (medoxomil >C=O), 141.3 (biphenyl C-1′), 141.3 (3C, *ipso*-C of -CPh_3), 140.4 (biphenyl C-1), 140.4 (medoxomil C-4 or C-5), 135.5 (biphenyl C-4), 132.8 (medoxomil C-4 or C-5), 130.5 (biphenyl C-6′), 130.4 (biphenyl C-3′), 130.2 (6C, *ortho*-C of -CPh_3), 130.1 (biphenyl C-5′), 129.7 (2C, biphenyl C-2 and C-6), 128.3 (3C, *para*-C of -CPh_3), 127.7 (7C, biphenyl C-4′ and *meta*-C of -CPh_3), 126.3 (biphenyl C-2′), 124.4 (2C, biphenyl C-3 and C-5), 115.9 (imidazole C-5), 82.9 (-CPh_3), 70.4 (-$\underline{C}(OH)(CH_3)_2$), 53.7 (-$CH_2O$-), 49.2 (>N-$\underline{C}H_2$-), 29.3 (-$\underline{C}H_2CH_2CH_3$), 29.2 (2C, -$C(OH)(\underline{C}H_3)_2$), 21.3 (-$CH_2\underline{C}H_2CH_3$), 13.8 (-$CH_2CH_2\underline{C}H_3$), 9.2 (medoxomil CH_3-5).

^{13}C-NMR (DMSO-d_6, 150 MHz) δ: 163.4 (tetrazole C-5), 160.6 (>C=O), 157.7 (imidazole C-4), 151.6 (medoxomil >C=O), 150.9 (imidazole C-2), 141.1 (biphenyl C-1′), 140.8 (3C, *ipso*-C of -CPh_3), 140.3 (medoxomil C-5 or C-4), 139.0 (biphenyl C-1), 136.0 (biphenyl C-4), 132.8 (medoxomil C-4 or C-5), 130.6 (biphenyl C-6′), 130.4 (biphenyl C-5'), 130.2 (biphenyl C-3′), 129.5 (6C, *ortho*-C of -CPh_3), 129.0 (2C, biphenyl C-2 and C-6), 128.2 (3C, *para*-C of -CPh_3), 127.8 (6C, *meta*-C of -CPh_3), 127.7 (biphenyl C-4'), 125.7 (biphenyl C-2′), 125.0 (2C, biphenyl C-3 and C-5), 116.2 (imidazole C-5), 82.2 (-$\underline{C}Ph_3$), 69.6 (-$\underline{C}(OH)(CH_3)_2$), 54.1 (-$CH_2O$-), 48.0 (>N-$\underline{C}H_2$-), 29.7 (2C, -$C(OH)(\underline{C}H_3)_2$), 28.2 (-$\underline{C}H_2CH_2CH_3$), 20.3 (-$CH_2\underline{C}H_2CH_3$), 13.5 (-$CH_2CH_2\underline{C}H_3$), 8.6 (medoxomil CH_3-5).

HRMS (ESI) *m/z* 801.3401 (calcd. for $C_{48}H_{45}N_6O_6$ $[M + H]^+$ 801.3387).

Crystal data for 6 acetone solvate: $C_{48}H_{44}N_6O_6 \cdot C_3H_6O$, M = 858.97, orthorhombic, $Pca2_1$, a = 13.493(3) Å, b = 11.100(3) Å, c = 29.124(4) Å, V = 4362.0(16) Å^3, Z = 4, D_c = 1.308 Mg·m^{-3}, T = 100(2) K, R = 0.037, wR = 0.077 [7234 reflections with $I > 2\sigma(I)$] for 577 variables. CCDC 1059381.

(5-Methyl-2-oxo-1,3-dioxolen-4-yl)methyl 4-(1-hydroxy-1-methylethyl)-2-propyl-1-[2'-(1*H*-tetrazol-5-yl)biphenyl-4-yl]methyl-1*H*-imidazole-5-carboxylate (7)

A solution of 96% H_2SO_4 (13.2 mL, 248.07 mmol, 3.0 eq) in H_2O (264 mL) was added to a suspension of the ester **6** (66.23 g, 82.69 mmol, 1.0 eq) in Me_2CO (132 mL). After heating at 50–55 °C for 2 h, TLC analysis (50% AcOEt/hexanes, R_f = 0.36 for **6**) indicated the disappearance of the starting material. The hot solution was diluted with H_2O (264 mL) and then cooled to 10 °C in an ice-water bath. Precipitated triphenylmethanol was removed by filtration and washed with H_2O (4 × 25 mL). The combined filtrate and washings were diluted with CH_2Cl_2 (200 mL) and then, while stirring,

very carefully neutralized with Na_2CO_3 (26.73 g, 252.20 mmol, 3.05 eq) solution in H_2O (100 mL). The resulting layers were separated and the aqueous layer was extracted with CH_2Cl_2 (2 × 50 mL). The combined organic phases were washed with brine (500 mL), dried over anhydrous $MgSO_4$ (30 g), filtered and concentrated by evaporation under reduced pressure to give a light-yellow solid (HPLC purity: **9** (0.64%), **10** (0.51%), **7** (98.63%) and the sum of other impurities (0.22%)). The crude medoxomil ester **7** was dissolved in Me_2CO (700 mL) under reflux. The resulting solution was filtered and the excess of Me_2CO (520 mL) was distilled off. AcOEt (230 mL) was added to the residue while stirring and 230 mL of a mixture of solvents were then distilled off. The resulting mixture was allowed to cool to room temperature. The precipitated solid was filtered off, washed with AcOEt (50 mL), dried in air and then in vacuum at room temperature to afford olmesartan medoxomil (**7**, 36.76 g, 80% yield). HPLC purity: **9** (0.04%), **10** (0.08%), **7** (99.20%) and the sum of other impurities (0.68%).

M.p. 182.00–186.14 °C, peak 183.07 °C, heating rate 10.00 °C/min (white crystals). Lit. m.p.: 182–184 °C (isobutanol) [20,21], 182–184 °C (THF) [20,21,26], 180–182 °C (ethanol, dec.) [19], 180–182 °C (acetone) [25], 177–180 °C (ethyl acetate, dec.) [18], 175–177 °C (t-butyl-methyl ether-ethyl acetate or ethyl methyl ketone or methanol-water) [28], 170–172 °C [18], 120–140 °C (heptane) [20,21].

FT-IR (KBr) ν: 3396, 3290, 3040, 2972, 2931, 1832, 1740, 1708, 1502, 1474, 1401, 1389, 1302, 1226, 1169, 1136, 1054, 1003, 953, 782, 761 cm^{-1}. Lit. IR (KBr) ν: 1831, 1707, 1391, 1300, 1167, 1140, 1003, 768, 765 cm^{-1} [26]; 3398, 3291, 3040, 3004, 2972, 2931, 2874, 1832, 1708, 1474, 1389, 1169, 1136, 1053, 782, 761 cm^{-1} [27]; 3291, 1833, 1740, 1708 cm^{-1} [19]; 1832, 1740, 1707 cm^{-1} [25].

^{1}H-NMR ($CDCl_3$, 600 MHz) δ: 7.82 (1H, dd, *J* = 7.5 and 1.2 Hz, biphenyl H-3′), 7.60 (1H, m, biphenyl H-5′), 7.52 (1H, m, biphenyl H-4′), 7.44 (1H, dd, *J* = 7.2 and 1.2 Hz, biphenyl H-6′), 7.08 (2H, BB′ of AA′BB′ system, biphenyl H-2 and H-6), 6.79 (2H, AA′ of AA′BB′ system, biphenyl H-3 and H-5), 5.79 (1H, br. s, -OH), 5.41 (2H, s, >N-CH_2-), 4.96 (2H, s, -CH_2O-), 2.54 (2H, t, *J* = 7.2 Hz, -$C\underline{H}_2CH_2CH_3$), 2.18 (3H, s, medoxomil CH_3-5), 1.67 (2H, m, -$CH_2C\underline{H}_2CH_3$), 1.59 (6H, s, -C(OH)($C\underline{H}_3$)$_2$), 0.91 (3H, t, *J* = 7.2 Hz, -$CH_2CH_2C\underline{H}_3$).

Lit. ^{1}H-NMR ($CDCl_3$) δ: 7.81 (1H, dd), 7.43-7.6 (3H, m), 7.09 (2H, d), 6.79 (2H, d), 5.41 (1H, s), 4.95 (1H, s), 2.56 (3H, t), 2.17 (3H, s), 1.58–1.69 (2H, m), 1.58 (6H, s), 0.92 (3H, t) [24]; 7.83 (1H, dd, *J* = 1.0 and 7.5 Hz), 7.42–7.63 (3H, m), 7.10 (2H, d, *J* = 8 Hz), 6.83 (2H, d, *J* = 8.0 Hz), 5.45 (2H, s), 5.00 (2H, s), 2.70 (2H, t, *J* = 7.5 Hz), 2.19 (3H, s), 1.6–1.8 (2H, m), 1.63 (6H, s), 0.93 (3H, t, *J* = 7.5 Hz) [18]; (260 MHz) 7.72 (1H, dd, *J* = 1.7 Hz), 7.3–7.5 (3H, m), 6.99 (2H, d, *J* = 8,0 Hz), 6.70 (2H, d, *J* = 8.0 Hz), 5.32 (2H, s), 4.86 (2H, s), 2.48 (2H, t, *J* = 7.5 Hz), 2.07 (3H, s), 1.54–1.63 (2H, m), 1.50 (6H, s), 0.82 (3H, t, *J* = 7.5 Hz) [28].

^{1}H-NMR (DMSO-d_6, 600 MHz) δ: 16.30 (1H, br. s, >N-H), 7.68 (1H, dd, *J* = 7.5 and 1.2 Hz, biphenyl H-5′), 7.65 (1H, m, biphenyl H-3′), 7.57 (1H, m, biphenyl H-4′), 7.54 (1H, dd, *J* = 7.2 and 1.2 Hz, biphenyl H-6′), 7.05 (2H, BB′ of AA′BB′ system, biphenyl H-2 and H-6), 6.87 (2H, AA′ of AA′BB′ system, biphenyl H-3 and H-5), 5.43 (2H, s, >N-CH_2-), 5.22 (1H, br. s, -OH), 5.06 (2H, s, -CH_2O-), 2.61 (2H, t, *J* = 7.2 Hz, -$C\underline{H}_2CH_2CH_3$), 2.08 (3H, s, medoxomil CH_3-5), 1.59 (2H, m, -$CH_2C\underline{H}_2CH_3$), 1.48 (6H, s, -C(OH)($C\underline{H}_3$)$_2$), 0.88 (3H, t, *J* = 7.2 Hz, -$CH_2CH_2C\underline{H}_3$).

Lit. ^{1}H-NMR (DMSO-d_6) δ: (500 MHz) 7.68 (1H, dt, *J* = 1.5 and 8.5 Hz), 7.65 (1H, dd, *J* = 1.5 and 8.5 Hz), 7.57 (1H, dt, *J* = 1.5 and 8.5 Hz), 7.54 (1H, d, *J* = 8.0 Hz), 7.05 (2H, d, *J* = 8.5 Hz), 6.87 (2H, d, *J* = 8.5 Hz), 5.43 (2H, s), 5.22 (1H, s), 5.06 (2H, s), 2.61 (2H, t, *J* = 8.0 Hz), 2.08 (3H, s), 1.58 (2H, m), 1.48 (6H, s), 0.88 (3H, t, *J* = 7.5 Hz) [27]; 7.52–7.70 (4H, m), 7.04 (2H, d, *J* = 8.5 Hz), 6.86 (2H, d, *J* = 8.5 Hz), 5.42 (2H, s), 5.20 (1H, s), 5.05 (2H, s), 2.60 (2H, t, *J* = 7.5 Hz), 2.08 (3H, s), 1.58 (2H, m, *J* = 7.5 Hz), 1.47 (6H, s), 0.88 (3H, t, *J* = 7.5 Hz) [19]; (300 MHz) 7.50–7.69 (4H, m), 7.03 (2H, d, *J* = 8.0 Hz), 6.85 (2H, d, *J* = 8.0 Hz), 5.41 (2H, s), 5.22 (1H, s), 5.05 (2H, s), 2.50 (2H, s), 2.08 (3H, s) [29].

^{13}C-NMR ($CDCl_3$, 150 MHz) δ: 161.0 (imidazole C-4), 160.8 (>C=O), 155.1 (tetrazole C-5), 152.8 (medoxomil >C=O), 152.5 (imidazole C-2), 140.9 (medoxomil C-4 or C-5), 140.6 (biphenyl C-1′), 138.7 (biphenyl C-1), 136.4 (biphenyl C-4), 133.2 (medoxomil C-4 or C-5), 131.3 (biphenyl C-5'), 130.9 (biphenyl C-3′), 130.7 (biphenyl C-6′), 129.5 (2C, biphenyl C-2 and C-6), 128.2 (biphenyl C-4′), 125.2 (2C, biphenyl C-3 and C-5), 122.8 (biphenyl C-2′), 116.2 (imidazole C-5), 70.7 ($-\underline{C}(OH)(CH_3)_2$), 53.9 ($-\underline{C}H_2O-$), 49.1 ($>N-\underline{C}H_2-$), 29.1 ($-\underline{C}H_2CH_2CH_3$), 29.0 (2C, $-C(OH)(\underline{C}H_3)_2$), 21.2 ($-CH_2\underline{C}H_2CH_3$), 13.8 ($-CH_2CH_2\underline{C}H_3$), 9.4 (medoxomil CH_3-5).

^{13}C-NMR (DMSO-d_6, 150 MHz) δ: 160.7 (>C=O), 157.5 (imidazole C-4), 155.0 (tetrazole C-5), 151.7 (medoxomil >C=O), 151.0 (imidazole C-2), 141.0 (biphenyl C-1′), 140.4 (medoxomil C-5 or C-4), 138.1 (biphenyl C-1), 136.6 (biphenyl C-4), 132.8 (medoxomil C-4 or C-5), 131.0 (biphenyl C-5′), 130.5 (biphenyl C-3′), 130.5 (biphenyl C-6′), 129.0 (2C, biphenyl C-2 and C-6), 127.8 (biphenyl C-4′), 125.4 (2C, biphenyl C-3 and C-5), 123.5 (biphenyl C-2′), 116.2 (imidazole C-5), 69.6 ($-\underline{C}(OH)(CH_3)_2$), 54.1 ($-\underline{C}H_2O-$), 48.0 ($>N-\underline{C}H_2-$), 29.7 (2C, $-C(OH)(\underline{C}H_3)_2$), 28.2 ($-\underline{C}H_2CH_2CH_3$), 20.6 ($-CH_2\underline{C}H_2CH_3$), 13.6 ($-CH_2CH_2\underline{C}H_3$), 8.7 (medoxomil CH_3-5).

Lit. ^{13}C-NMR (DMSO-d_6) δ: 160.7, 157.7, 155.1, 151.7, 151.1, 141.1, 140.4, 138.2, 136.6, 132.8, 130.9, 130.5, 129.0, 127.7, 125.4, 123.6, 116.2, 69.7, 54.1, 48.1, 29.6, 28.3, 20.6, 13.5, 8.7 [27].

HRMS (ESI) *m/z* 559.2306 (calcd. for $C_{29}H_{31}N_6O_6$ $[M + H]^+$ 559.2305).

4-(1-Hydroxy-1-methylethyl)-2-propyl-1-[2′-(1H-tetrazol-5-yl)biphenyl-4-yl]-methyl-1H-imidazole-5-carboxylic acid (olmesartan, 8)

A solution of NaOH (0.716 g, 17.9 mmol, 2.0 eq) in H_2O (15 mL) was added to a solution of olmesartan medoxomil (**7**, 5.0 g, 8.95 mmol, 1.0 eq) in MeOH (100 mL). After being stirred at room temperature for 24 h, TLC analysis (MeOH/AcOEt 30%) indicated the disappearance of the starting material **7**. MeOH was evaporated under reduced pressure and the residue was portioned between H_2O (100 mL) and AcOEt (25 mL). The aqueous layer was separated and acidified to pH 5.5–6.0 by the dropwise addition of glacial AcOH. The resulting mixture was stirred at room temperature for additional 30 min. The precipitated solid was filtered off, washed with H_2O and dried under vacuum at room temperature. The crude product (3.98 g) was purified by maceration in Me_2CO to afford olmesartan (**8**, 3.83 g, 96%).

M.p. 172.90–185.87 °C, peak 179.46 °C, heating rate 10.00 °C/min (white powder). Lit. m.p.: 166–169 °C (diisopropyl ether) [18], 199–201 °C (EtOH) [19].

FT-IR (KBr) ν: 3432, 2972, 1637, 1572, 1509, 1463, 1432, 1364, 1336, 1193, 979, 874, 825, 761 cm^{-1}. Lit. IR (KBr) ν: 3429, 3066, 1637 cm^{-1} [30].

^{1}H-NMR (DMSO-d_6, 600 MHz) δ: 7.67 (1H, dd, *J* = 7.5 and 1.2 Hz, biphenyl H-5′), 7.64 (1H, m, biphenyl H-3′), 7.56 (1H, m, biphenyl H-4′), 7.53 (1H, dd, *J* = 7.2 and 1.2 Hz, biphenyl H-6′), 7.06 (2H, BB′ of AA′BB′ system, biphenyl H-2 and H-6), 6.95 (2H, AA′ of AA′BB′ system, biphenyl H-3 and H-5), 5.65 (2H, s, $>N-CH_2-$), 2.58 (2H, t, *J* = 7.2 Hz, $-C\underline{H}_2CH_2CH_3$), 1.54 (6H, s, $-C(OH)(C\underline{H}_3)_2$), 1.53 (2H, m, $-CH_2C\underline{H}_2CH_3$), 0.85 (3H, t, *J* = 7.2 Hz, $-CH_2CH_2C\underline{H}_3$).

Lit. ^{1}H-NMR (DMSO-d_6) δ: (400 MHz) 7.62–7.70 (2H, m), 7.51-7.59 (2H, m), 7.06 (2H, d, *J* = 8.3 Hz), 6.94 (2H, d, *J* = 8.3 Hz), 5.64 (2H, s), 2.57 (2H, t, *J* = 7.6 Hz), 1.53 (6H, s), 1.53 (2H, tq, J= 7.3 and 7.6 Hz), 0.85 (3H, t, *J* = 7.3 Hz) [31]; 7.5–7.7 (4H, m), 7.06 (2H, d, *J* = 8.5 Hz), 6.94 (2H, d, *J* = 8.5 Hz), 5.64 (2H, s), 2.58 (2H, t, *J* = 8 Hz), 1.4-1.6 (2H, m), 1.54 (6H, s), 0.85 (3H, t, *J* = 7.5 Hz), [18]; 7.59–7.61 (2H, m), 7.47–7.53 (2H, m), 7.01–7.03 (2H, m), 6.89–6.91 (2H, m), 5.61 (2H, s), 2.54 (2H, t), 1.49–1.51 (8H, m), 0.82 (3H, t) [32]; (400 MHz) 7.5–7.8 (4H, m), 6.9–7.2 (4H, m), 5.7 (2H, s), 2.6 (2H, t, *J* = 5.4 Hz), 1.4–1.7 (2H, m), 1.6 (6H, s), 0.8 (3H, t, *J* = 5.6 Hz) [30].

^{13}C-NMR (DMSO-d_6, 150 MHz) δ: 160.9 (>C=O), 155.0 (tetrazole C-5), 153.1 (imidazole C-4), 150.7 (imidazole C-2), 141.0 (biphenyl C-1′), 138.1 (biphenyl C-1), 136.8 (biphenyl C-4), 131.1 (biphenyl C-5′), 130.6 (biphenyl C-3′), 130.6 (biphenyl C-6′), 129.0 (2C, biphenyl C-2 and C-6), 127.8 (biphenyl C-4'), 126.0 (2C, biphenyl C-3 and C-5), 123.4 (biphenyl C-2'), 118.0 (imidazole C-5), 70.8 (-$\underline{C}(OH)(CH_3)_2$), 47.0 (>N-$\underline{C}H_2$-), 29.7 (2C, -$C(OH)(\underline{C}H_3)_2$), 28.0 (-$\underline{C}H_2CH_2CH_3$), 20.4 (-$CH_2\underline{C}H_2CH_3$), 13.6 (-$CH_2CH_2\underline{C}H_3$).

HRMS (ESI) *m/z* 447.2134 (calcd. for $C_{24}H_{27}N_6O_3$ $[M + H]^+$ 447.2145).

Spectral data (NMR, IR, HRMS) and DSC thermograms of the bromide 2 and compounds synthesized

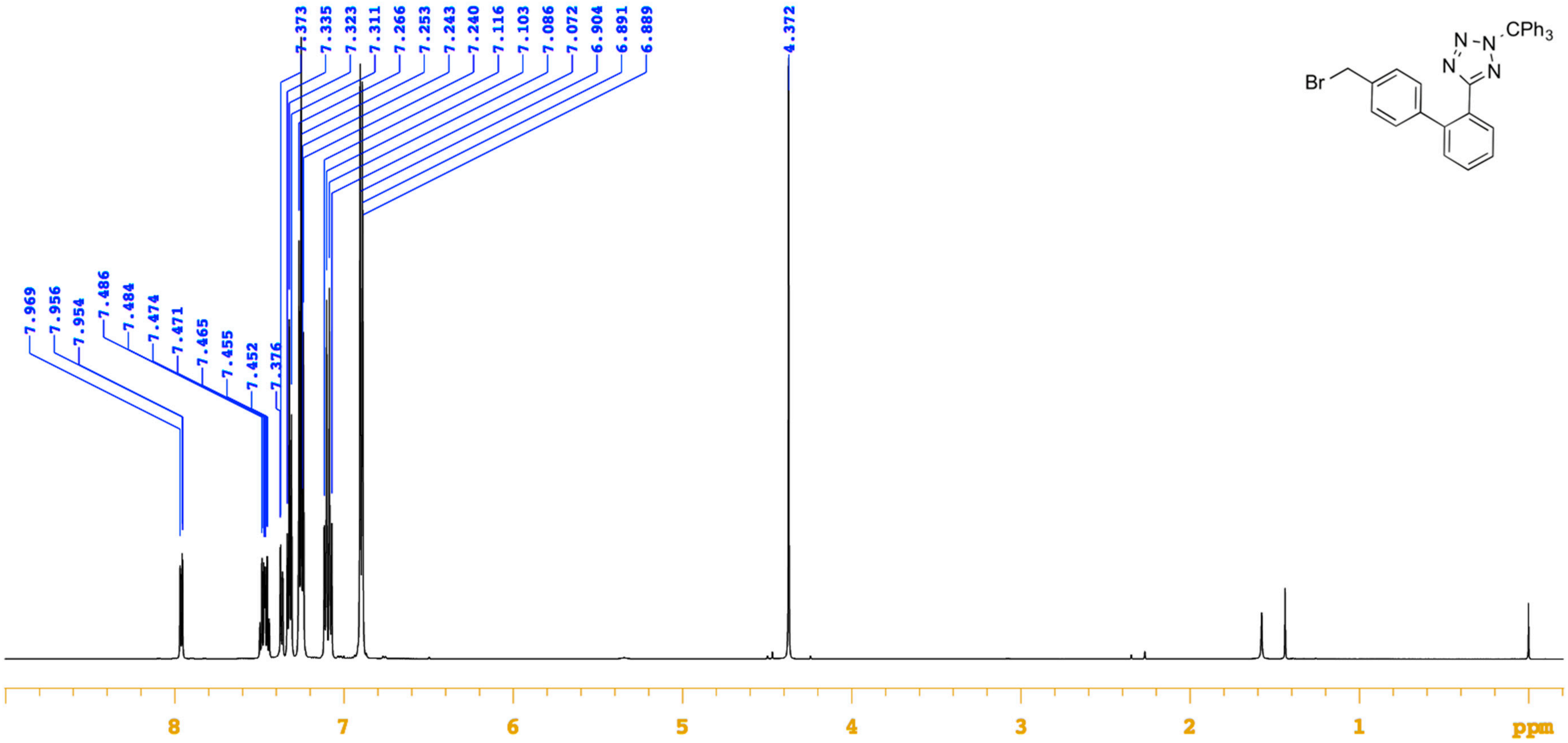

Figure S1. ^{1}H-NMR (600 MHz, $CDCl_3$, δ, ppm) spectrum of the bromide **2**.

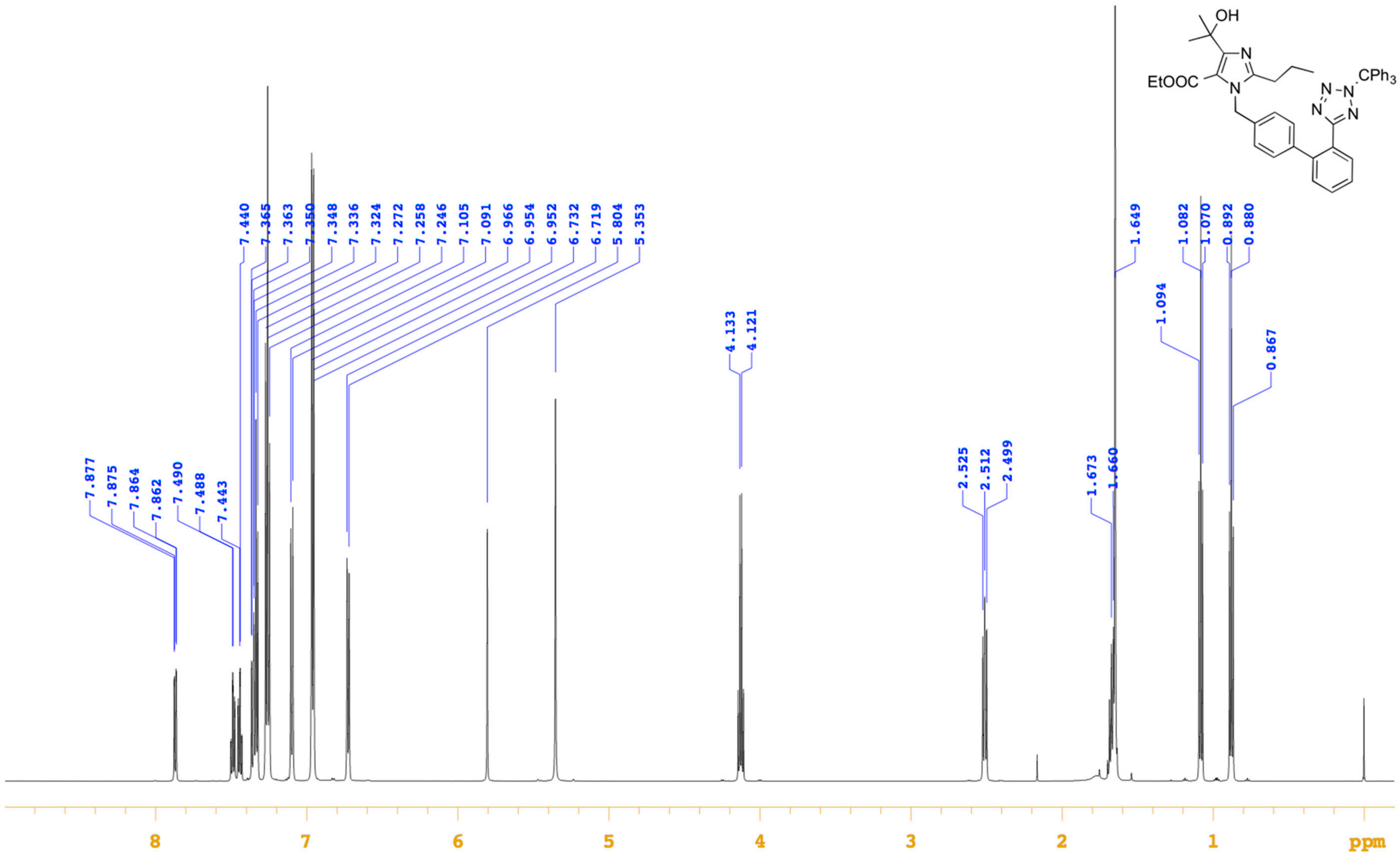

Figure S2. ¹H-NMR (600 MHz, $CDCl_3$, δ, ppm) spectrum of the ethyl ester **3**.

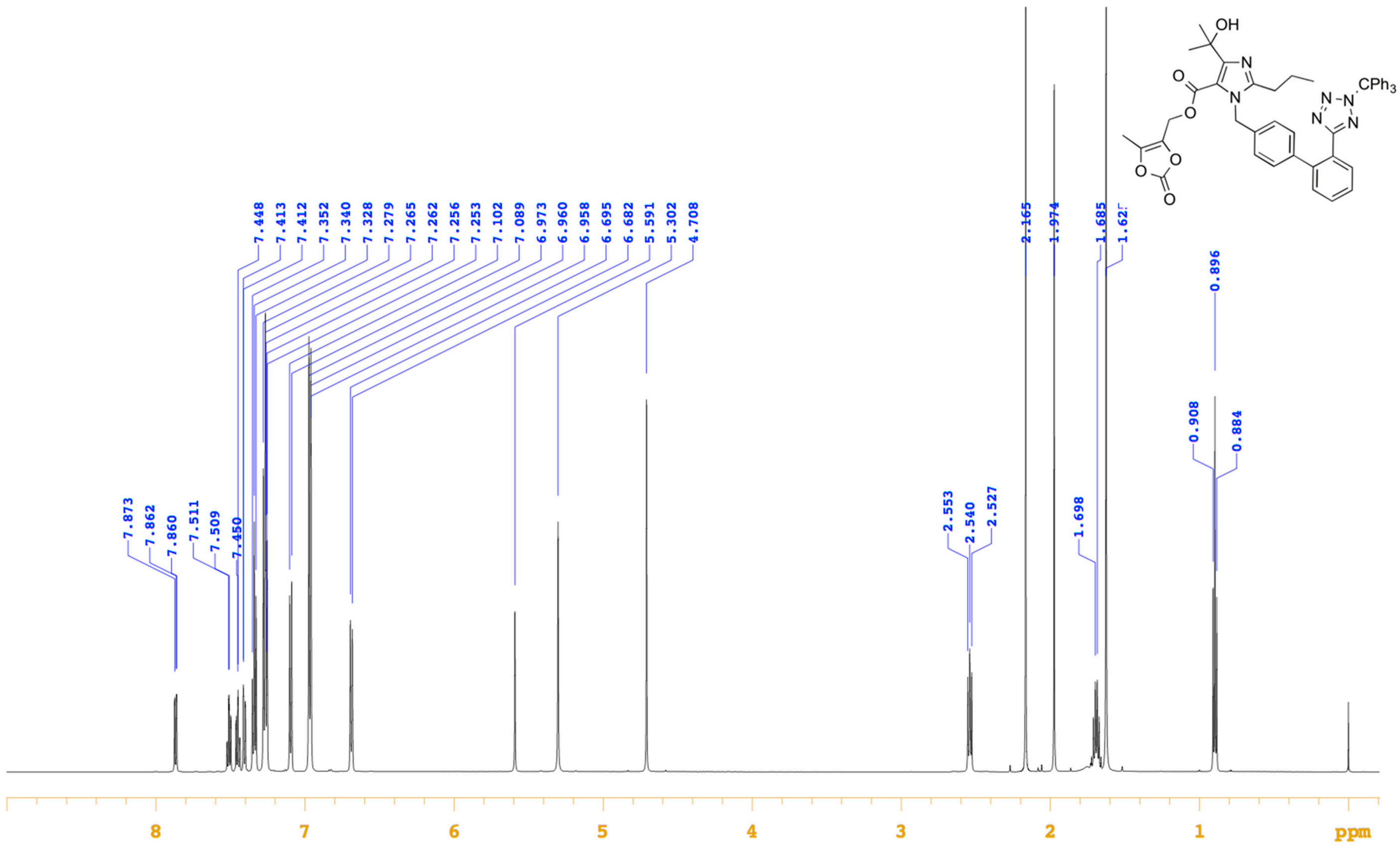

Figure S3. ^{1}H-NMR (600 MHz, $CDCl_3$, δ, ppm) spectrum of the medoxomil ester **6**.

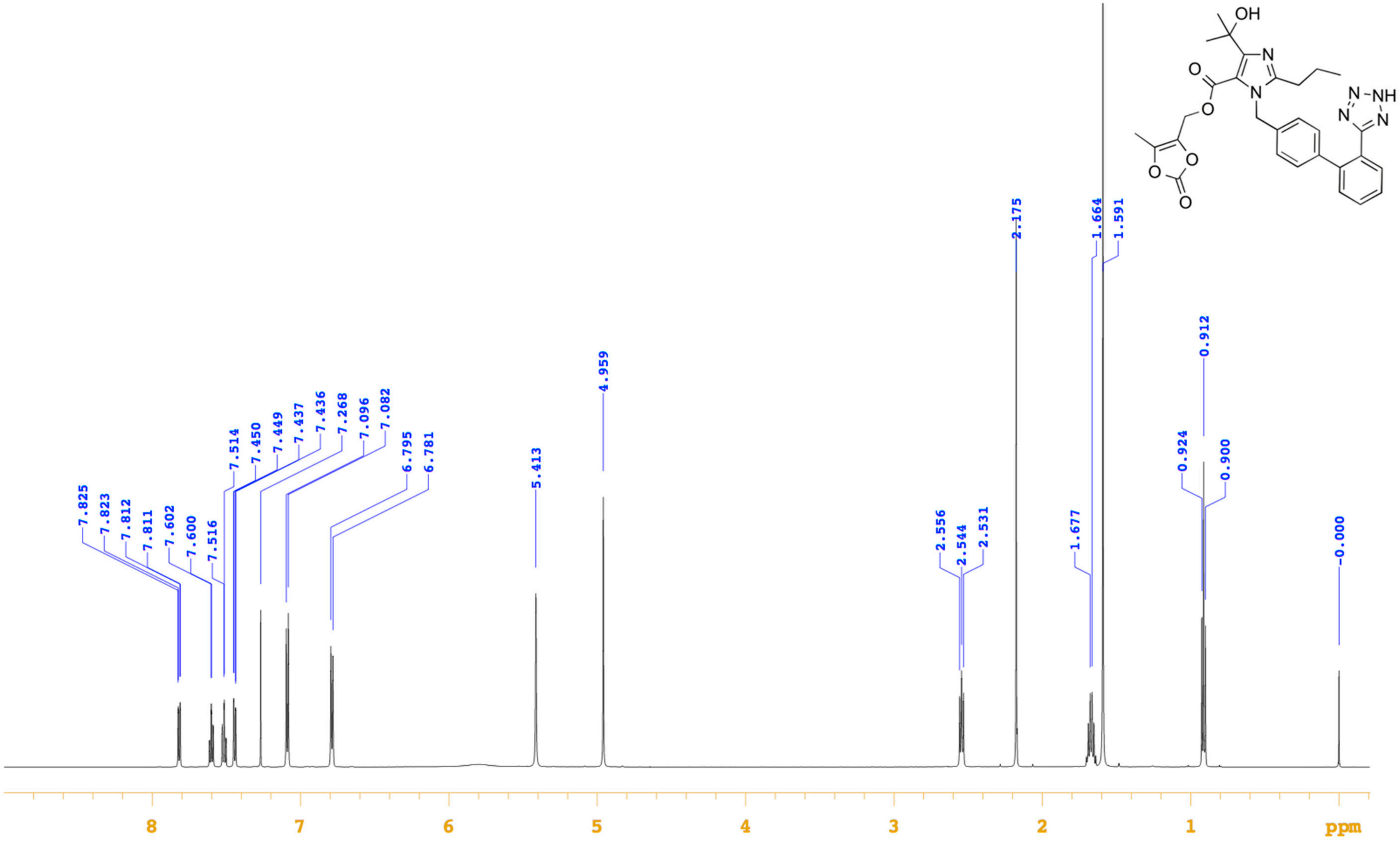

Figure S4. ^{1}H-NMR (600 MHz, $CDCl_3$, δ, ppm) spectrum of the olmesartan medoxomil (**7**).

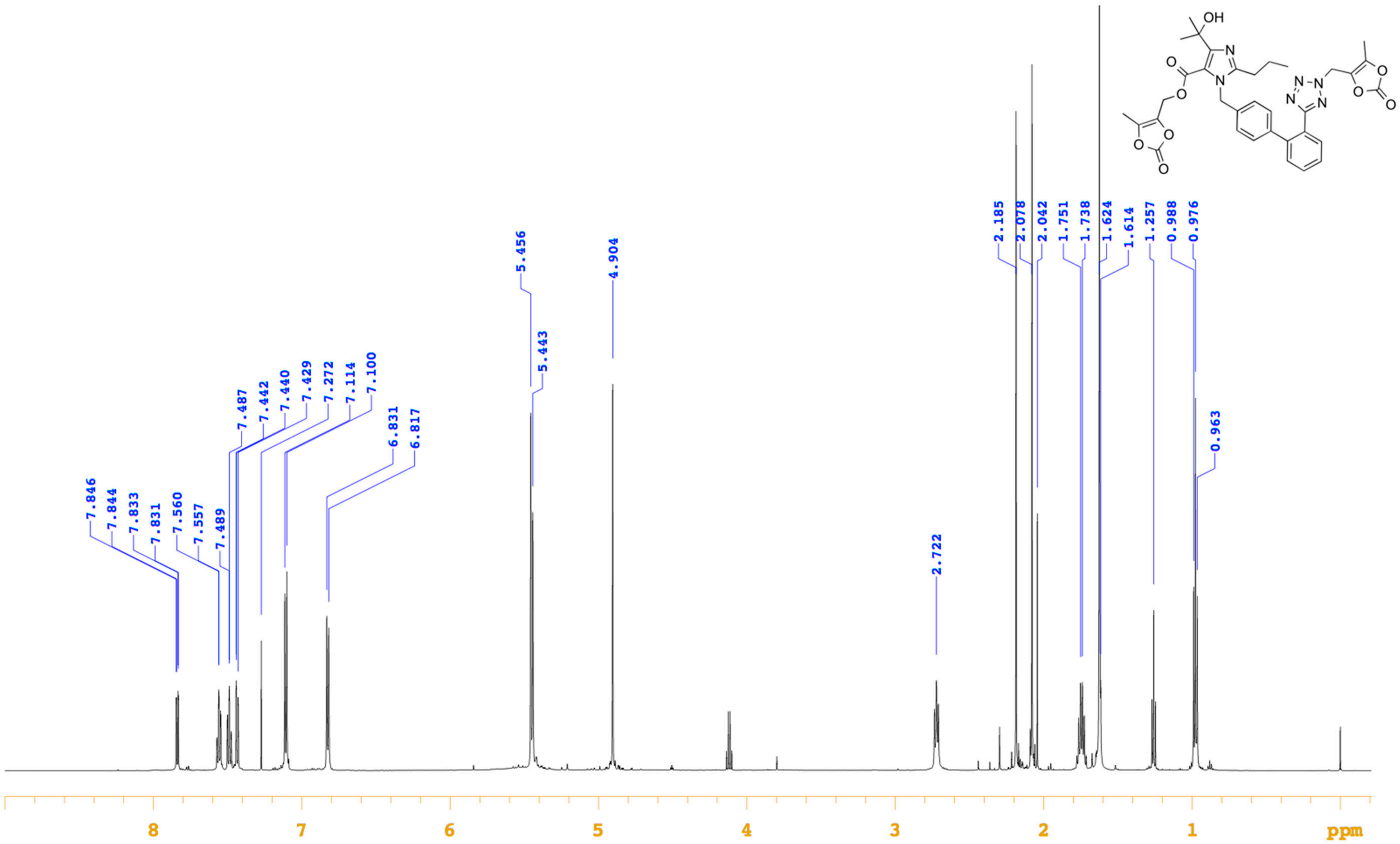

Figure S5. ^{1}H-NMR (600 MHz, $CDCl_3$, δ, ppm) spectrum of the *N*-2 substituted medoxomil impurity **9**.

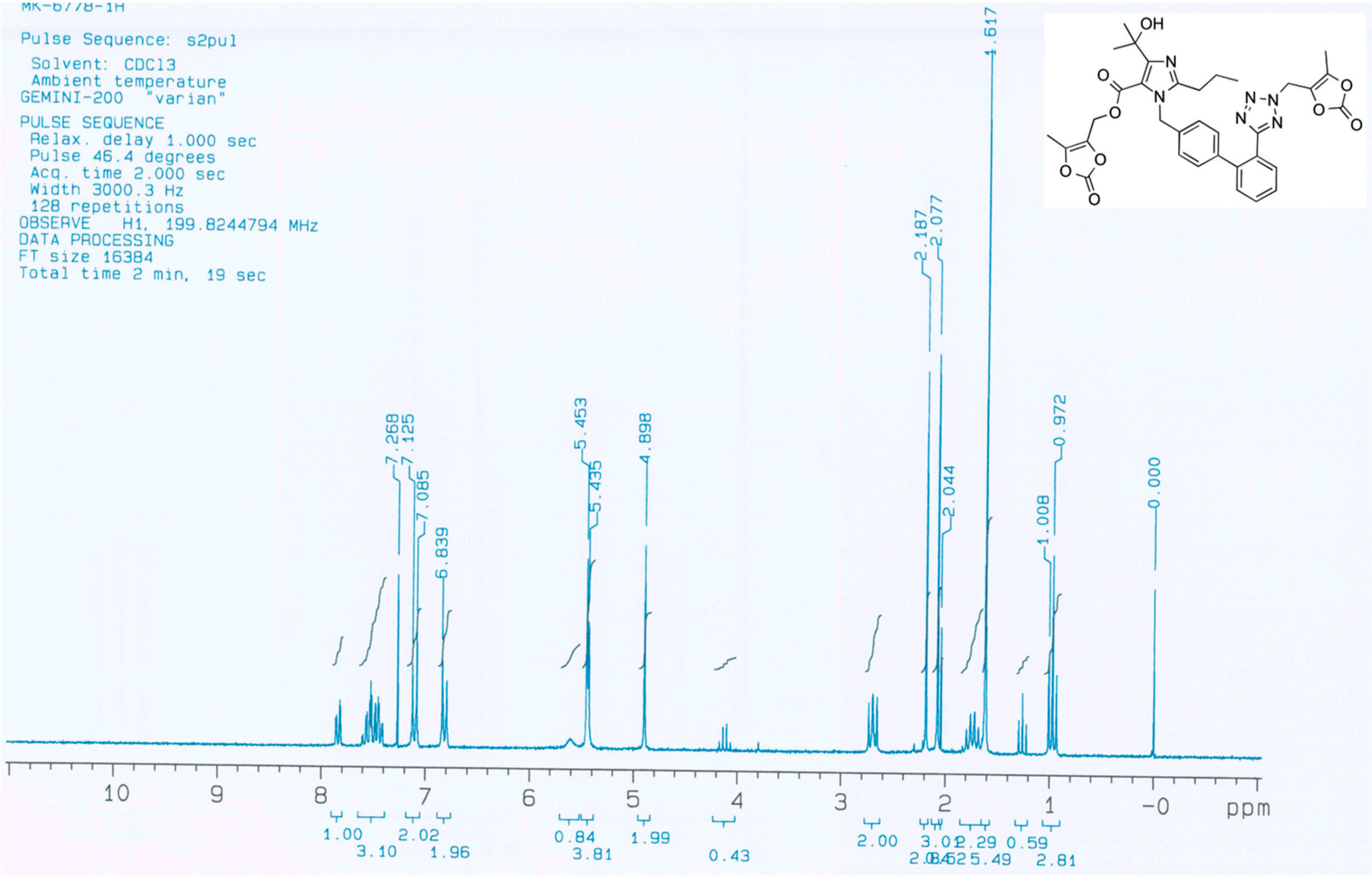

Figure S6. ^{1}H-NMR (200 MHz, $CDCl_3$, δ, ppm) spectrum of the *N*-2 substituted medoxomil impurity **9**.

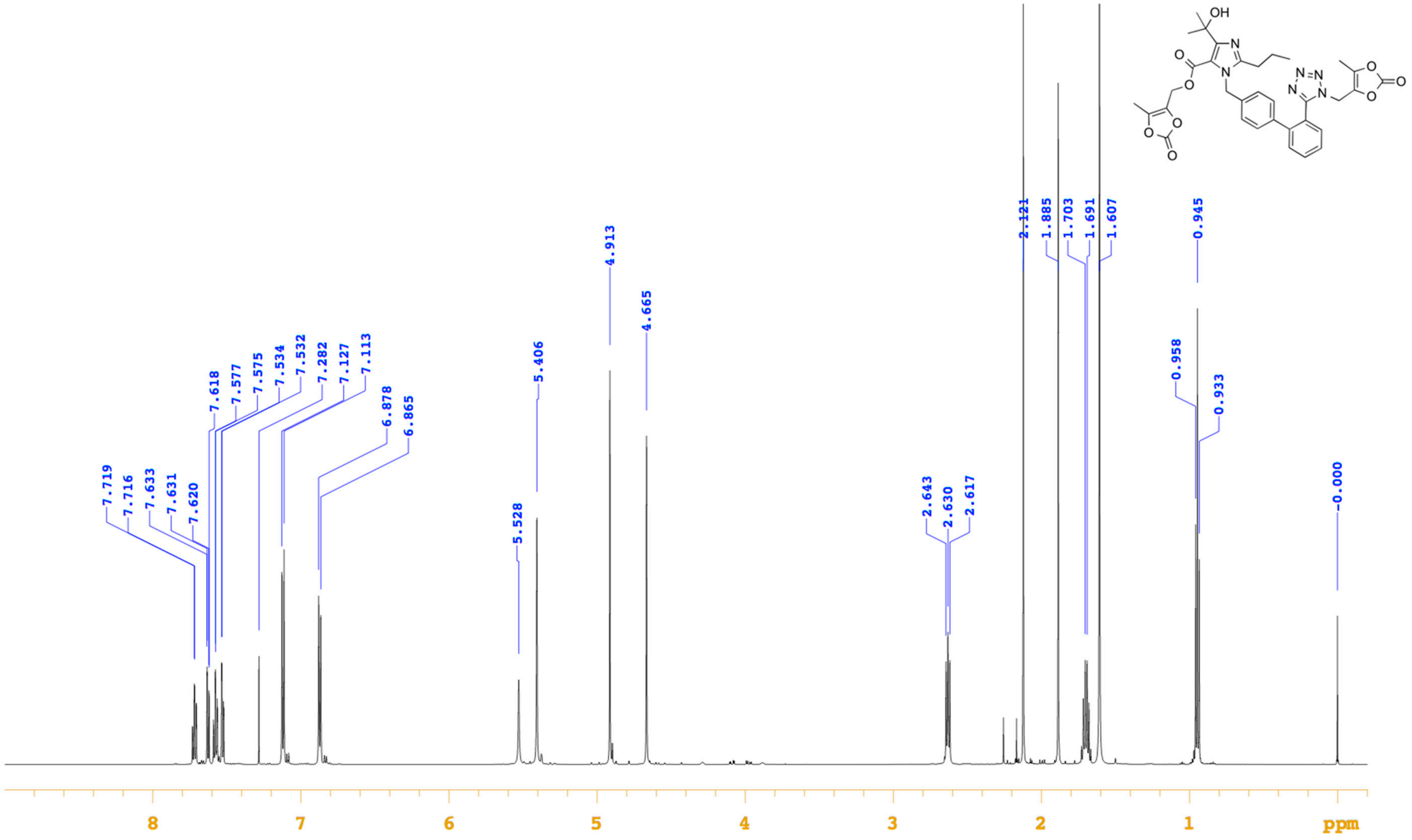

Figure S7. ^{1}H-NMR (600 MHz, CDCl3, δ, ppm) spectrum of the *N*-1 substituted medoxomil impurity **10**.

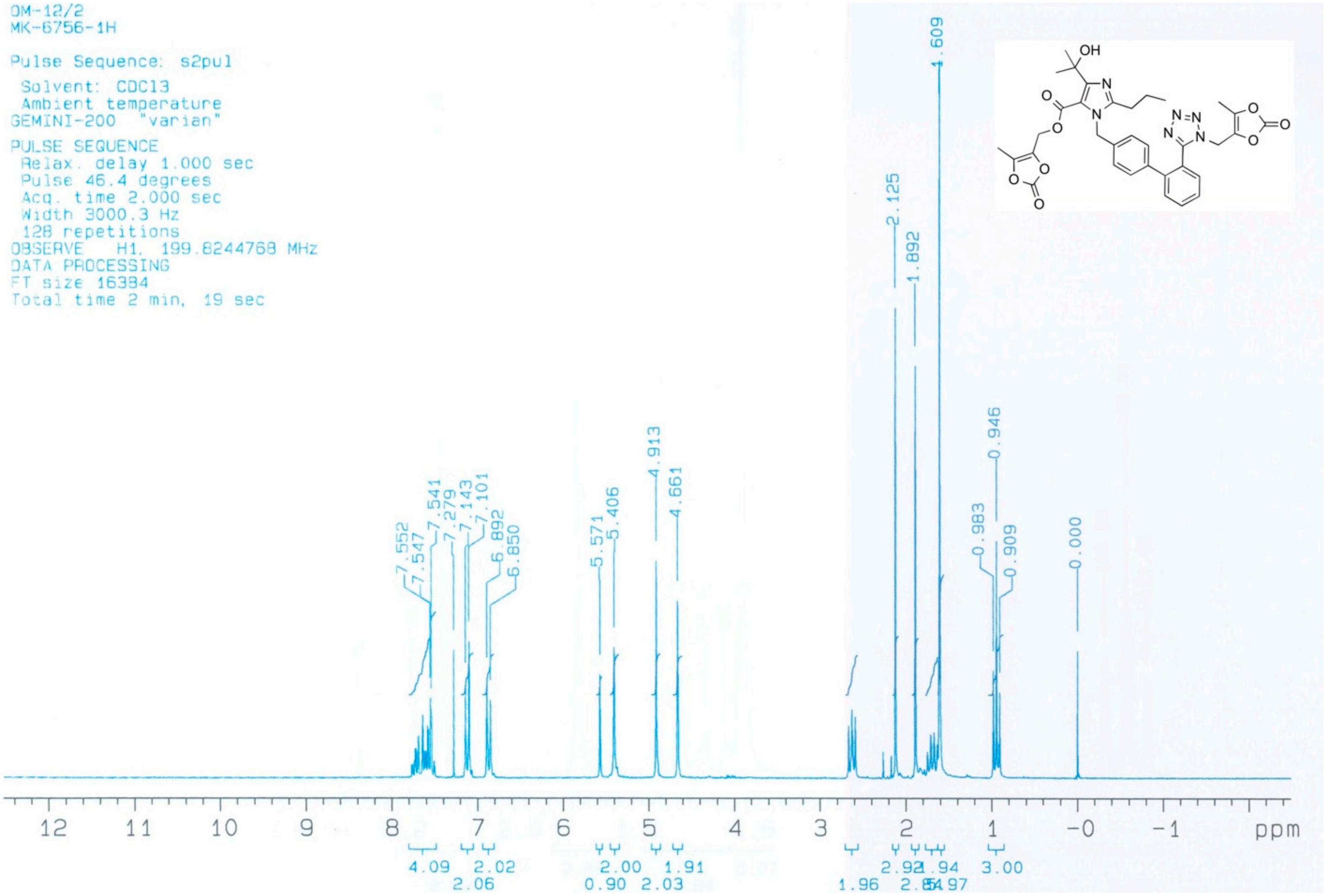

Figure S8. ^{1}H-NMR (200 MHz, $CDCl_3$, δ, ppm) spectrum of the *N*-1 substituted medoxomil impurity **10**.

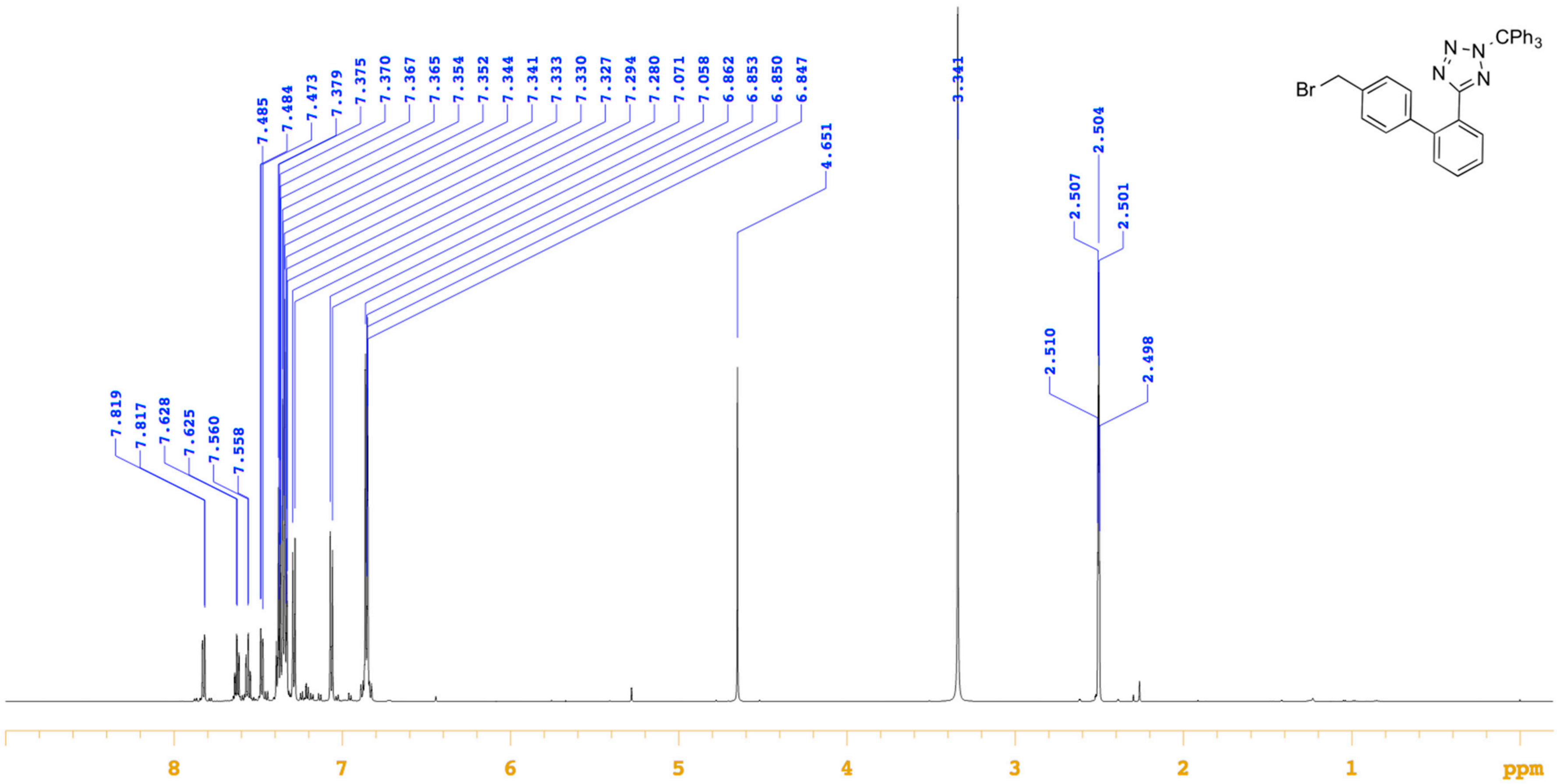

Figure S9. ^{1}H-NMR (600 MHz, DMSO-d_6, δ, ppm) spectrum of the bromide **2.**

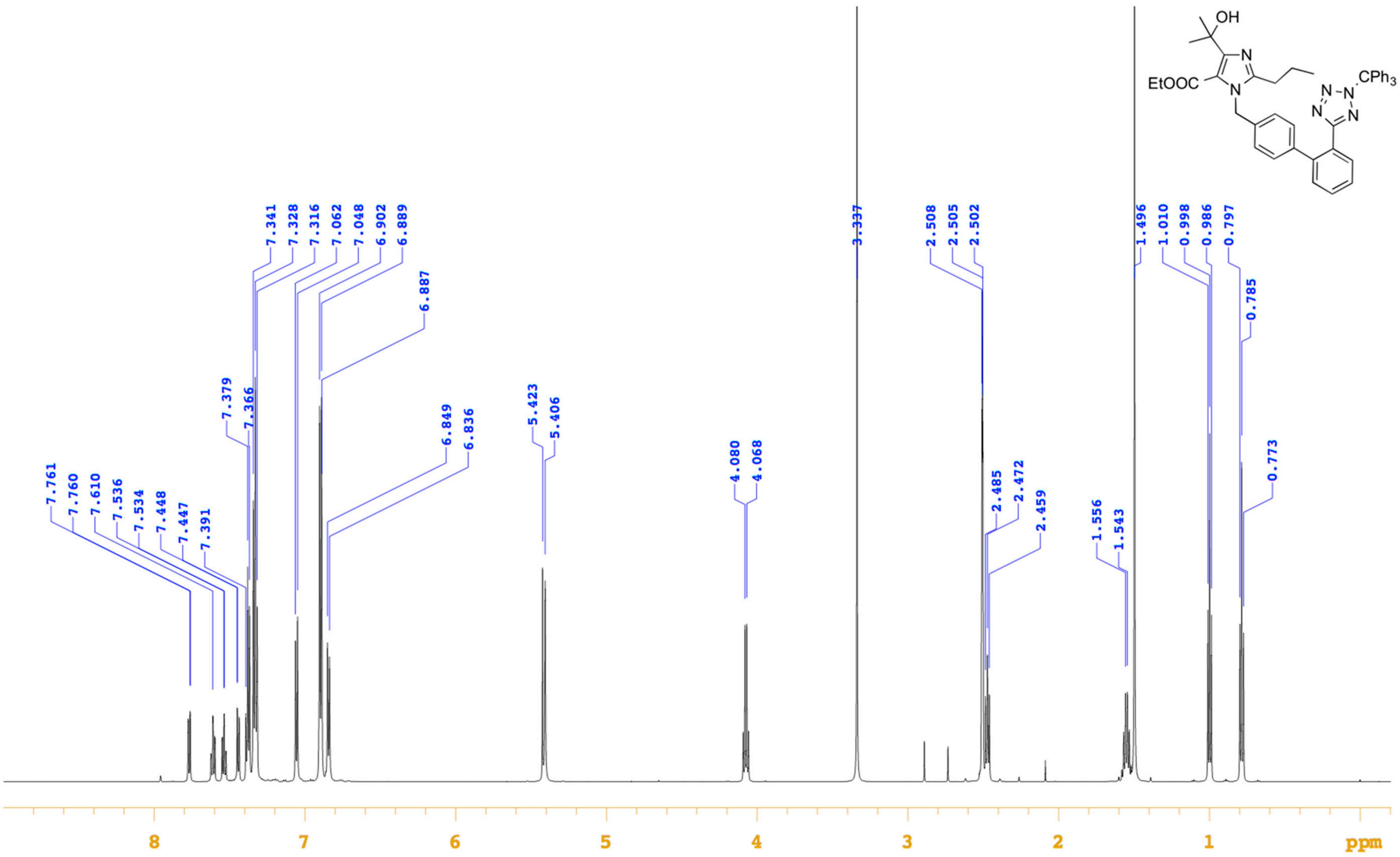

Figure S10. ^{1}H-NMR (600 MHz, DMSO-d_6, δ, ppm) spectrum of the ethyl ester **3**.

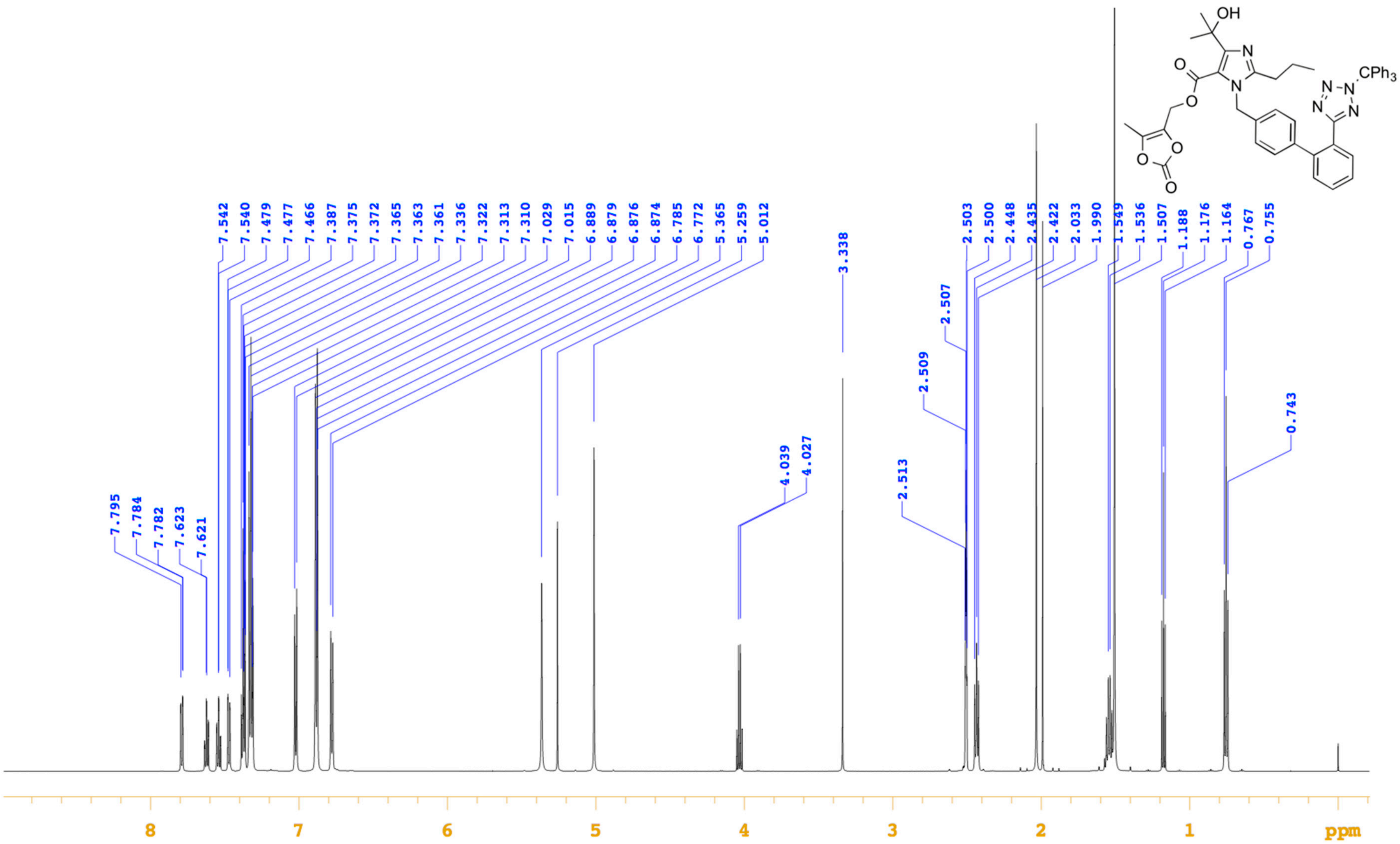

Figure S11. ^{1}H-NMR (600 MHz, DMSO-d_6, δ, ppm) spectrum of the medoxomil ester **6**.

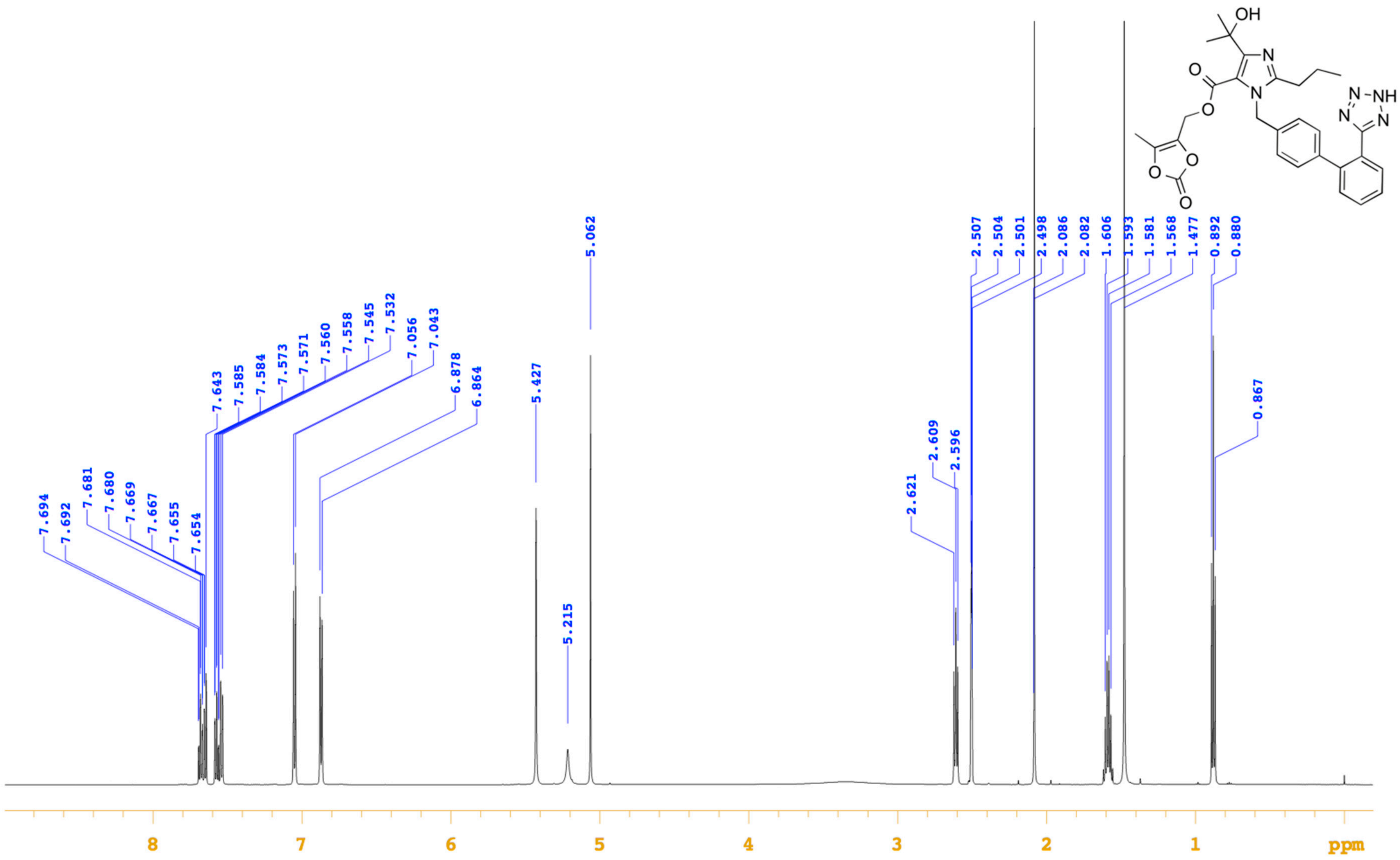

Figure S12. ^{1}H-NMR (600 MHz, DMSO-d_6, δ, ppm) spectrum of the olmesartan medoxomil (**7**).

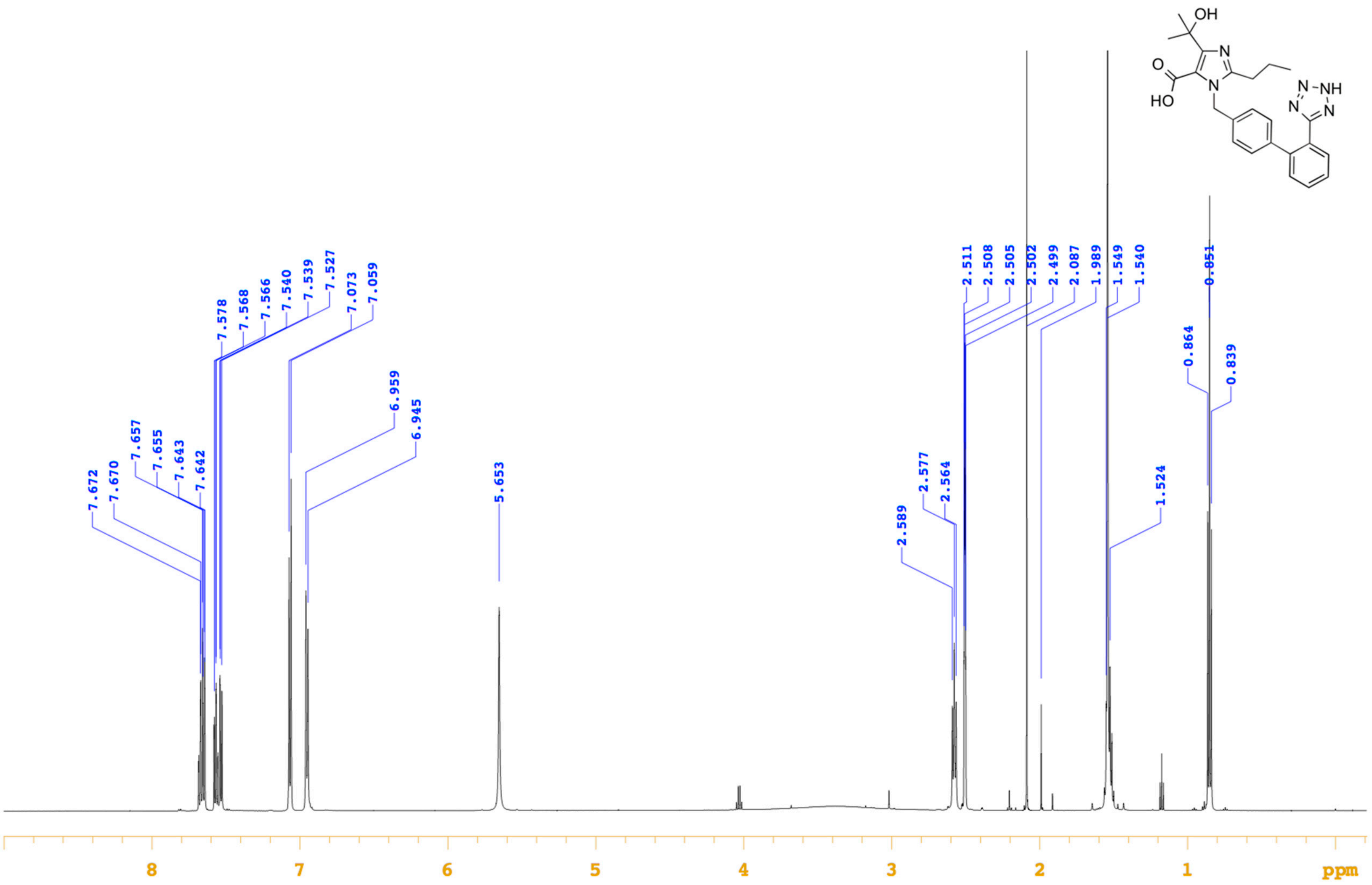

Figure S13. ^{1}H-NMR (600 MHz, DMSO-d_6, δ, ppm) spectrum of the olmesartan (**8**).

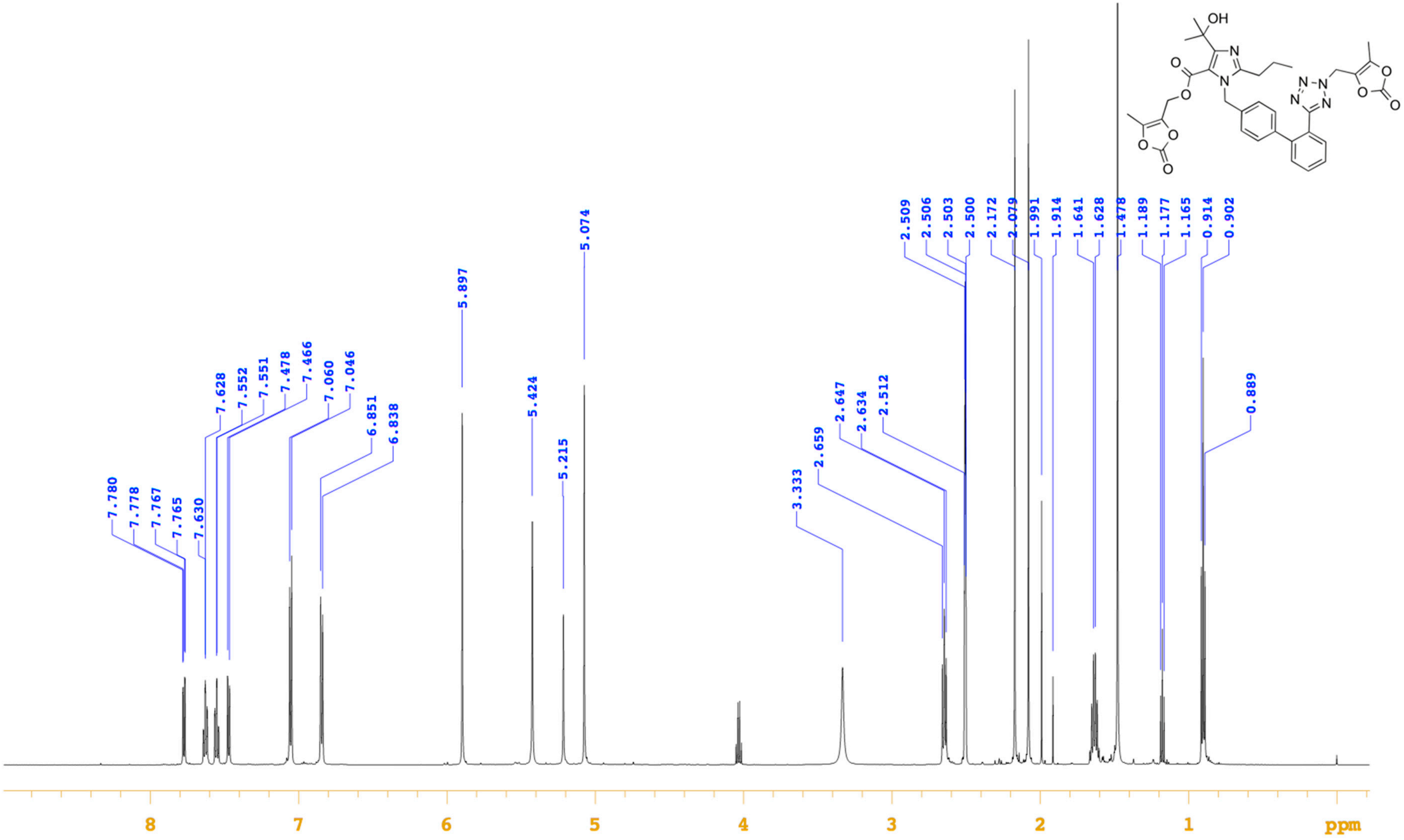

Figure S14. ^{1}H-NMR (600 MHz, DMSO-d_6, δ, ppm) spectrum of the *N*-2 substituted medoxomil impurity **9**.

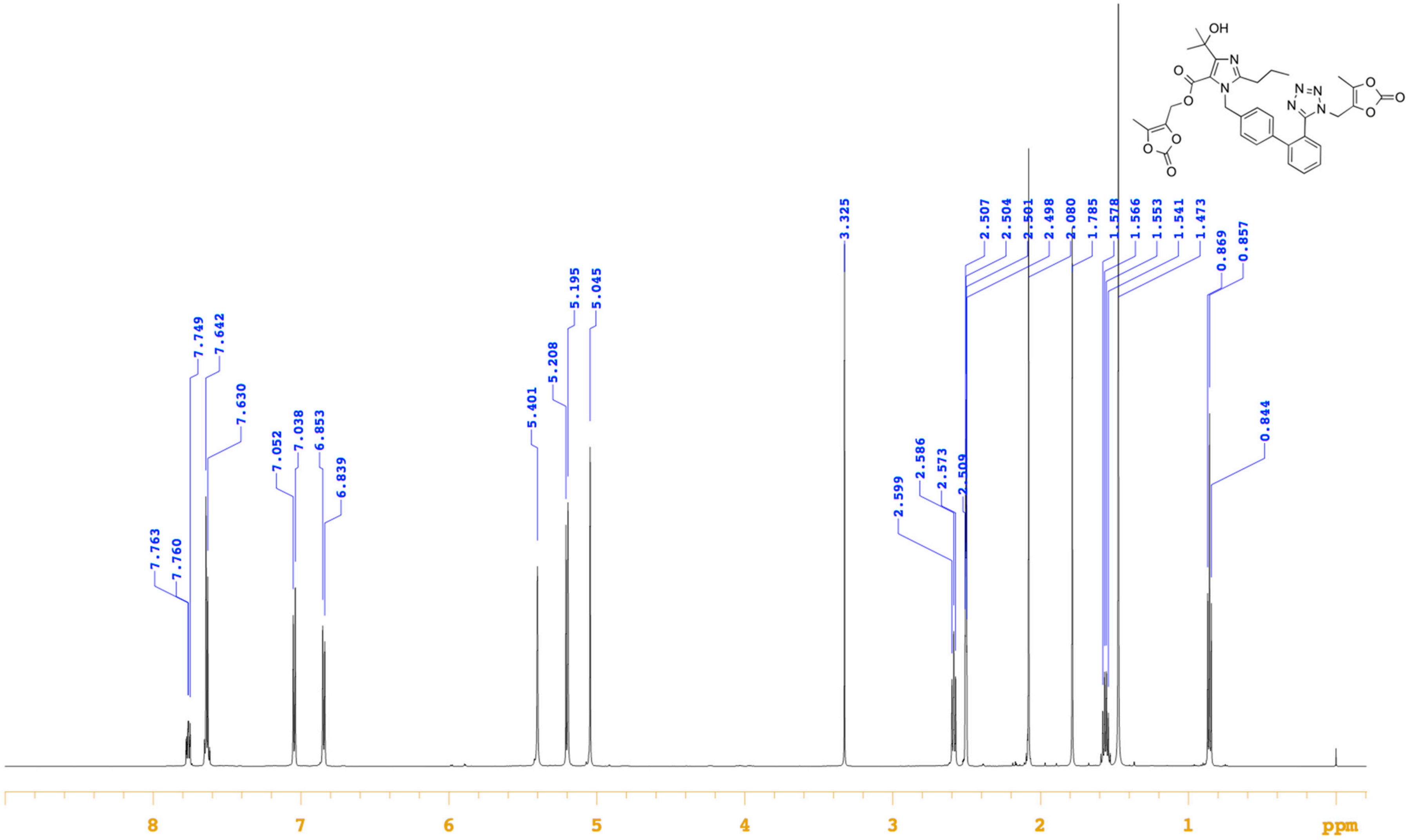

Figure S15. ¹H-NMR (600 MHz, DMSO-d_6, δ, ppm) spectrum of the *N*-1 substituted medoxomil impurity **10**.

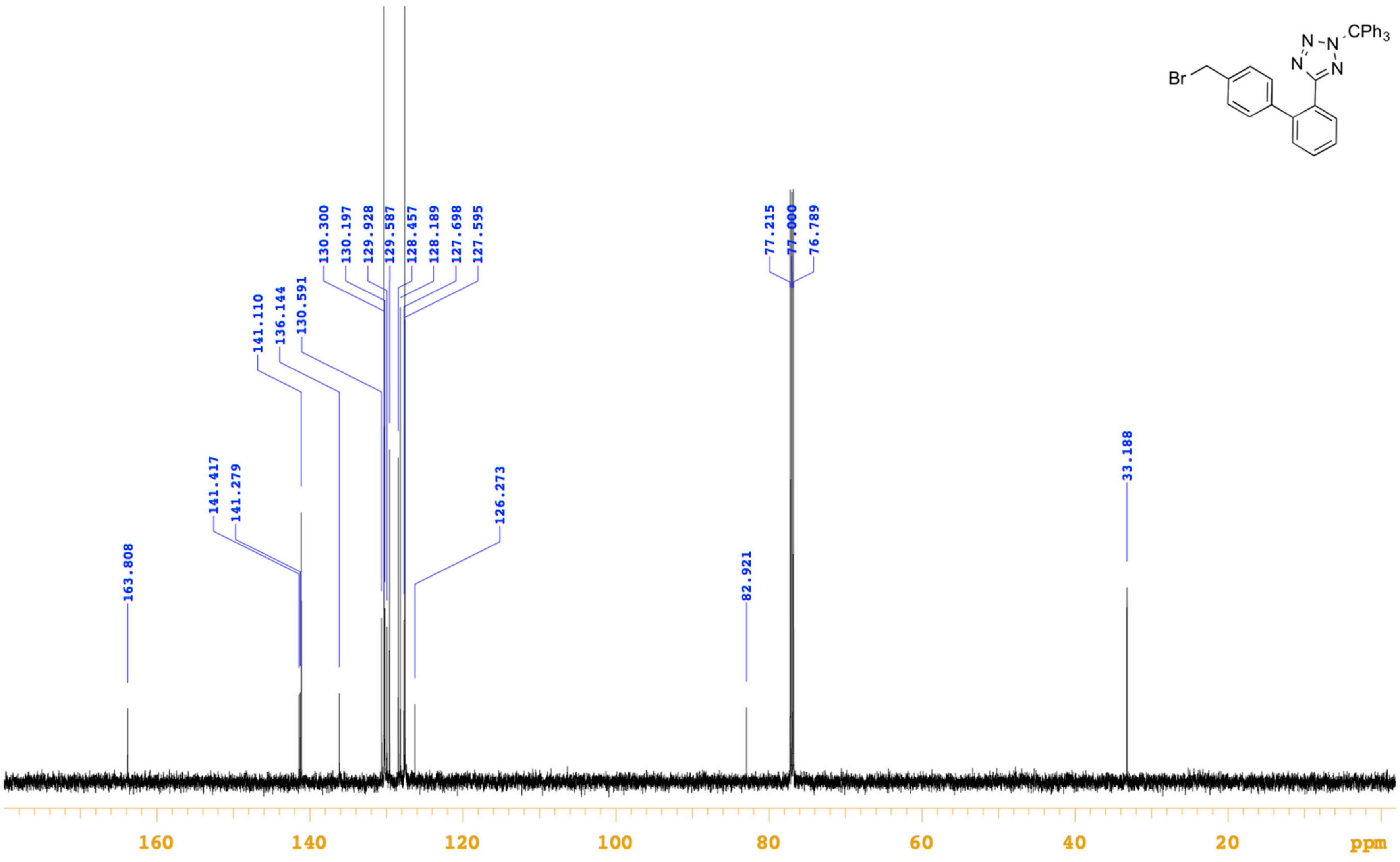

Figure S16. ^{13}C-NMR (150 MHz, $CDCl_3$, δ, ppm) spectrum of the bromide **2**.

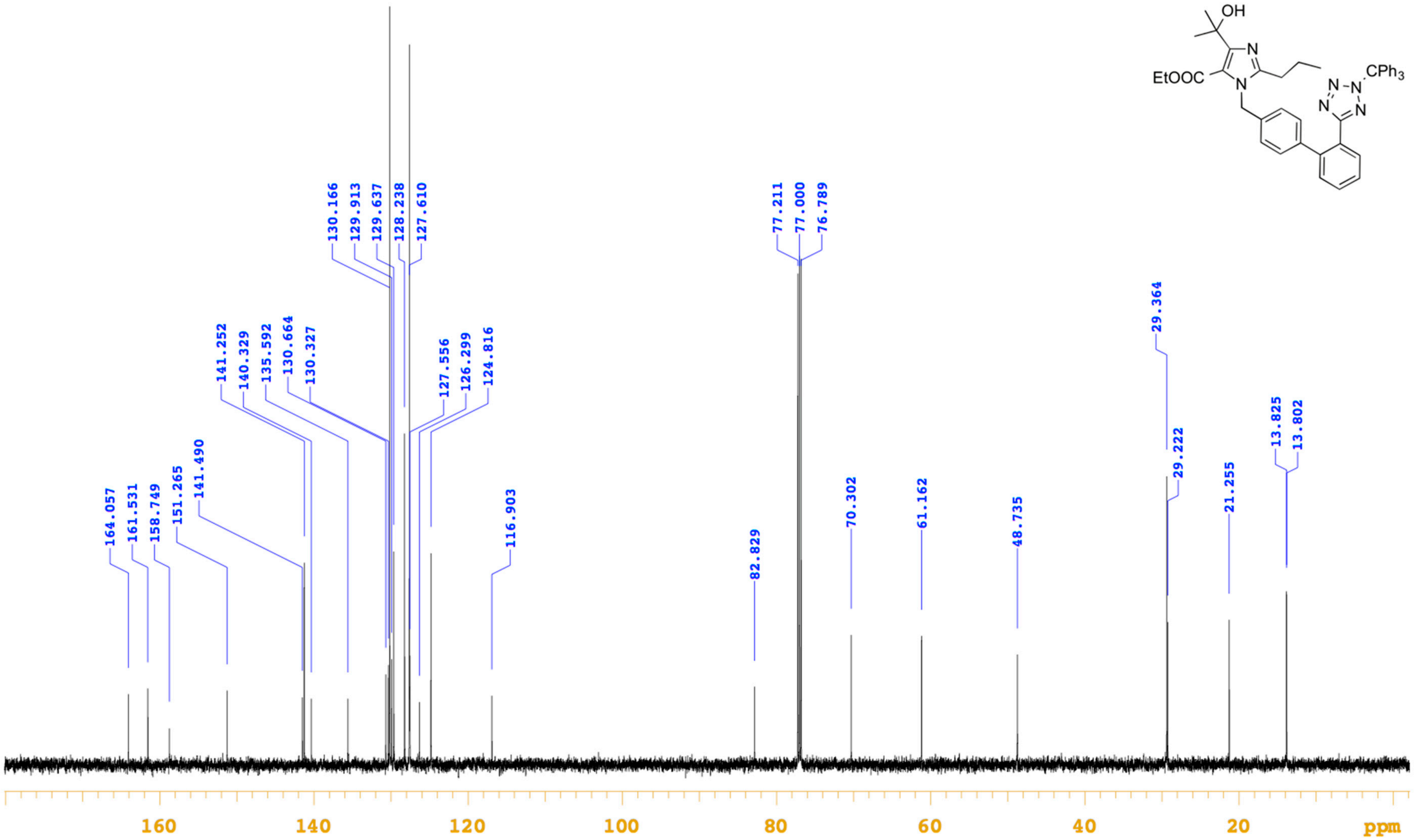

Figure S17. ^{13}C-NMR (150 MHz, $CDCl_3$, δ, ppm) spectrum of the ethyl ester **3**.

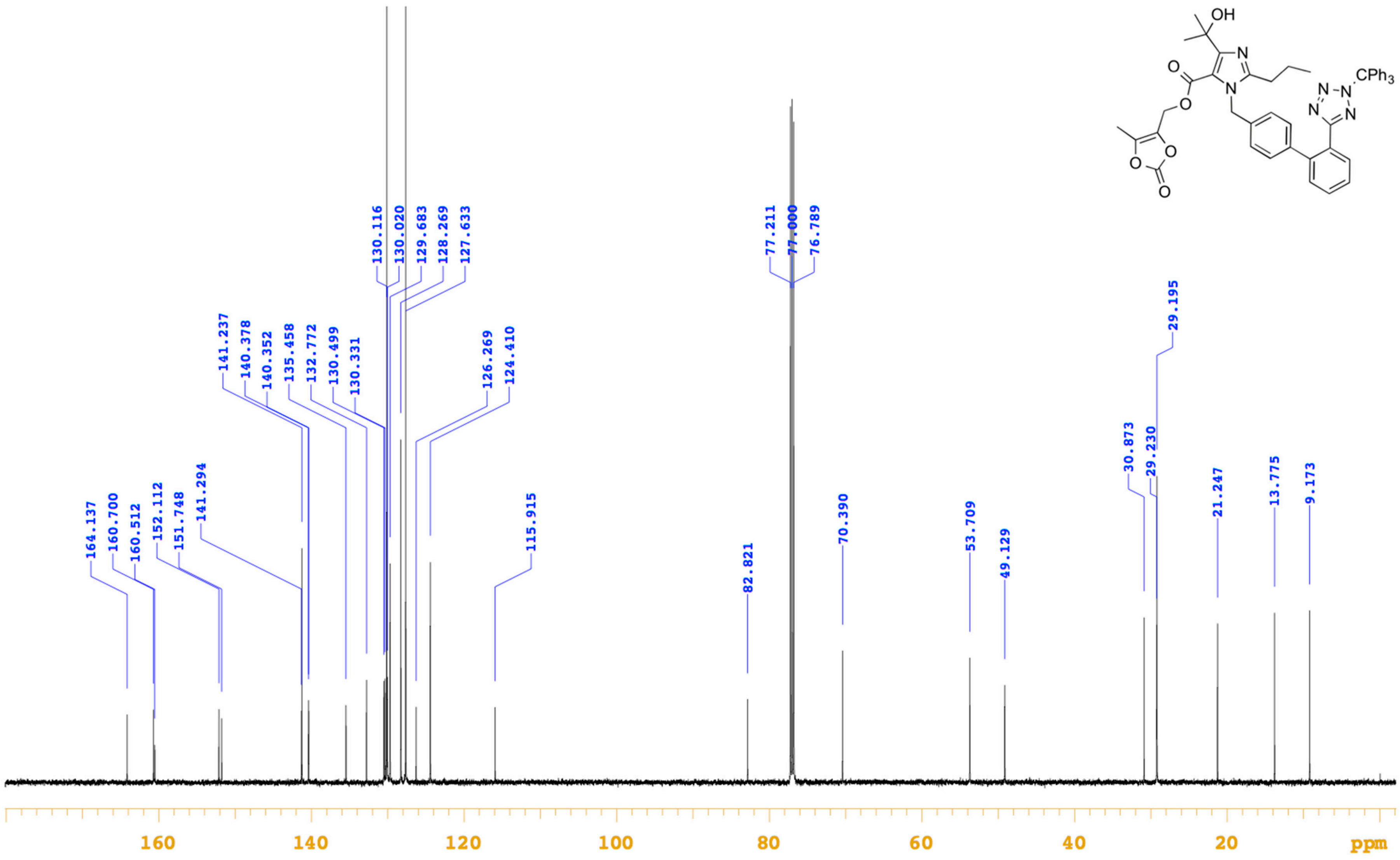

Figure S18. ^{13}C-NMR (150 MHz, $CDCl_3$, δ, ppm) spectrum of the medoxomil ester **6**.

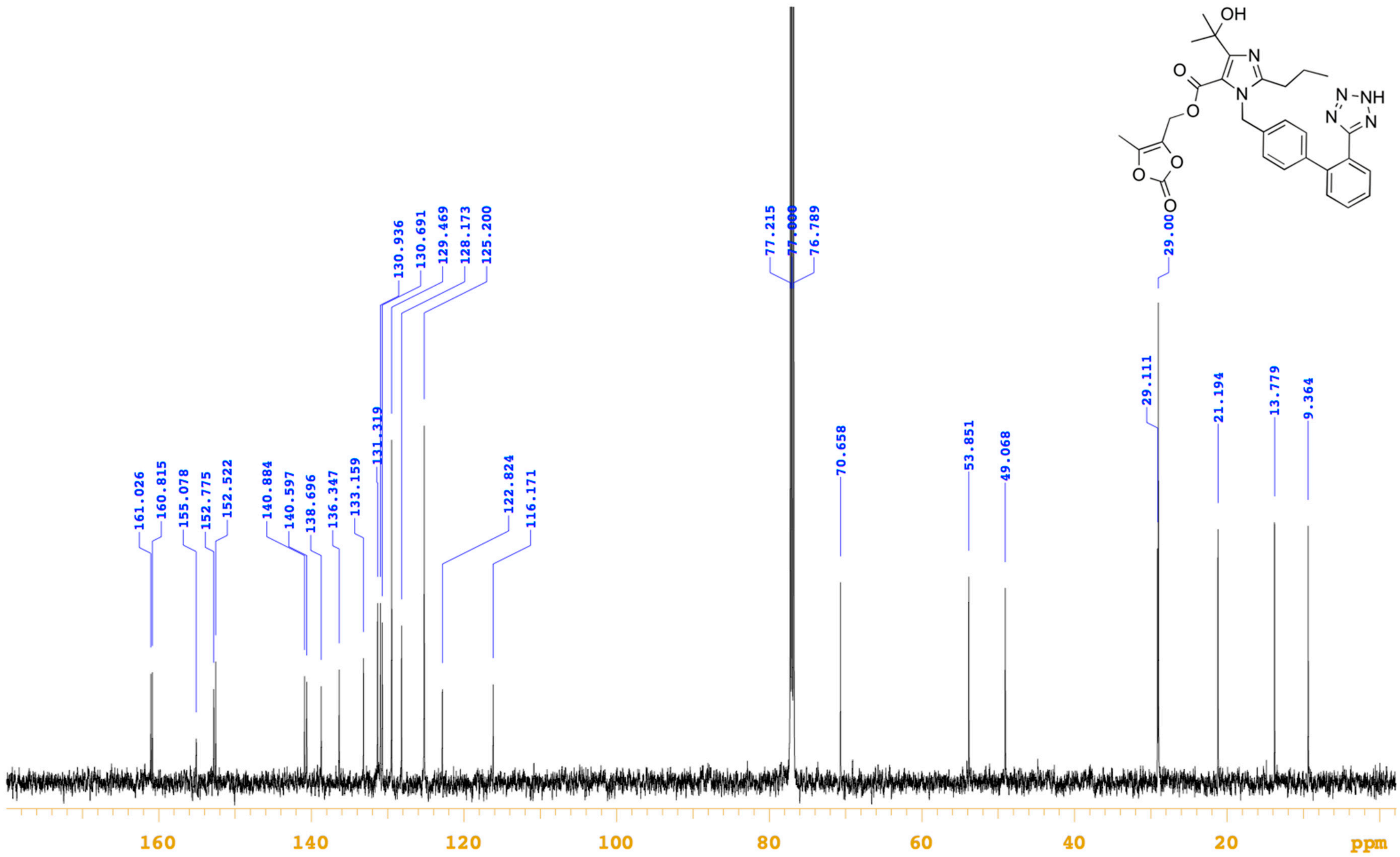

Figure S19. ¹³C-NMR (150 MHz, $CDCl_3$, δ, ppm) spectrum of the olmesartan medoxomil (**7**).

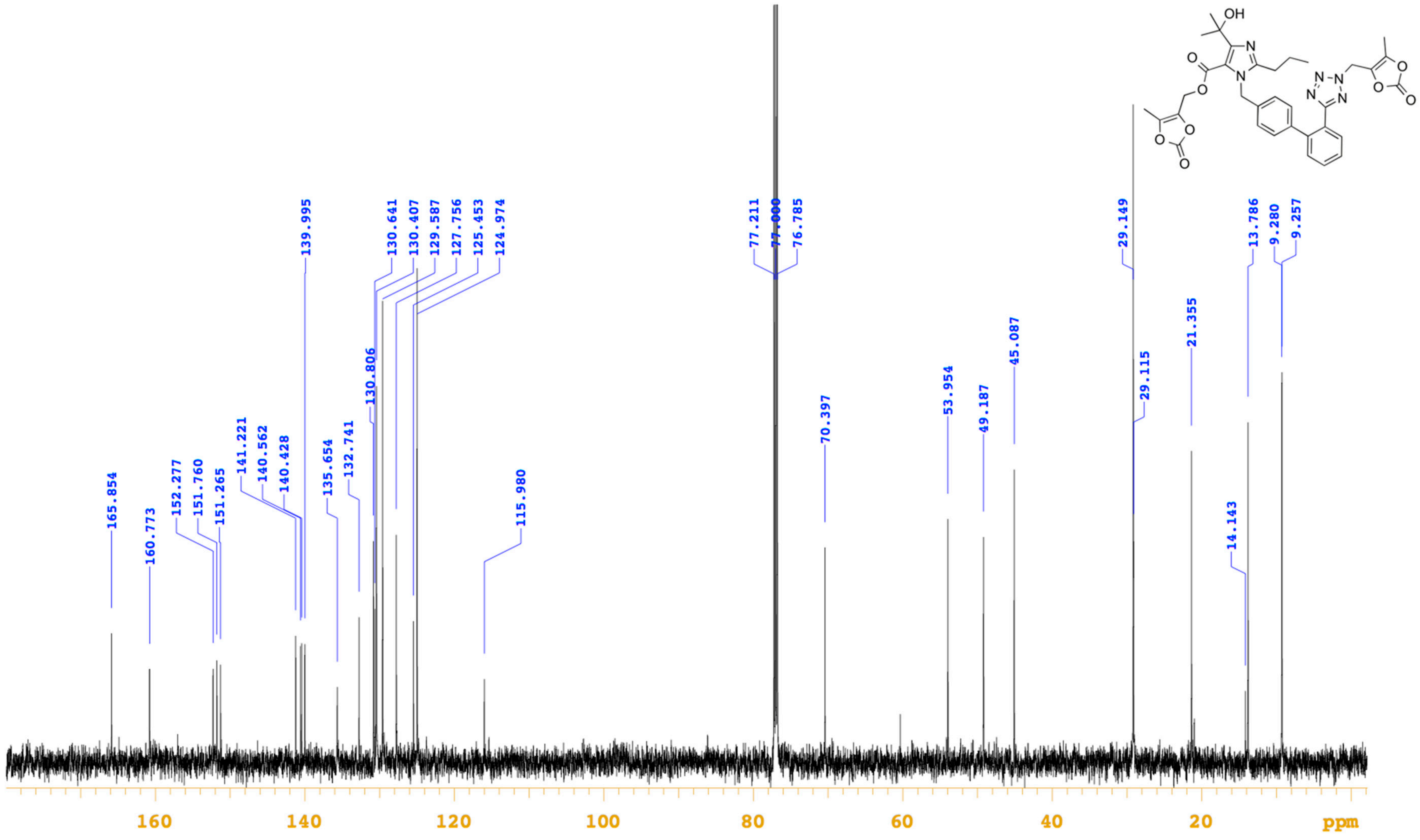

Figure S20. ^{13}C-NMR (150 MHz, $CDCl_3$, δ, ppm) spectrum of the *N*-2 substituted medoxomil impurity **9**.

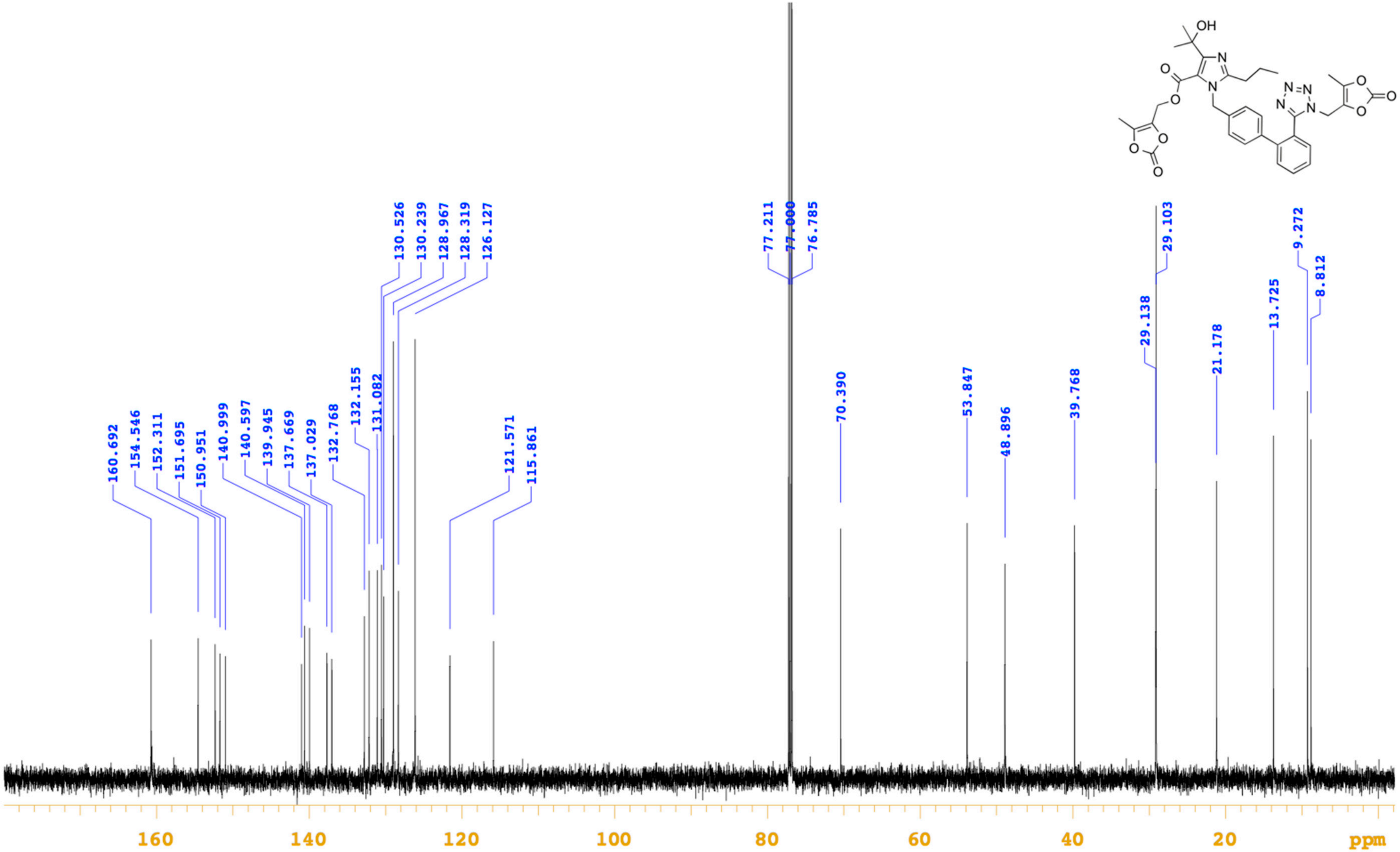

Figure S21. ^{13}C-NMR (150 MHz, $CDCl_3$, δ, ppm) spectrum of the *N*-1 substituted medoxomil impurity **10**.

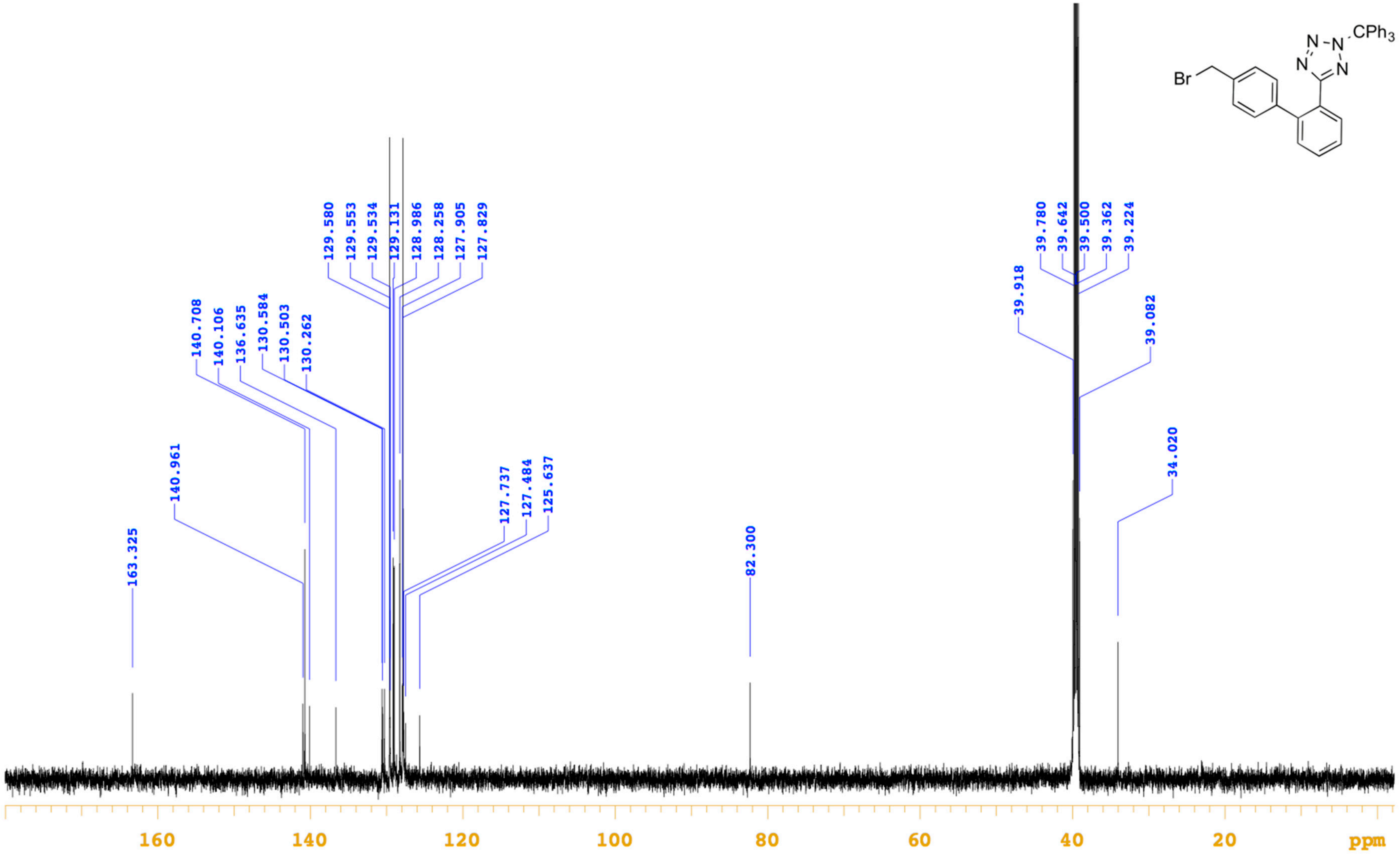

Figure S22. ^{13}C-NMR (150 MHz, DMSO-d_6, δ, ppm) spectrum of the bromide **2**.

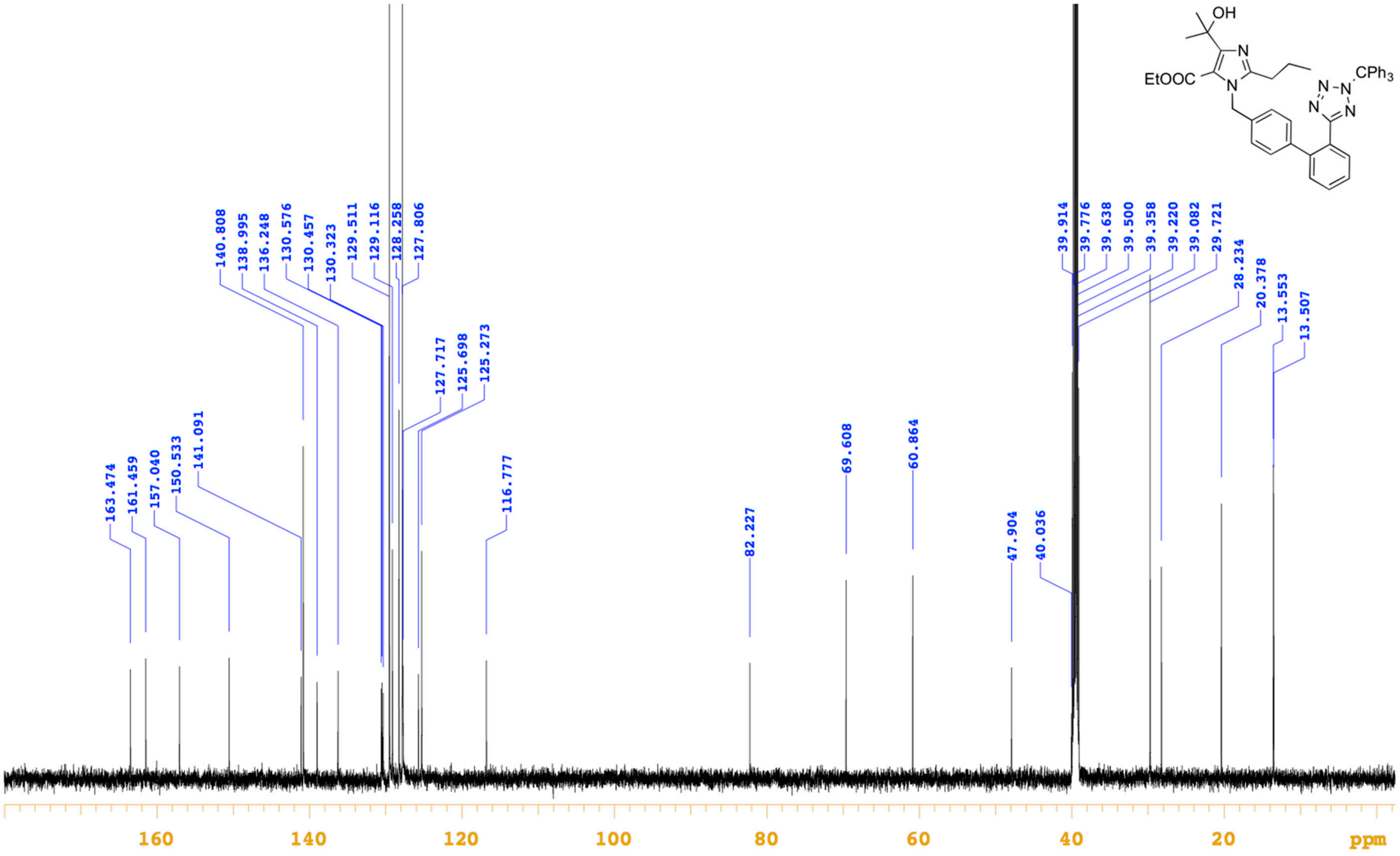

Figure S23. ^{13}C-NMR (150 MHz, DMSO-d_6, δ, ppm) spectrum of the ethyl ester **3**.

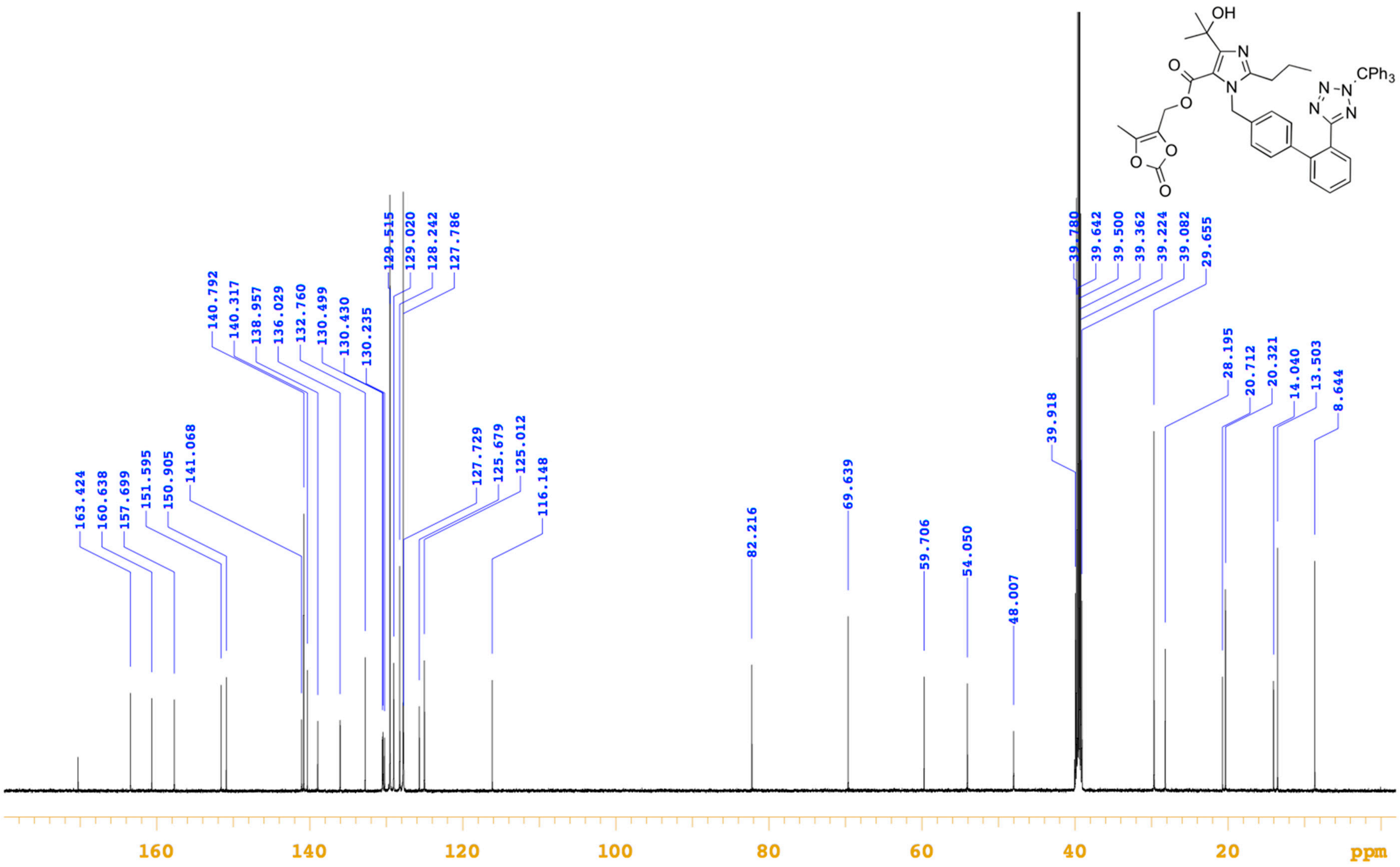

Figure S24. ^{13}C-NMR (150 MHz, DMSO-d_6, δ, ppm) spectrum of the medoxomil ester **6**.

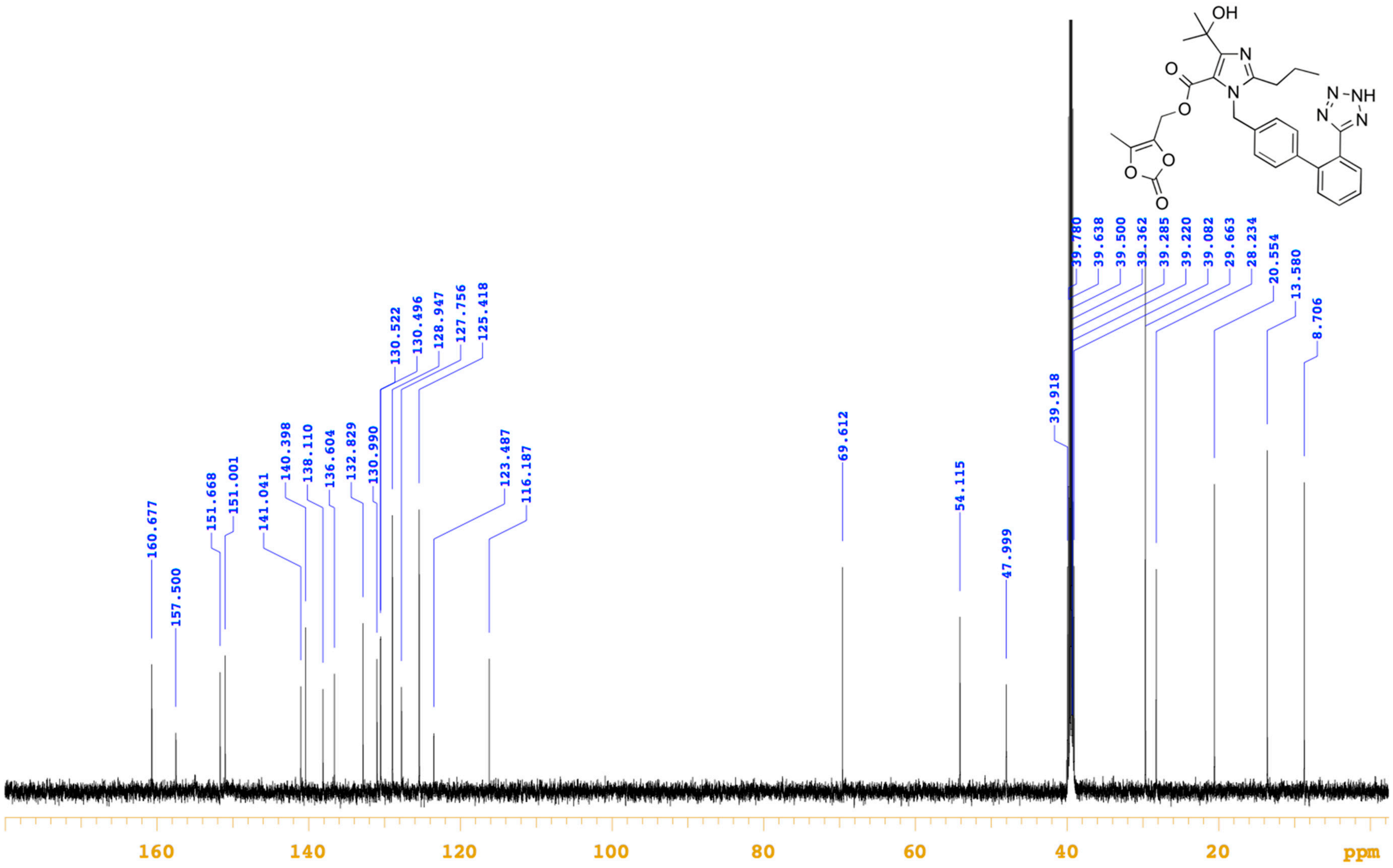

Figure S25. ^{13}C-NMR (150 MHz, DMSO-d_6, δ, ppm) spectrum of the olmesartan medoxomil (**7**).

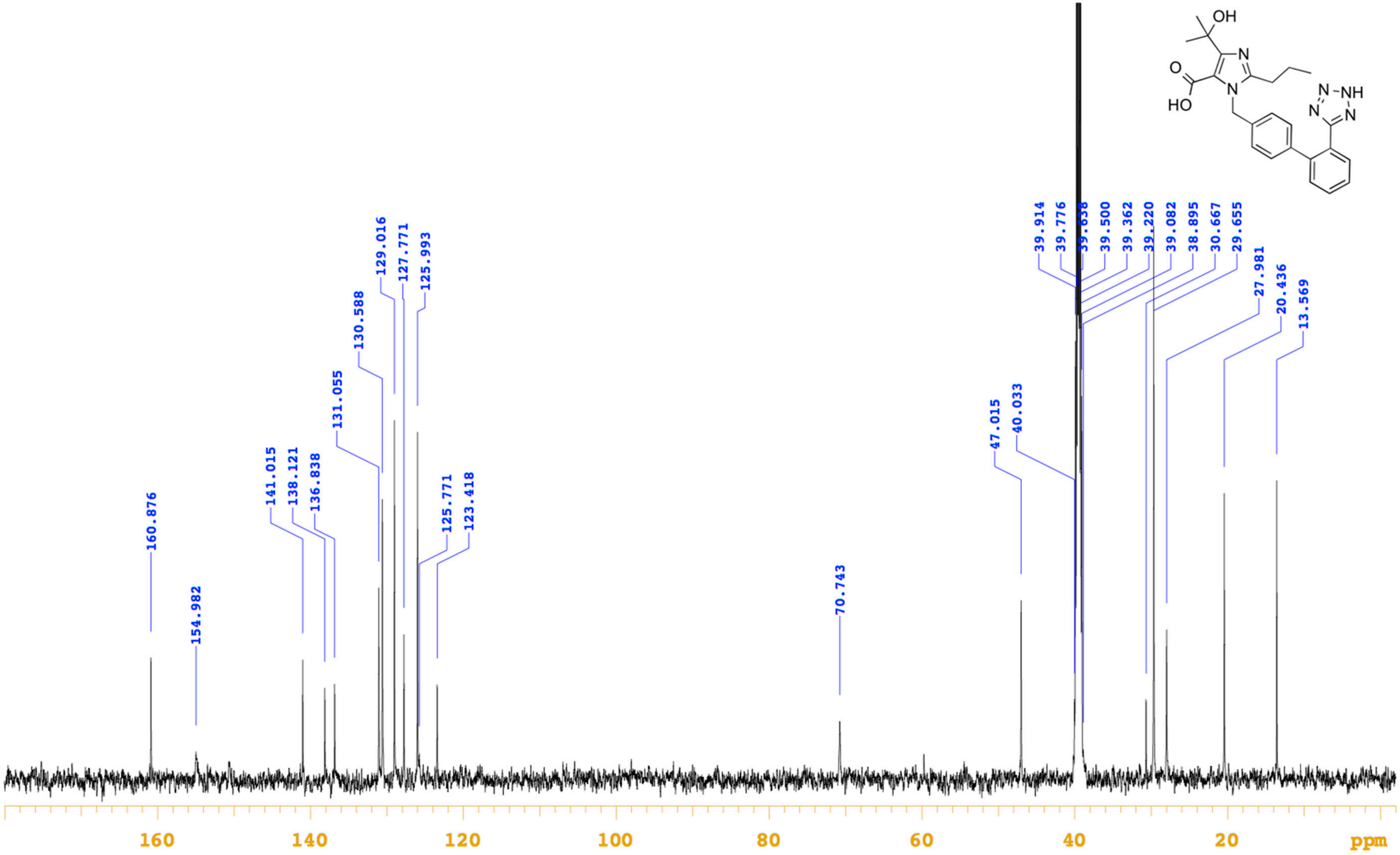

Figure S26. ^{13}C-NMR (150 MHz, DMSO-*d*$_6$, δ, ppm) spectrum of the olmesartan (**8**).

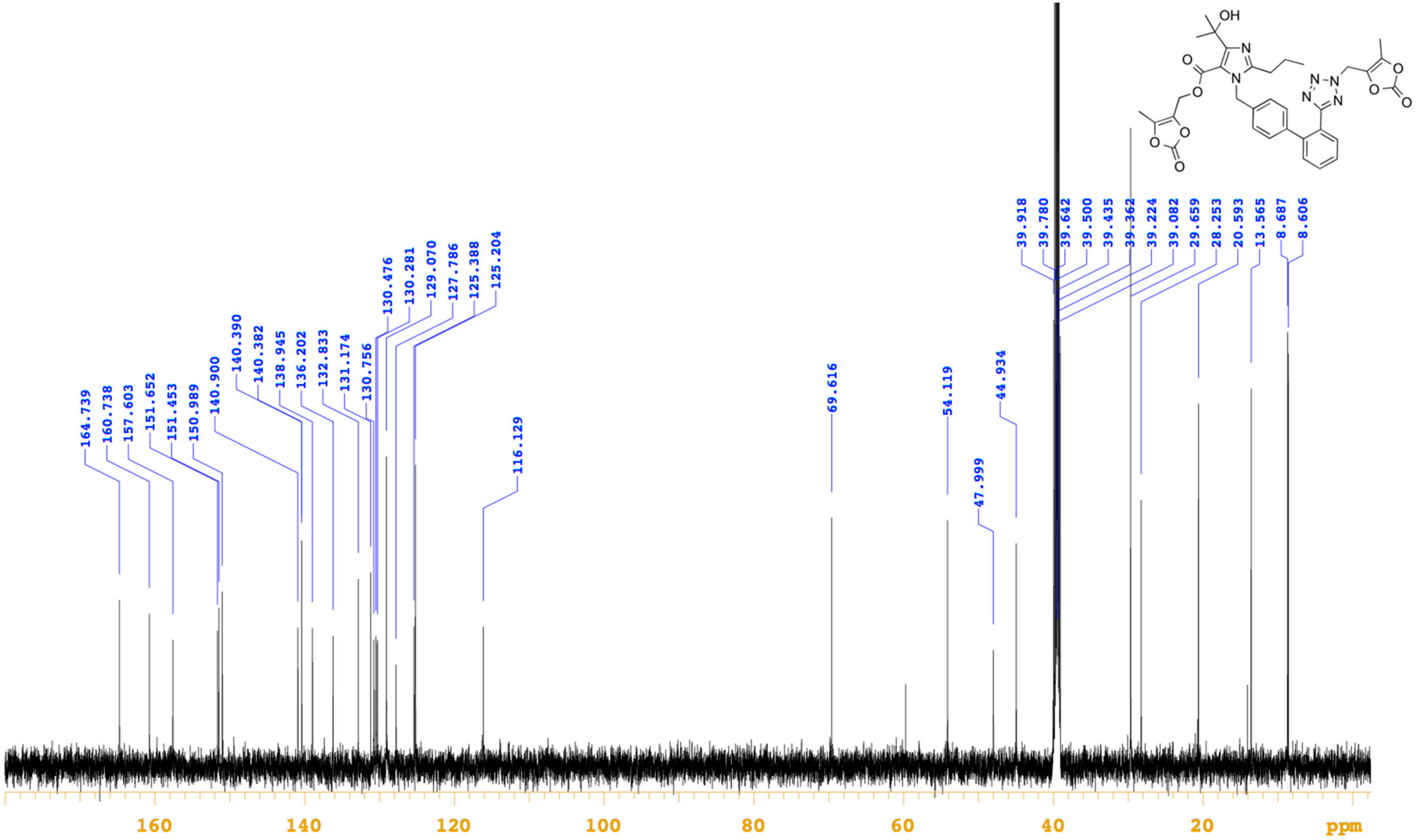

Figure S27. ^{13}C-NMR (150 MHz, DMSO-d_6, δ, ppm) spectrum of the *N*-2 substituted medoxomil impurity **9**.

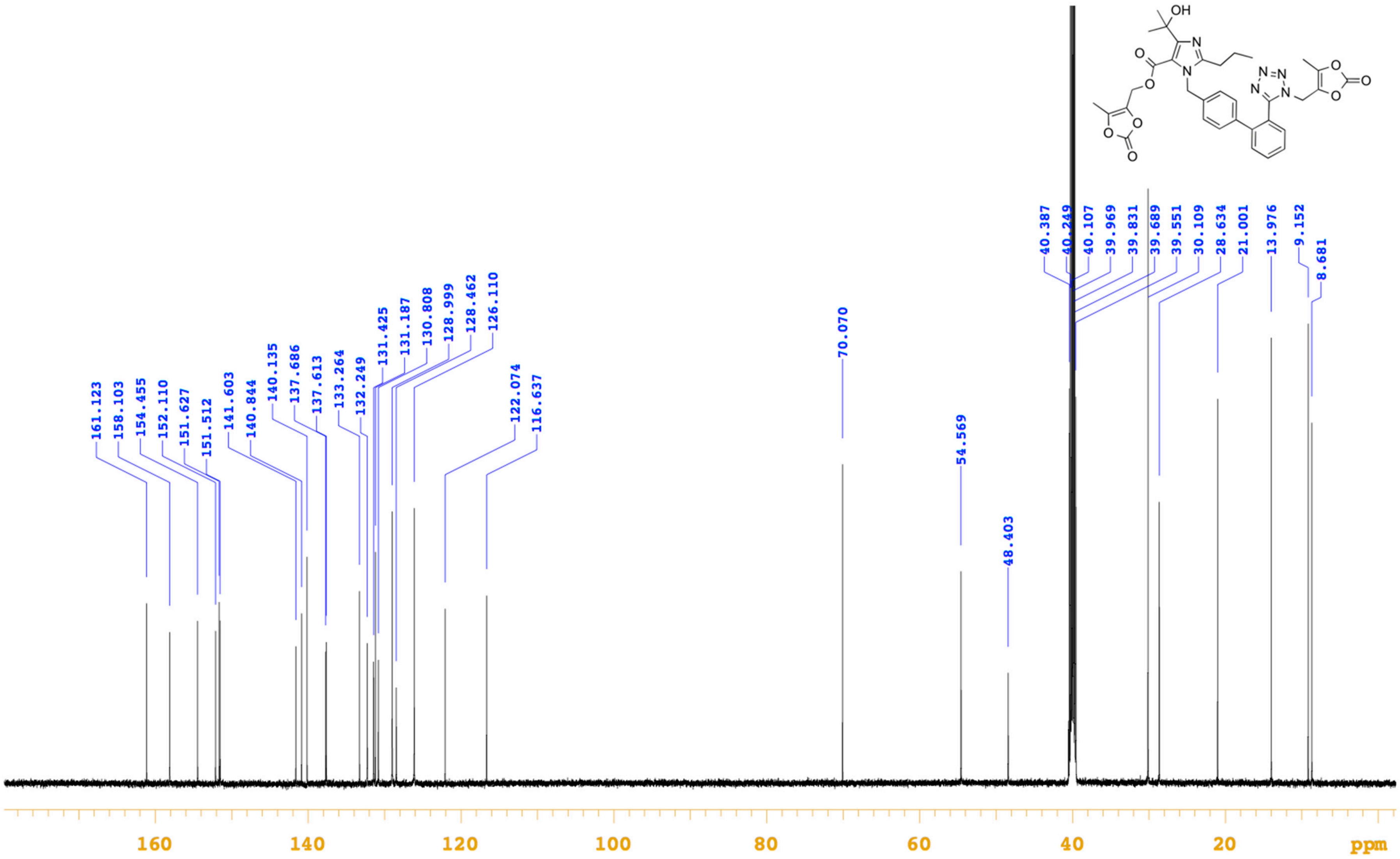

Figure S28. ^{13}C-NMR (150 MHz, DMSO-d_6, δ, ppm) spectrum of the *N*-1 substituted medoxomil impurity **10**.

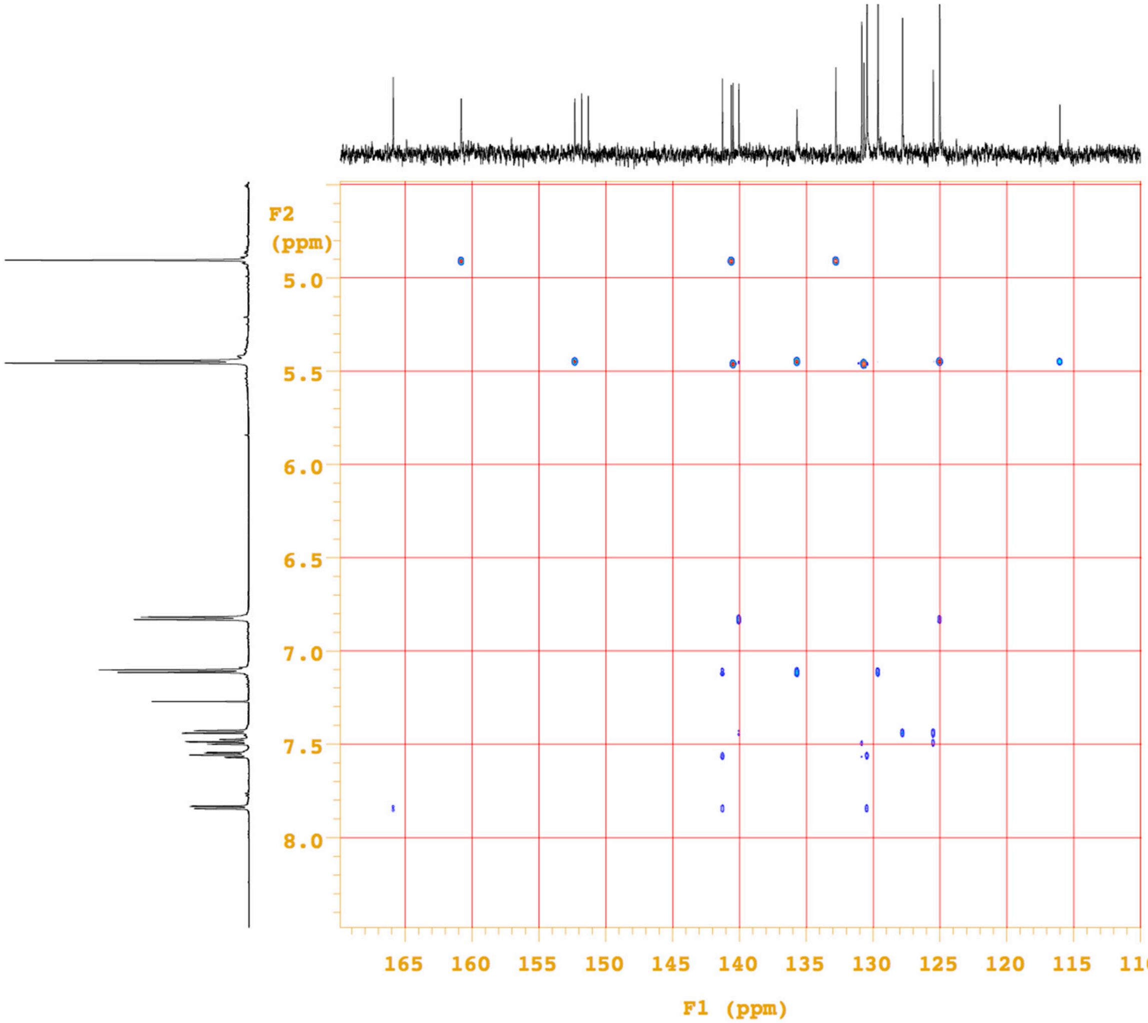

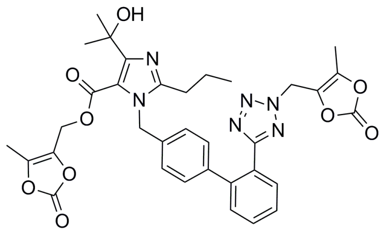

Figure S29. ^{1}H/^{13}C g-HMBC NMR spectrum of the *N*-2 substituted medoxomil derivative **9** in $CDCl_3$ solution.

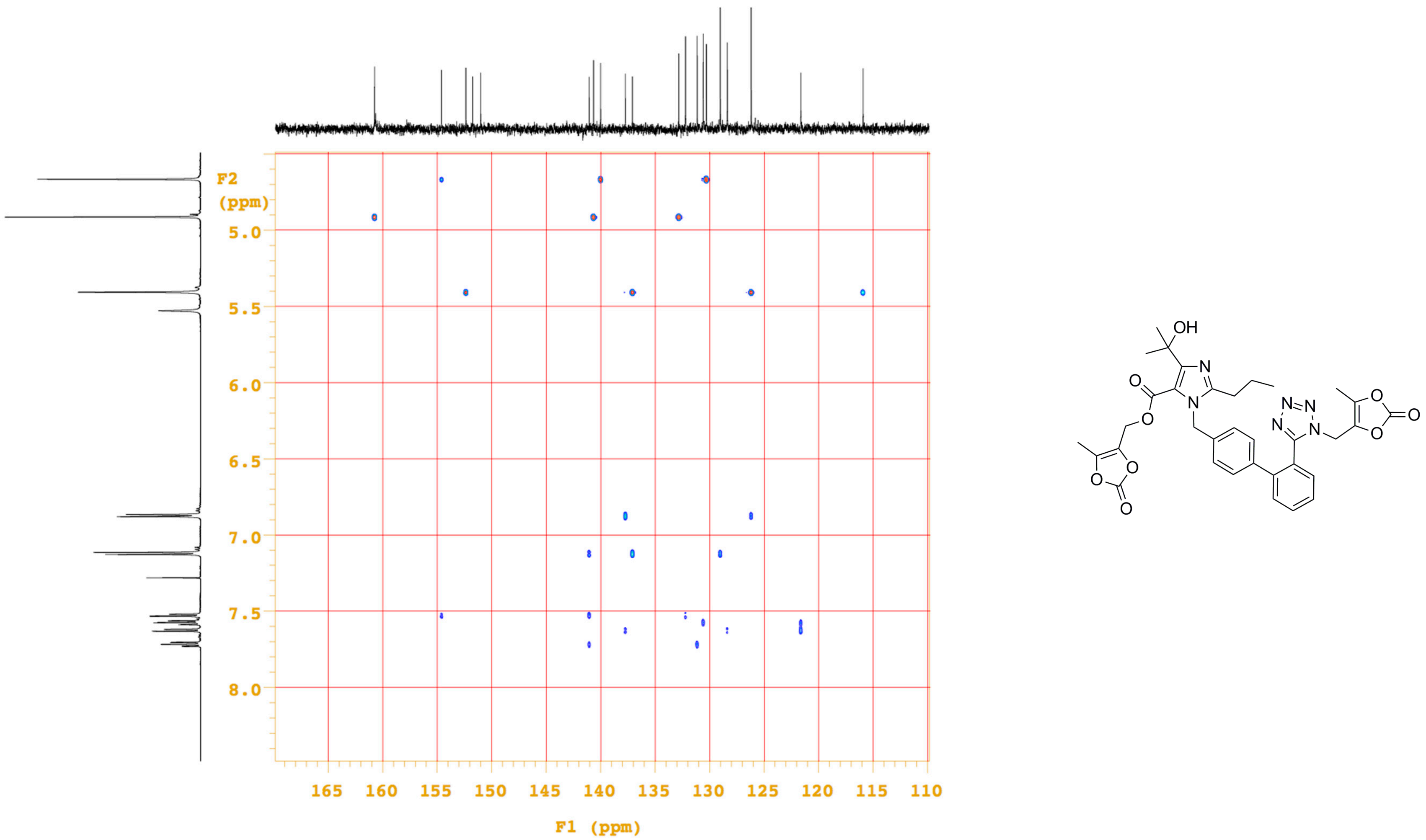

Figure S30. $^{1}H/^{13}C$ g-HMBC NMR spectrum of the *N*-1 substituted medoxomil derivative **10** in $CDCl_3$ solution.

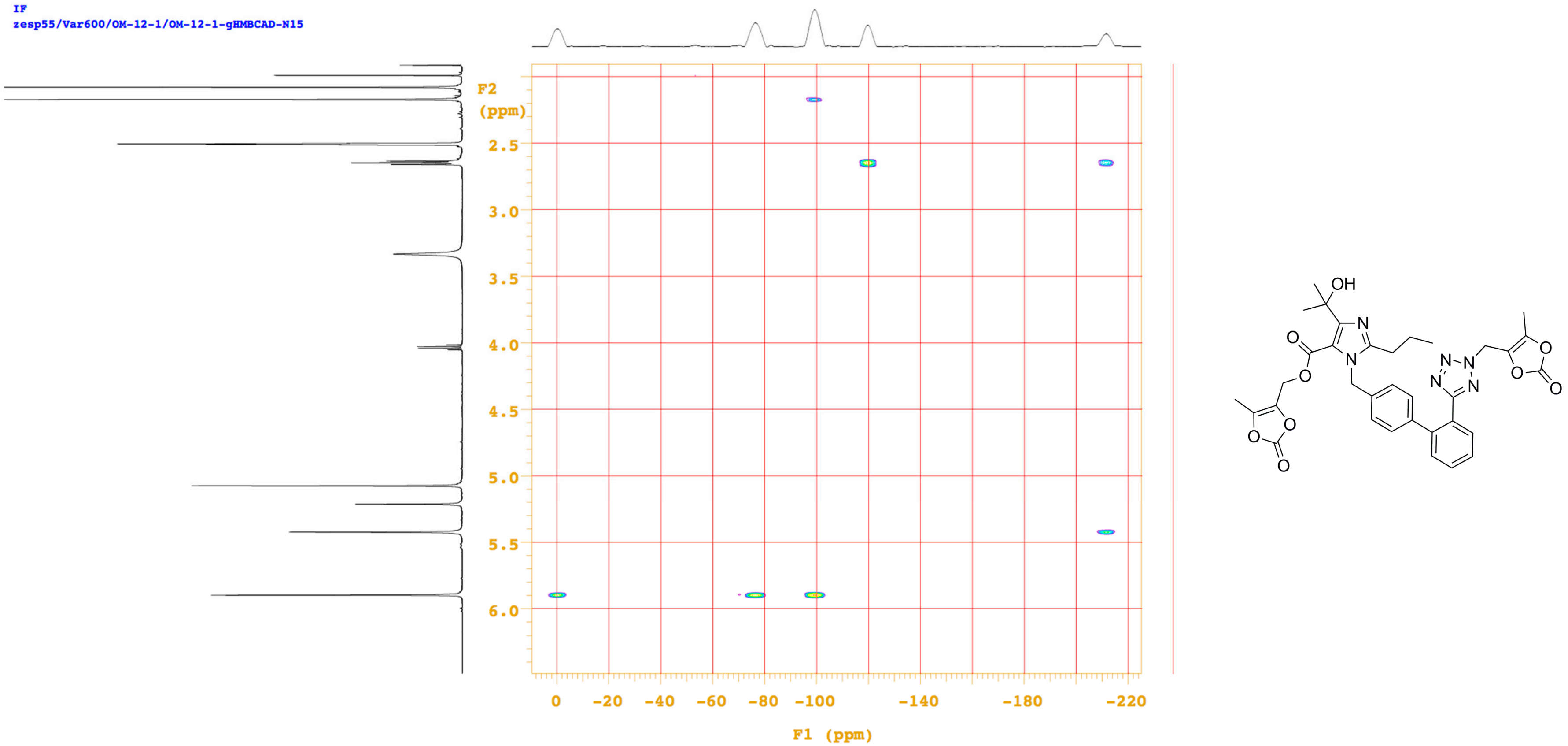

Figure S31. $^{1}H/^{15}N$ g-HMBC NMR spectrum of the *N*-2 substituted medoxomil derivative **9** in DMSO-d_6 solution.

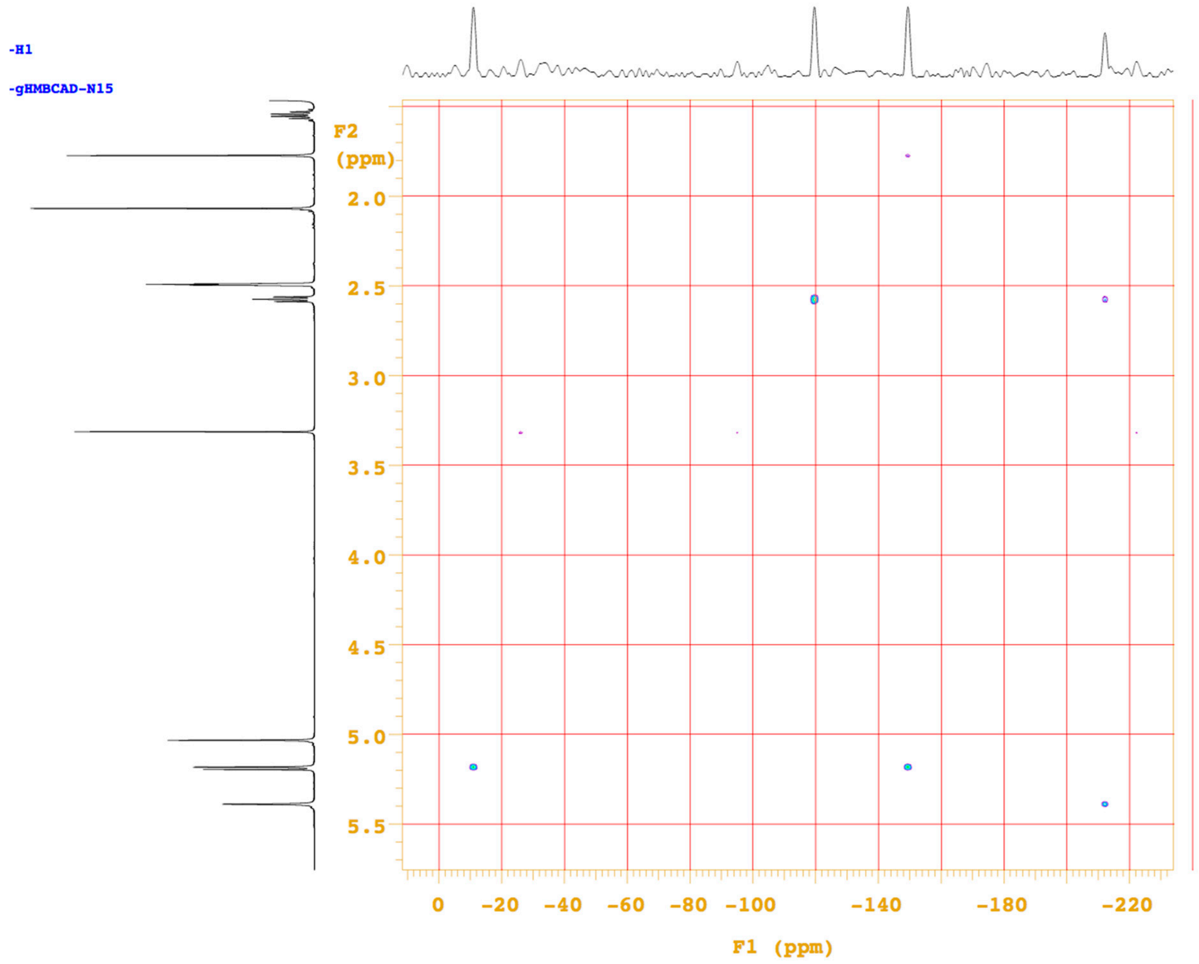

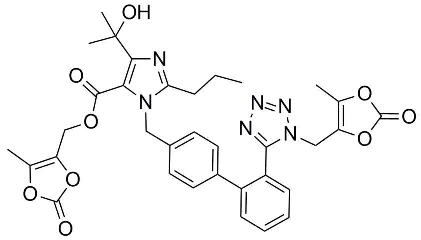

Figure S32. $^{1}H/^{15}N$ g-HMBC NMR spectrum of the *N*-1 substituted medoxomil derivative **10** in DMSO-d_6 solution.

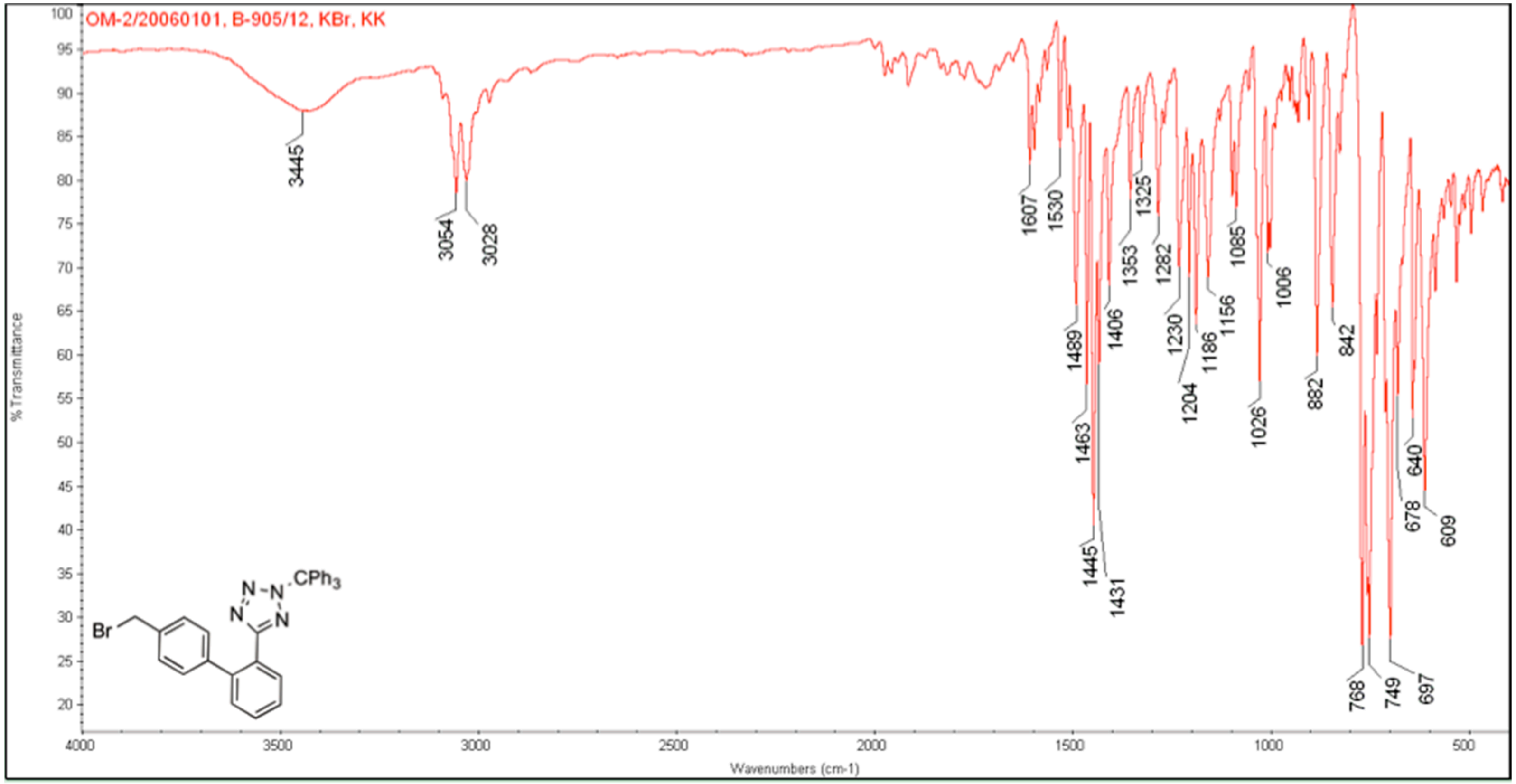

Figure S33. IR spectrum (KBr, ν, cm^{-1}) of the bromide **2**.

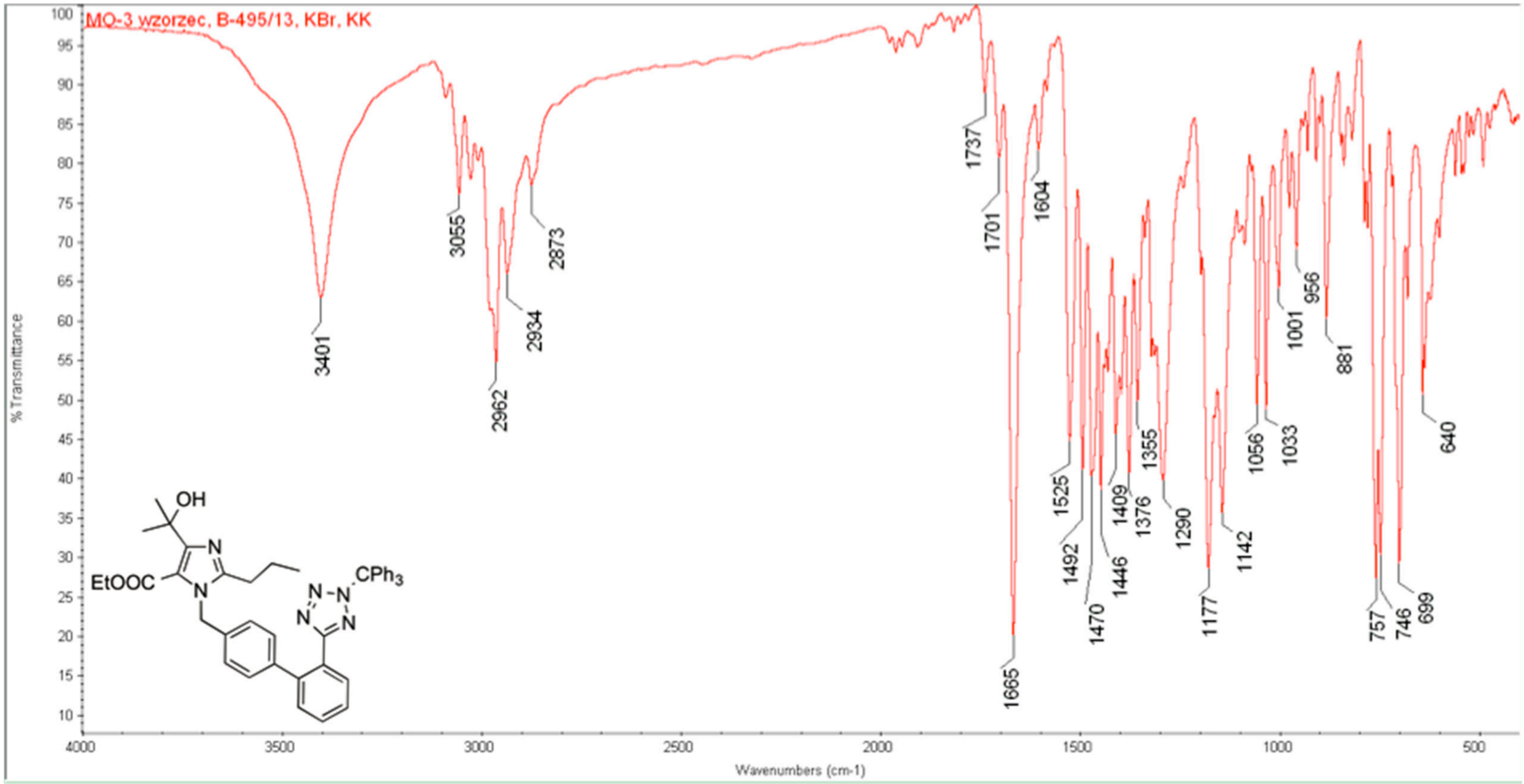

Figure S34. IR spectrum (KBr, ν, cm^{-1}) of the ethyl ester **3**.

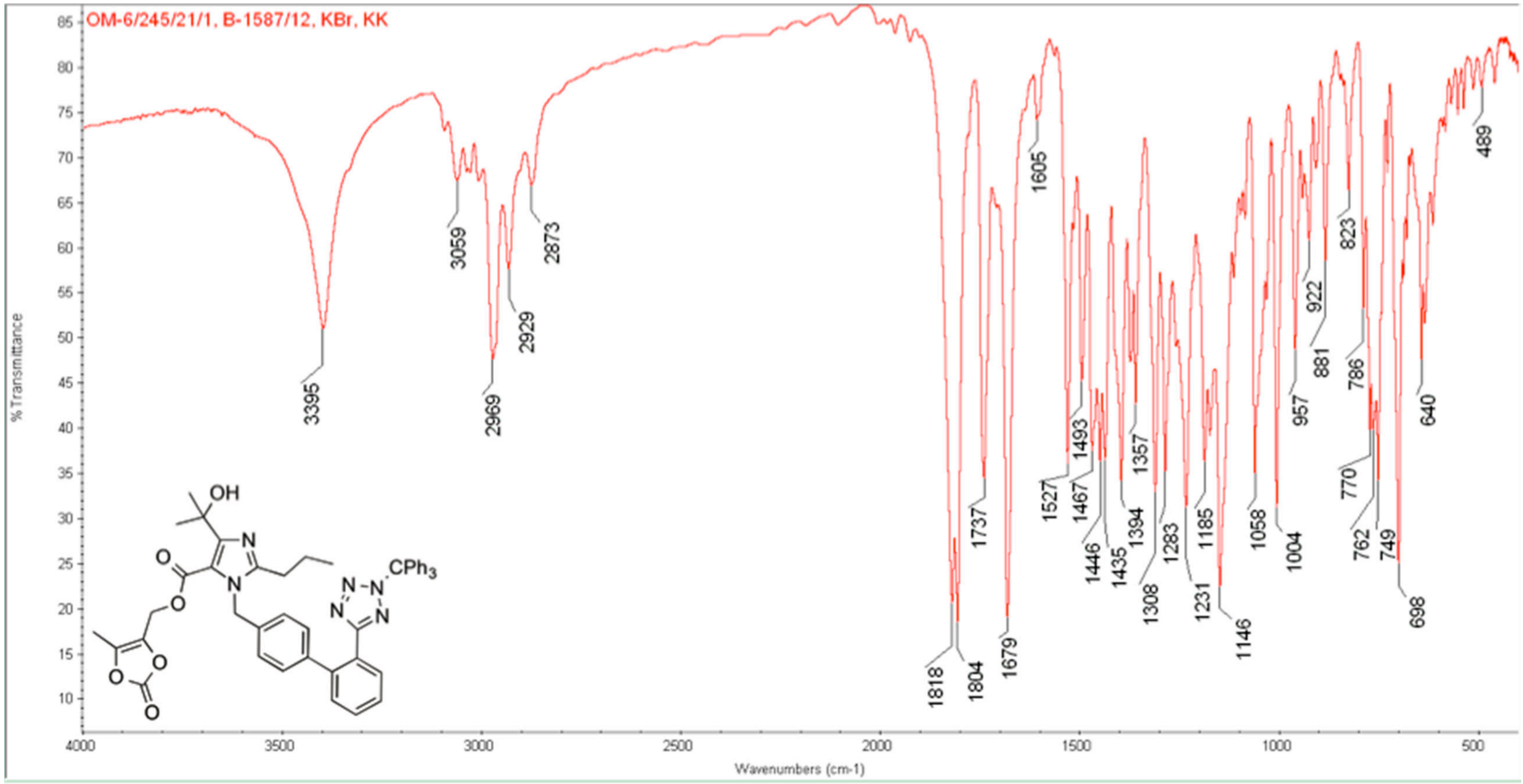

Figure S35. IR spectrum (KBr, ν, cm⁻¹) of the medoxomil ester **6**.

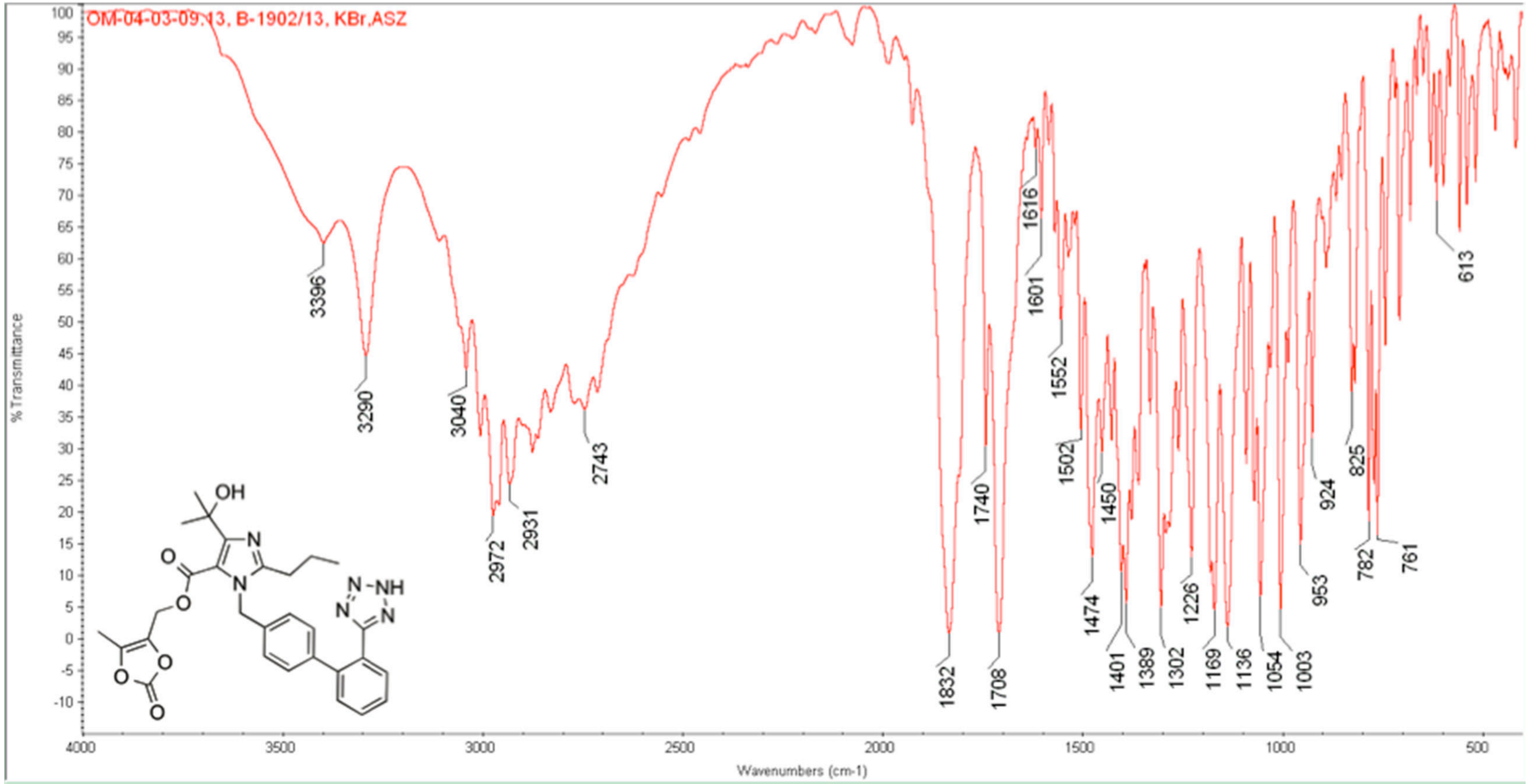

Figure S36. IR spectrum (KBr, ν, cm⁻¹) of the olmesartan medoxomil (**7**).

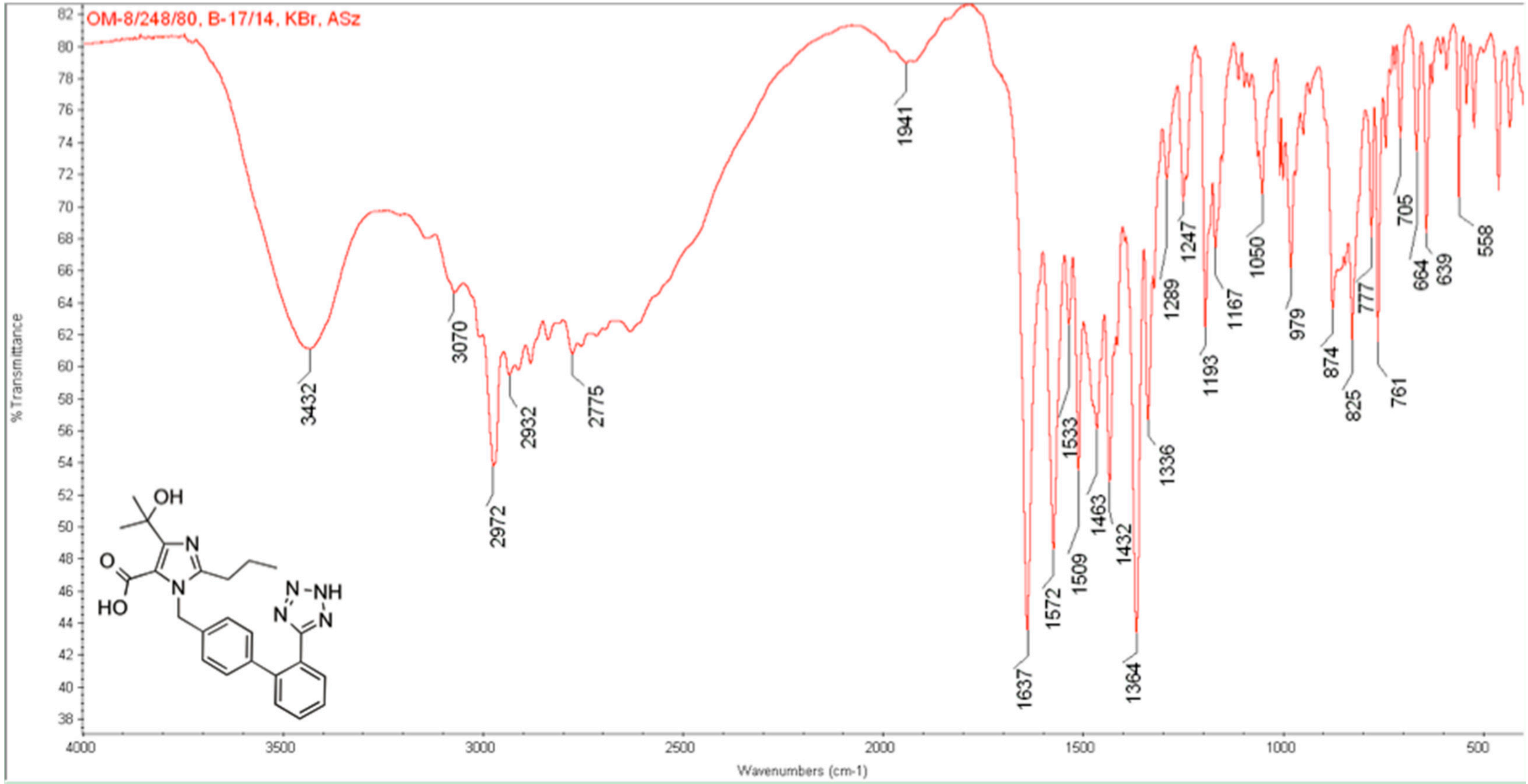

Figure S37. IR spectrum (KBr, ν, cm⁻¹) of the olmesartan (**8**).

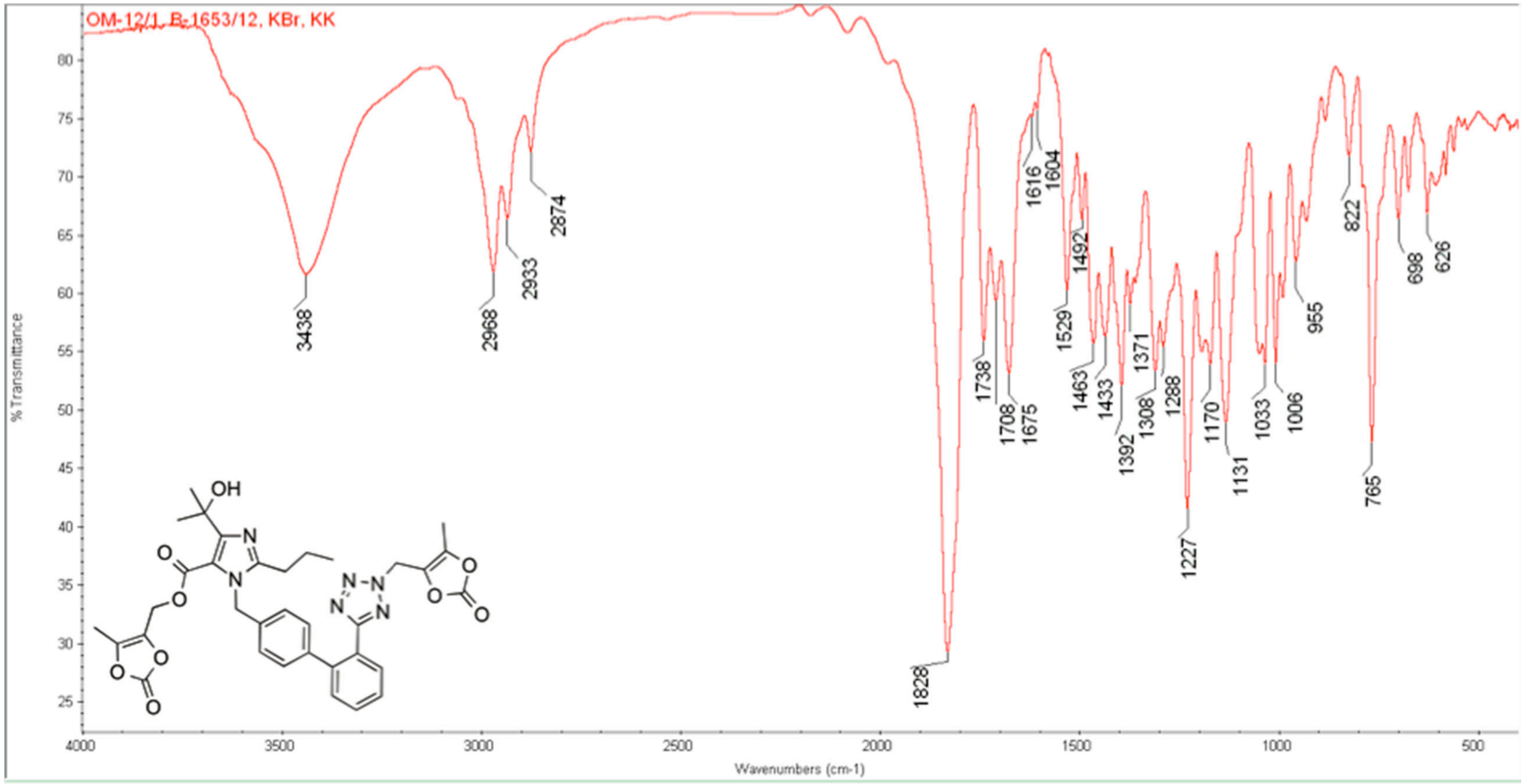

Figure S38. IR spectrum (KBr, ν, cm^{-1}) of the *N*-2 substituted medoxomil impurity **9**.

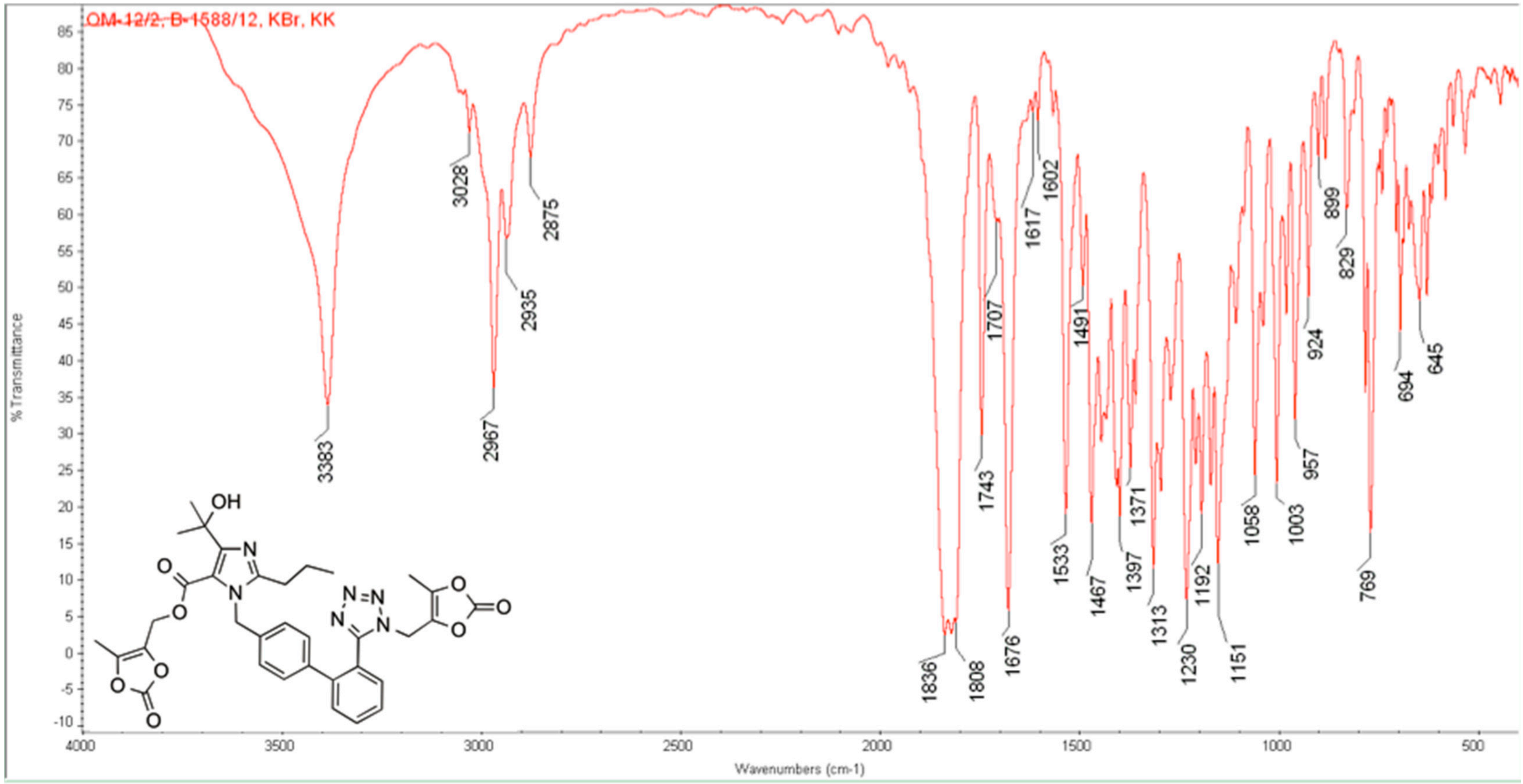

Figure S39. IR spectrum (KBr, ν, cm^{-1}) of the *N*-1 substituted medoxomil impurity **10.**

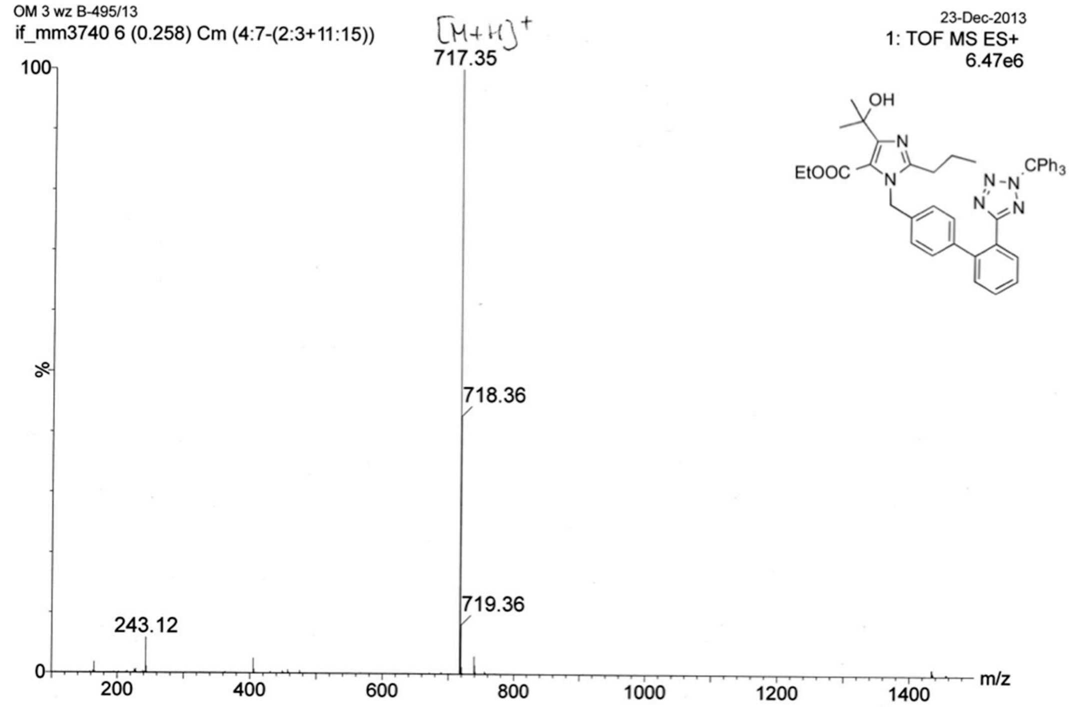

Single Mass Analysis

Tolerance = 3.0 mDa / DBE: min = -1.5, max = 100.0
Element prediction: Off
Number of isotope peaks used for i-FIT = 3

Monoisotopic Mass, Even Electron Ions
1087 formula(e) evaluated with 6 results within limits (up to 50 closest results for each mass)
Elements Used:
C: 0-100 H: 0-100 N: 0-10 O: 0-5 Na: 0-1
OM 3 wz B-495/13
if_mm3740 6 (0.258) AM2 (Ar,30000.0,0.00,0.00); ABS; Cm (4:7-(2:3+11:15))

23-Dec-2013
1: TOF MS ES+
1.69e+007

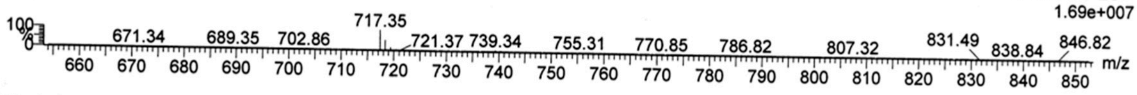

Minimum:				-1.5				
Maximum:		3.0	10.0	100.0				
Mass	Calc. Mass	mDa	PPM	DBE	i-FIT	Norm	Conf(%)	Formula
717.3550	717.3553	-0.3	-0.4	26.5	592.4	6.168	0.21	C45 H45 N6 O3
	717.3556	-0.6	-0.8	22.5	594.1	7.784	0.04	C47 H50 O5 Na
	717.3569	-1.9	-2.6	27.5	597.2	10.937	0.00	C48 H46 N4 O Na
	717.3529	2.1	2.9	23.5	586.3	0.003	99.75	C43 H46 N6 O3 Na
	717.3521	2.9	4.0	34.5	602.0	15.699	0.00	C56 H45
	717.3580	-3.0	-4.2	25.5	599.6	13.290	0.00	C49 H49 O5

Figure S40. HRMS spectrum of the ethyl ester **3**.

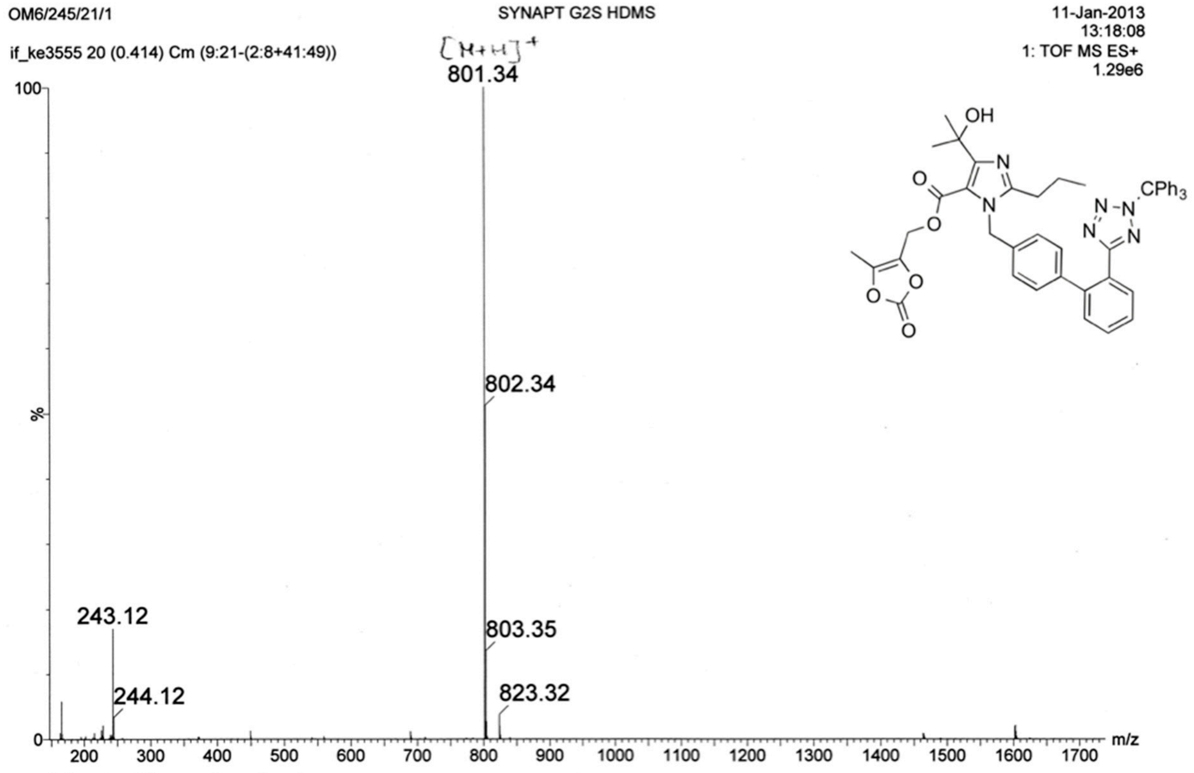

Single Mass Analysis

Tolerance = 10.0 mDa / DBE: min = -1.5, max = 100.0
Element prediction: Off
Number of isotope peaks used for i-FIT = 4

Monoisotopic Mass, Even Electron Ions
1110 formula(e) evaluated with 15 results within limits (up to 50 closest results for each mass)
Elements Used:
C: 0-200 H: 0-200 N: 0-10 O: 0-10

Minimum:				-1.5				
Maximum:		10.0	5.0	100.0				
Mass	Calc. Mass	mDa	PPM	DBE	i-FIT	Norm	Conf(%)	Formula
801.3387	801.3387	0.0	0.0	24.5	759.5	6.414	0.16	C47 H49 N2 O10
	801.3401	-1.4	-1.7	29.5	758.5	5.427	0.44	C48 H45 N6 O6
	801.3369	1.8	2.2	37.5	760.5	7.450	0.06	C59 H45 O3
	801.3414	-2.7	-3.4	34.5	758.2	5.154	0.58	C49 H41 N10 O2
	801.3360	2.7	3.4	25.5	762.4	9.312	0.01	C43 H45 N8 O8
	801.3427	-4.0	-5.0	28.5	753.2	0.156	85.59	C52 H49 O8
	801.3342	4.5	5.6	38.5	758.4	5.334	0.48	C55 H41 N6 O
	801.3441	-5.4	-6.7	33.5	755.8	2.741	6.45	C53 H45 N4 O4
	801.3328	5.9	7.4	33.5	755.9	2.832	5.89	C54 H45 N2 O5
	801.3320	6.7	8.4	21.5	766.6	13.537	0.00	C38 H45 N10 O10
	801.3454	-6.7	-8.4	38.5	759.4	6.281	0.19	C54 H41 N8
	801.3302	8.5	10.6	34.5	759.8	6.750	0.12	C50 H41 N8 O3
	801.3473	-8.6	-10.7	25.5	765.5	12.369	0.00	C42 H45 N10 O7
	801.3481	-9.4	-11.7	37.5	763.2	10.104	0.00	C58 H45 N2 O2
	801.3288	9.9	12.4	29.5	761.1	8.005	0.03	C49 H45 N4 O7

Figure S41. HRMS spectrum of the medoxomil ester **6**.

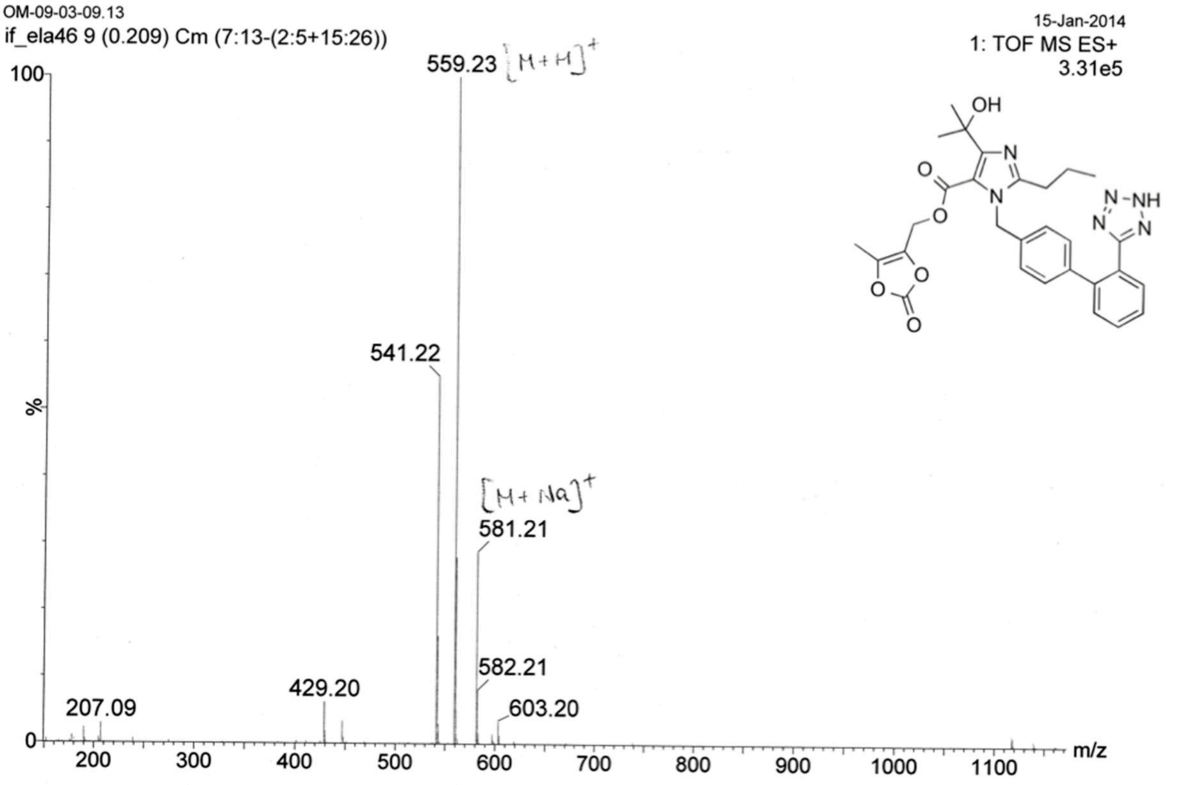

Single Mass Analysis

Tolerance = 5.0 PPM / DBE: min = -1.5, max = 100.0
Element prediction: Off
Number of isotope peaks used for i-FIT = 3

Monoisotopic Mass, Even Electron Ions
525 formula(e) evaluated with 4 results within limits (up to 50 closest results for each mass)
Elements Used:
C: 0-50 H: 0-50 N: 0-10 O: 0-10
OM-09-03-09.13 15-Jan-2014
if_ela46 9 (0.209) AM2 (Ar,30000.0,0.00,0.00); ABS; Cm (7:13-(2:5+15:26)) 1: TOF MS ES+
1.21e+006

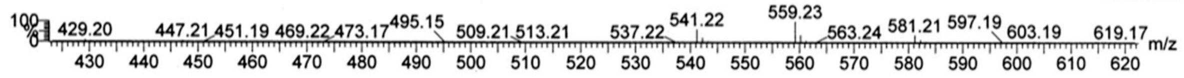

Mass	Calc. Mass	mDa	PPM	DBE	i-FIT	Norm	Conf(%)	Formula
Minimum:				-1.5				
Maximum:		3.0	5.0	100.0				
559.2306	559.2305	0.1	0.2	17.5	567.6	0.111	89.47	C29 H31 N6 O6
	559.2318	-1.2	-2.1	22.5	573.0	5.509	0.41	C30 H27 N10 O2
	559.2292	1.4	2.5	12.5	569.8	2.291	10.12	C28 H35 N2 O10
	559.2332	-2.6	-4.6	16.5	577.0	9.510	0.01	C33 H35 O8

Figure S42. HRMS spectrum of the olmesartan medoxomil (**7**).

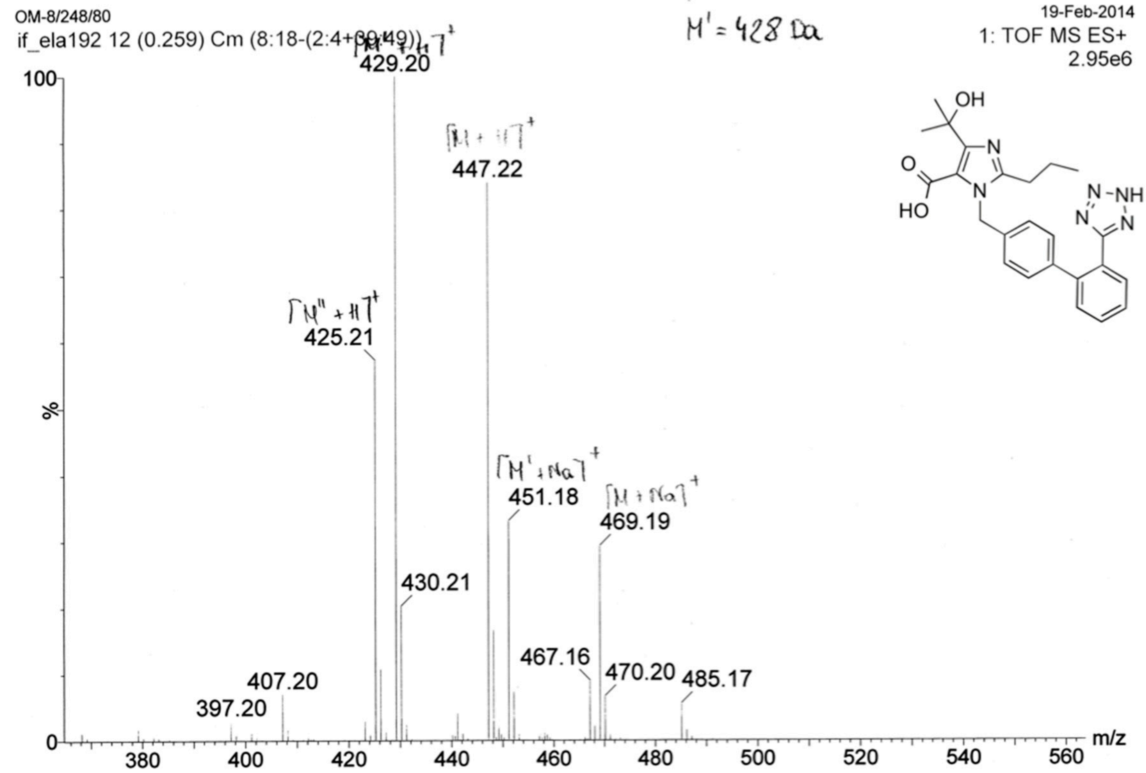

Single Mass Analysis
Tolerance = 5.0 PPM / DBE: min = -1.5, max = 100.0
Element prediction: Off
Number of isotope peaks used for i-FIT = 2

Monoisotopic Mass, Even Electron Ions
286 formula(e) evaluated with 2 results within limits (up to 50 closest results for each mass)
Elements Used:
C: 0-50 H: 0-50 N: 0-10 O: 0-5

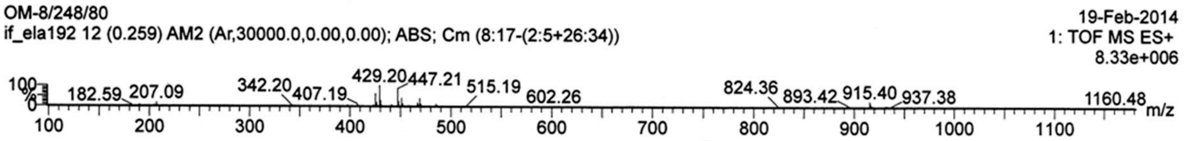

Minimum:				-1.5				
Maximum:		3.0	5.0	100.0				
Mass	Calc. Mass	mDa	PPM	DBE	i-FIT	Norm	Conf(%)	Formula
447.2134	447.2145	-1.1	-2.5	14.5	585.9	0.002	99.80	C24 H27 N6 O3
	447.2113	2.1	4.7	22.5	592.1	6.220	0.20	C35 H27

Figure S43. HRMS spectrum of the olmesartan (**8**).

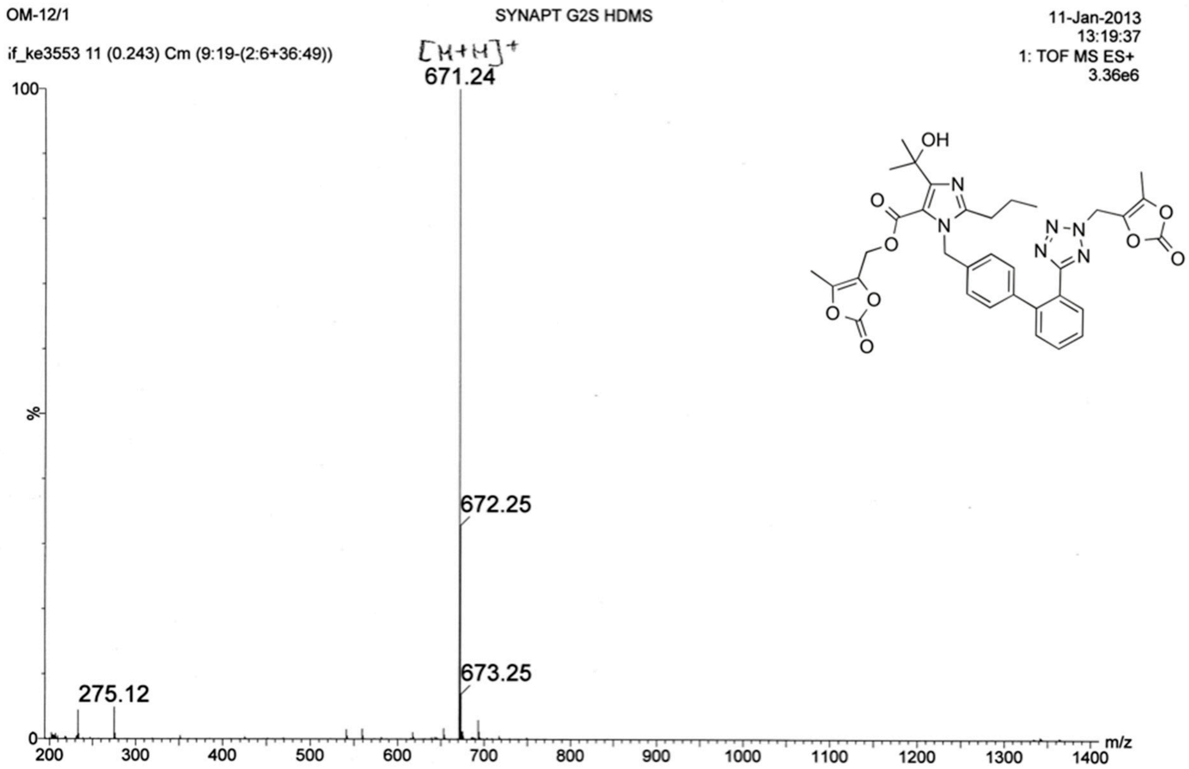

Single Mass Analysis

Tolerance = 10.0 mDa / DBE: min = -1.5, max = 100.0
Element prediction: Off
Number of isotope peaks used for i-FIT = 4

Monoisotopic Mass, Even Electron Ions
922 formula(e) evaluated with 14 results within limits (up to 50 closest results for each mass)
Elements Used:
C: 0-200 H: 0-200 N: 0-10 O: 0-10

Mass	Calc. Mass	mDa	PPM	DBE	i-FIT	Norm	Conf(%)	Formula
Minimum:				-1.5				
Maximum:		10.0	5.0	100.0				
671.2454	671.2447	0.7	1.0	33.5	939.7	6.503	0.15	C46 H31 N4 O2
	671.2466	-1.2	-1.8	20.5	934.7	1.540	21.44	C34 H35 N6 O9
	671.2434	2.0	3.0	28.5	938.9	5.674	0.34	C45 H35 O6
	671.2479	-2.5	-3.7	25.5	935.0	1.808	16.40	C35 H31 N10 O5
	671.2487	-3.3	-4.9	37.5	943.3	10.157	0.00	C51 H31 N2
	671.2420	3.4	5.1	34.5	937.9	4.675	0.93	C42 H27 N10
	671.2407	4.7	7.0	29.5	936.8	3.601	2.73	C41 H31 N6 O4
	671.2506	-5.2	-7.7	24.5	934.3	1.117	32.73	C39 H35 N4 O7
	671.2393	6.1	9.1	24.5	934.7	1.534	21.58	C40 H35 N2 O8
	671.2519	-6.5	-9.7	29.5	937.5	4.322	1.33	C40 H31 N8 O3
	671.2375	7.9	11.8	37.5	944.7	11.547	0.00	C52 H31 O
	671.2538	-8.4	-12.5	16.5	941.9	8.718	0.02	C28 H35 N10 O10
	671.2367	8.7	13.0	25.5	936.9	3.766	2.31	C36 H31 N8 O6
	671.2546	-9.2	-13.7	28.5	941.1	7.891	0.04	C44 H35 N2 O5

Figure S44. HRMS spectrum of the *N*-2 substituted medoxomil impurity **9**.

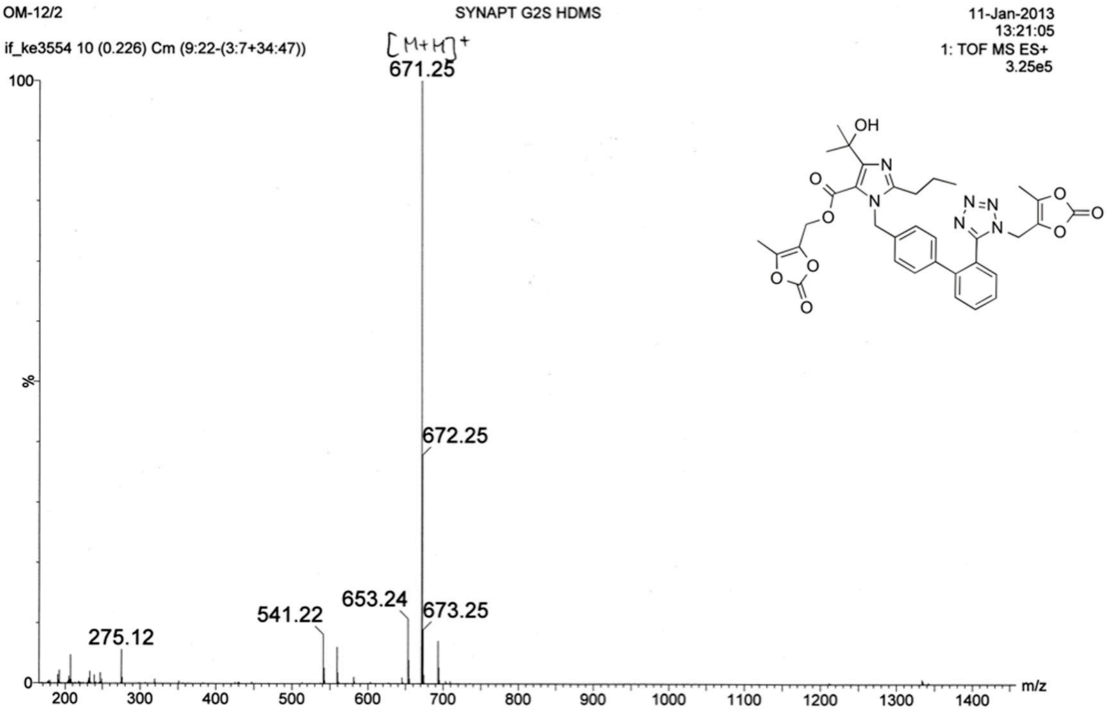

Single Mass Analysis

Tolerance = 10.0 mDa / DBE: min = -1.5, max = 100.0
Element prediction: Off
Number of isotope peaks used for i-FIT = 4

Monoisotopic Mass, Even Electron Ions
922 formula(e) evaluated with 15 results within limits (up to 50 closest results for each mass)
Elements Used:
C: 0-200 H: 0-200 N: 0-10 O: 0-10

Minimum:				-1.5				
Maximum:		10.0	5.0	100.0				
Mass	Calc. Mass	mDa	PPM	DBE	i-FIT	Norm	Conf(%)	Formula
671.2463	671.2466	-0.3	-0.4	20.5	105.4	1.974	13.89	C34 H35 N6 O9
	671.2447	1.6	2.4	33.5	114.8	11.404	0.00	C46 H31 N4 O2
	671.2479	-1.6	-2.4	25.5	103.7	0.364	69.49	C35 H31 N10 O5
	671.2487	-2.4	-3.6	37.5	118.1	14.737	0.00	C51 H31 N2
	671.2434	2.9	4.3	28.5	114.4	11.066	0.00	C45 H35 O6
	671.2420	4.3	6.4	34.5	112.4	9.047	0.01	C42 H27 N10
	671.2506	-4.3	-6.4	24.5	108.6	5.234	0.53	C39 H35 N4 O7
	671.2407	5.6	8.3	29.5	111.8	8.405	0.02	C41 H31 N6 O4
	671.2519	-5.6	-8.3	29.5	111.2	7.772	0.04	C40 H31 N8 O3
	671.2393	7.0	10.4	24.5	111.0	7.587	0.05	C40 H35 N2 O8
	671.2538	-7.5	-11.2	16.5	114.4	10.990	0.00	C28 H35 N10 O10
	671.2546	-8.3	-12.4	28.5	115.9	12.533	0.00	C44 H35 N2 O5
	671.2375	8.8	13.1	37.5	119.8	16.409	0.00	C52 H31 O
	671.2367	9.6	14.3	25.5	105.2	1.836	15.95	C36 H31 N8 O6
	671.2559	-9.6	-14.3	33.5	117.1	13.701	0.00	C45 H31 N6 O

Figure S45. HRMS spectrum of the *N*-1 substituted medoxomil impurity **10.**

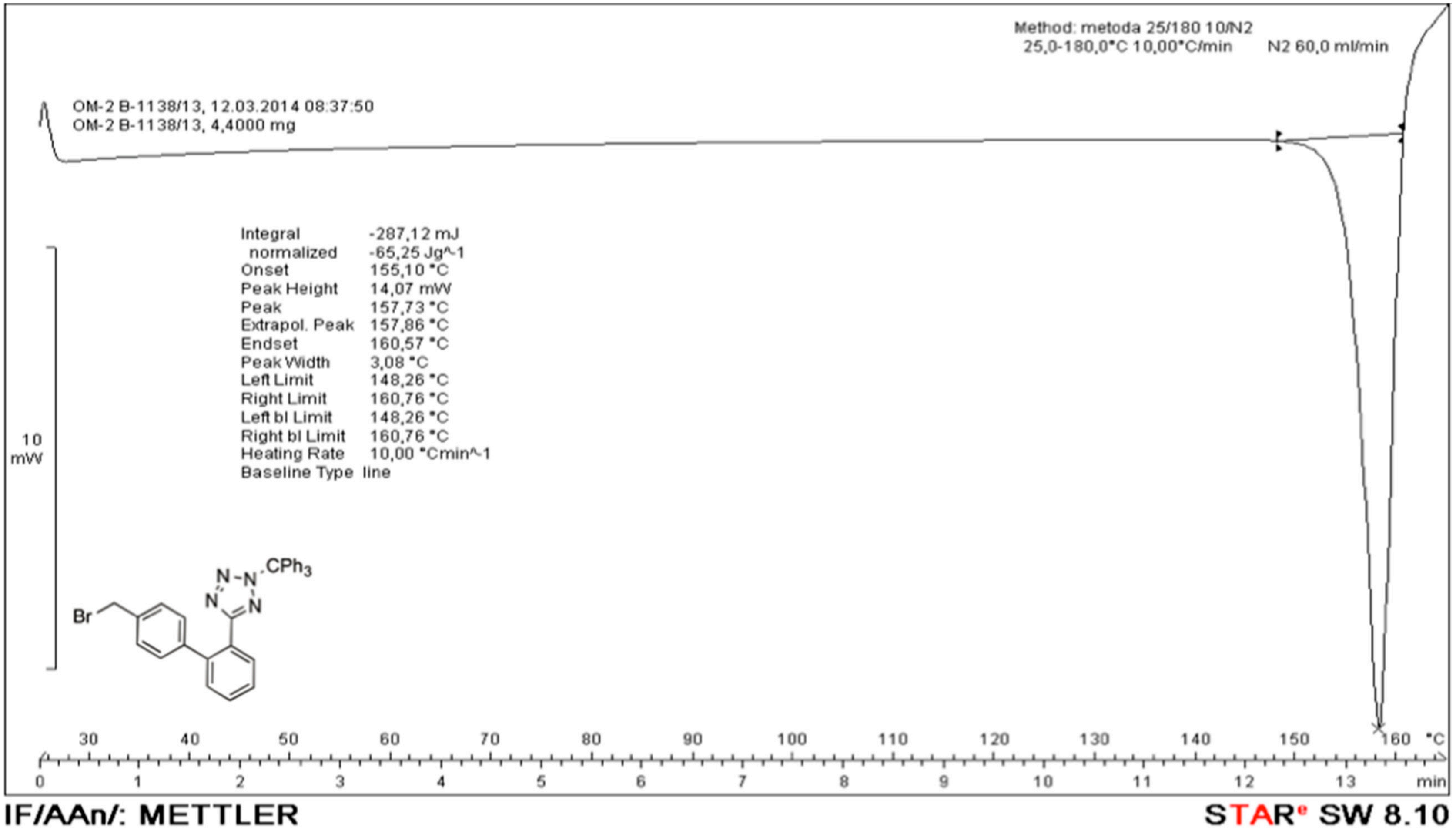

Figure S46. DSC thermogram of the bromide **2**.

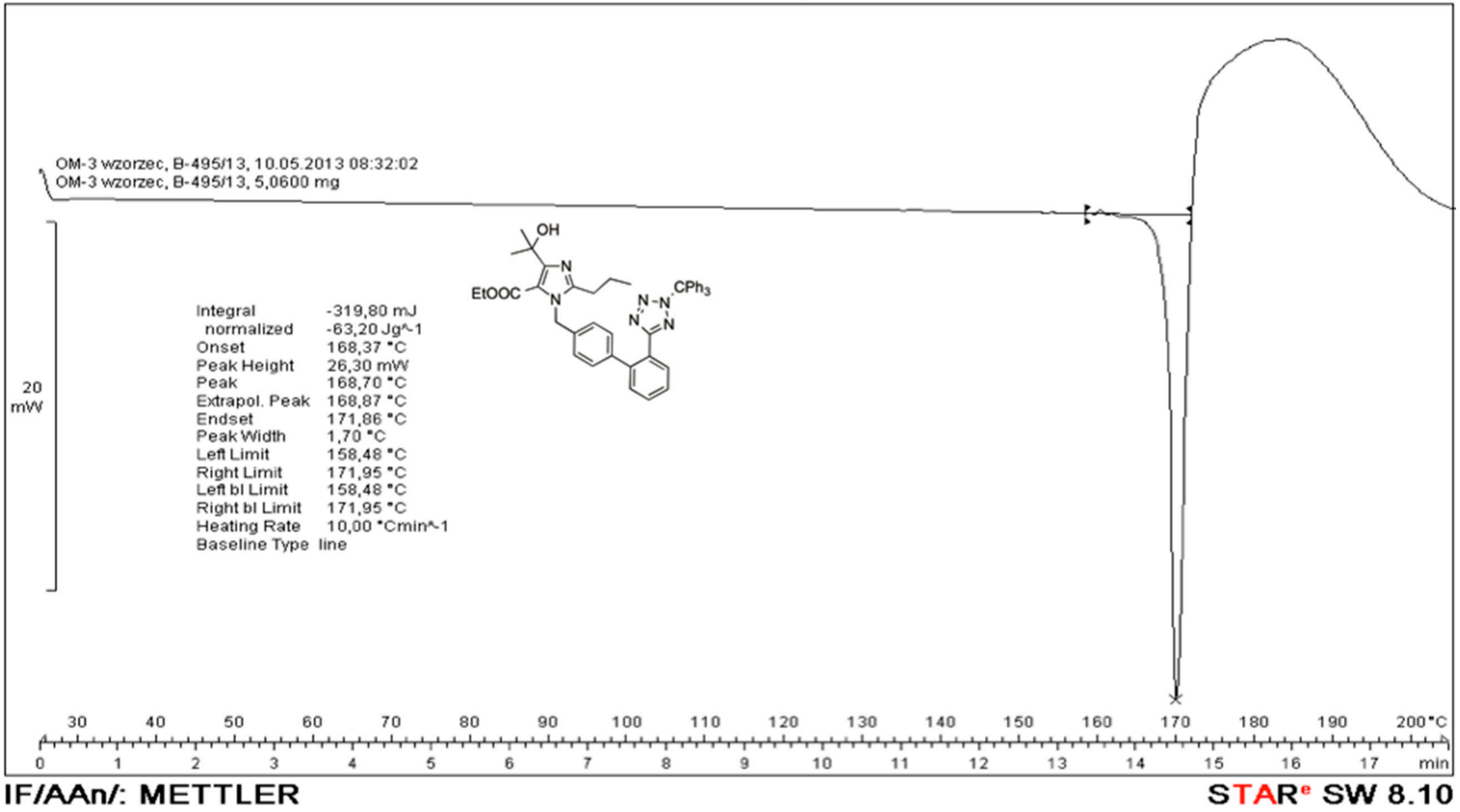

Figure S47. DSC thermogram of the ethyl ester **3**.

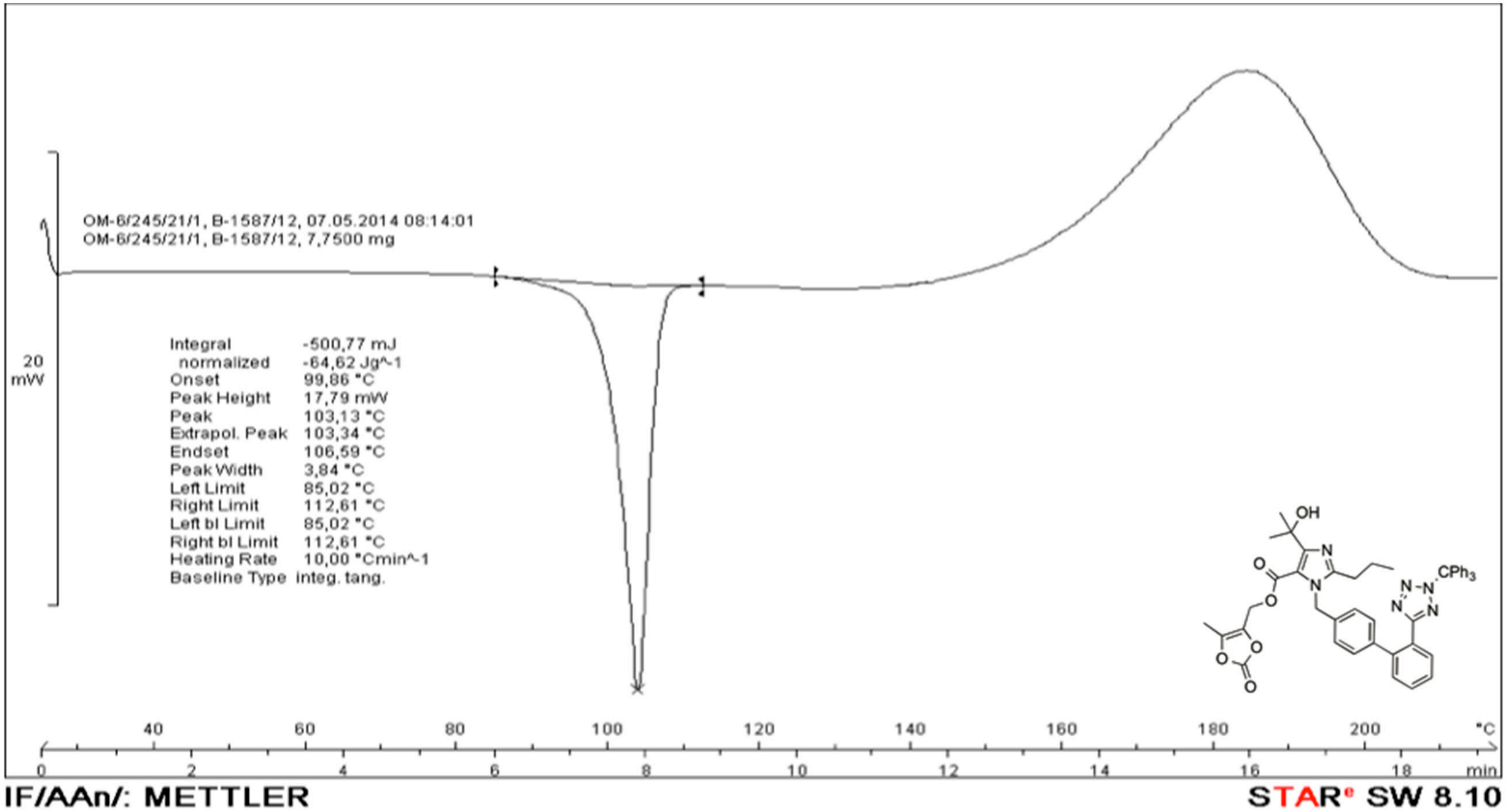

Figure S48. DSC thermogram of the medoxomil ester **6**.

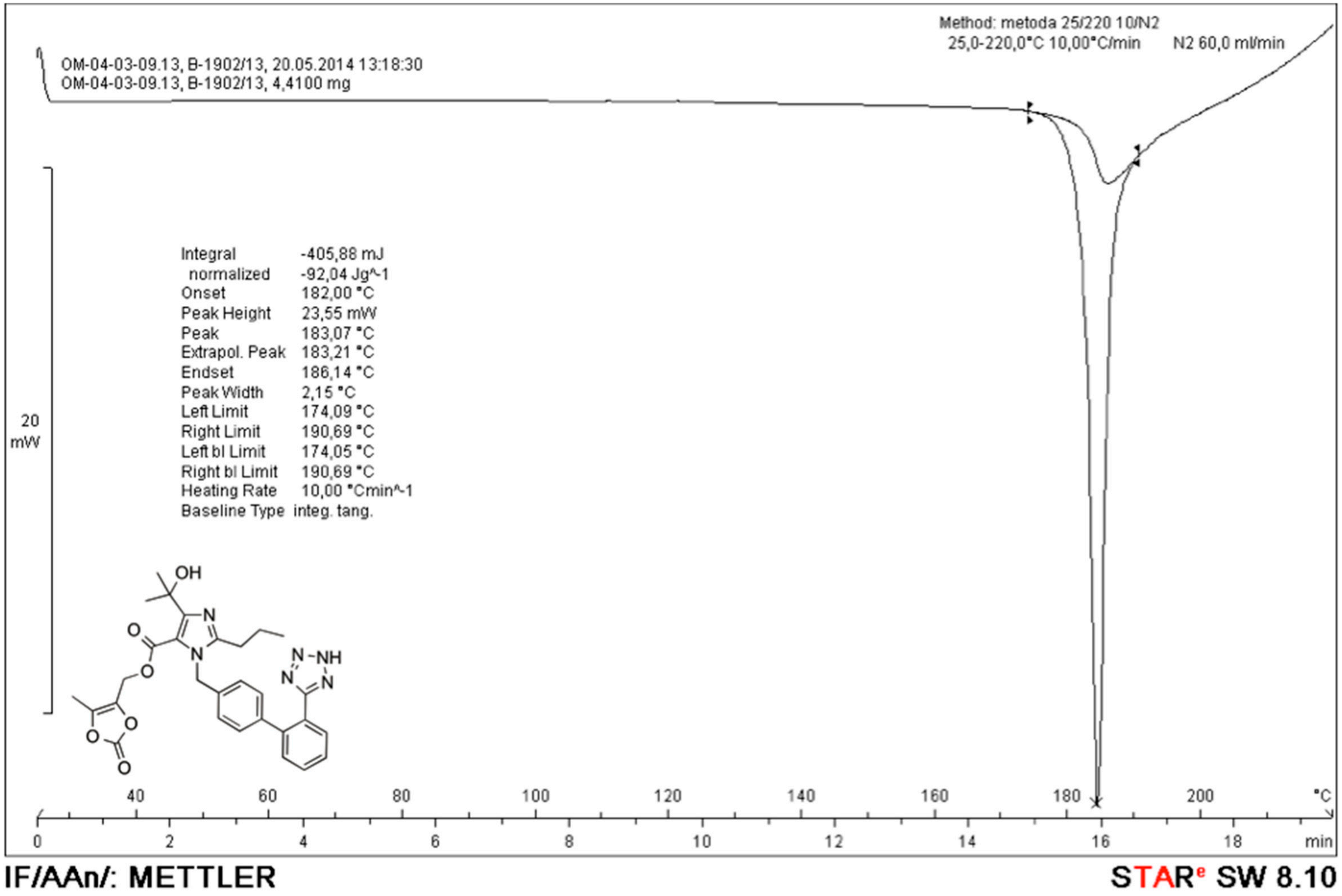

Figure S49. DSC thermogram of the olmesartan medoxomil (**7**).

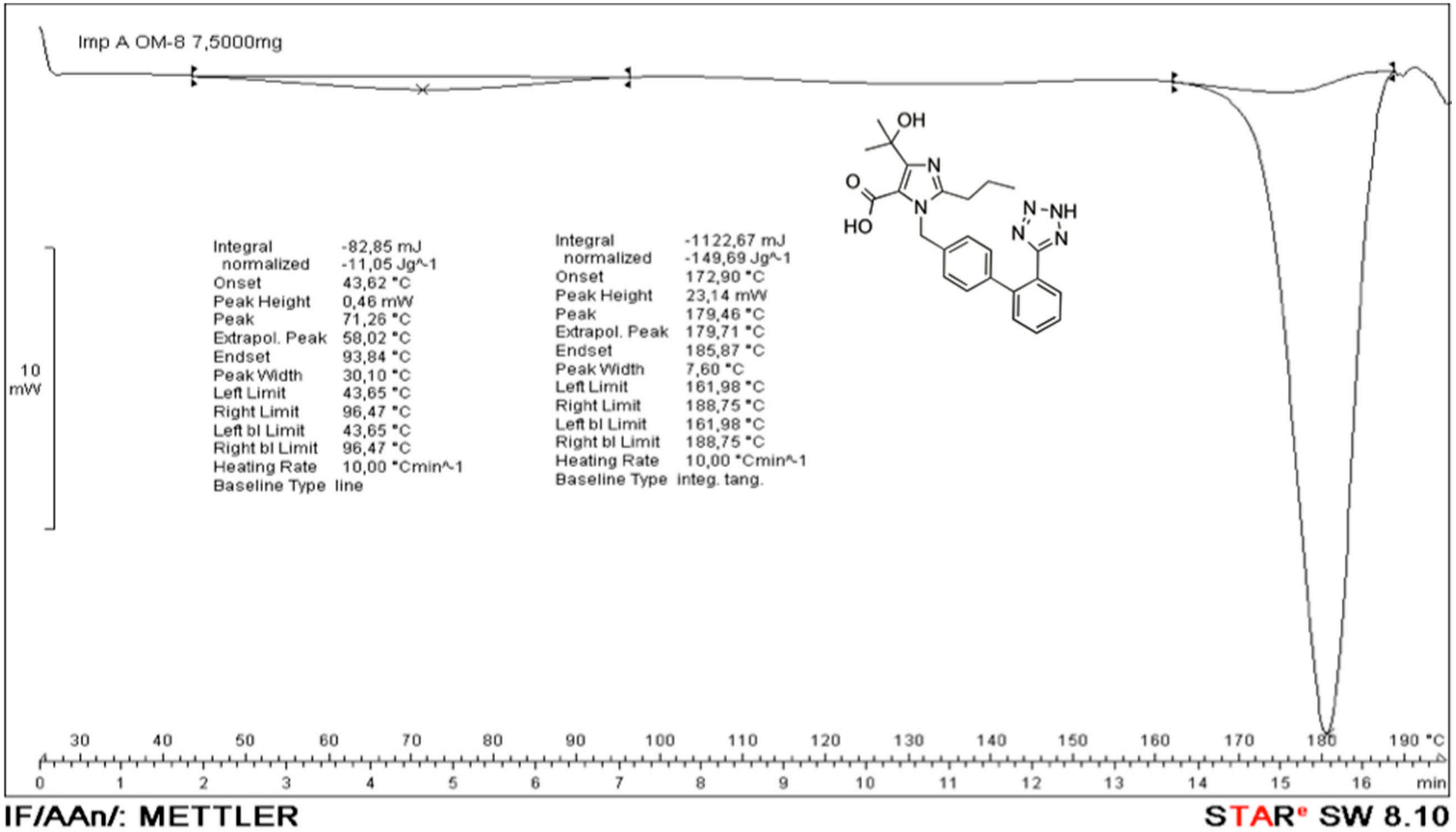

Figure S50. DSC thermogram of the olmesartan (**8**).

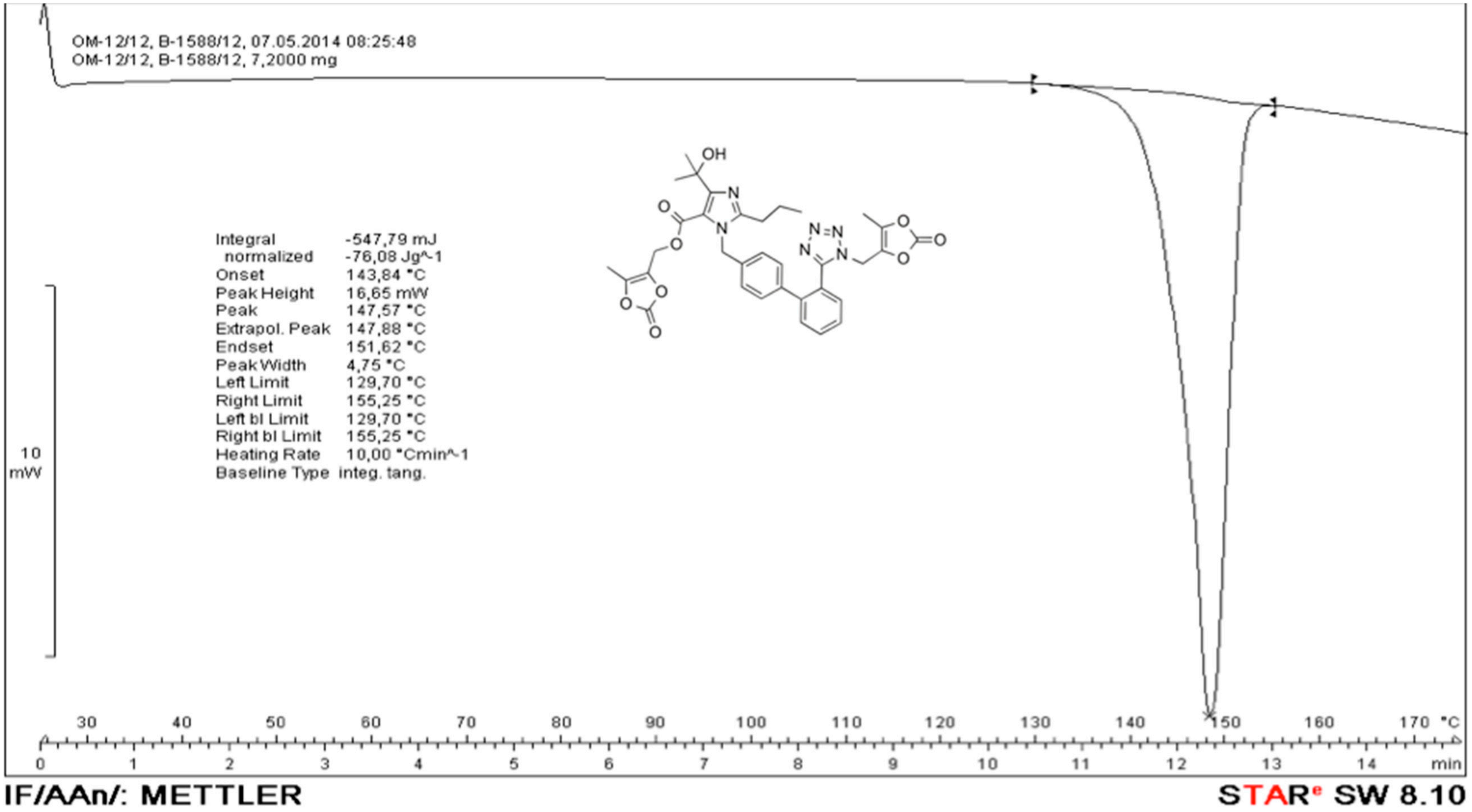

Figure S51. DSC thermogram of the *N*-1 substituted medoxomil impurity **10**.

Abbreviations

AcOEt	ethyl acetate
AcOH	acetic acid
DMF	*N*,*N*-dimethylformamide
DMSO	dimethyl slufoxide
EtOH	ethanol
MeCN	acetonitrile
Me_2CO	acetone
MeOH	methanol
i-PrOH	isopropanol
THF	tetrahydrofuran

References

1. Kamble, R.R.; Biradar, D.B.; Meti, G.Y.; Taj, T.; Gireesh, T.; Khazi, I.A.M.; Vaidyanathan, S.T.; Mohandoss, R.; Sridhar, B.; Parthasarathi, V. An efficient synthesis, X-ray and spectral characterization of biphenyl derivatives. *J. Chem. Sci.* **2011**, *123*, 393–401.
2. Cepanec, I.; Litwic, M.; Koretic, S.; Bartolincic, A.; Druskovic, V.; Sporec, A. Process for Production of (*S*)-*N*-Pentanoyl-*N*-[[2′-(1*H*-Tetrazole-5-yl) [1,1′-Biphenyl]-4-yl]Methyl-L-Valine. WO Patent 2005/049586 A1, 2 June 2005.
3. Manning, R.E.; Reitz, D.B.; Huang, H.C. *N*-Substituted-1,2,4-Triazolone Compounds for Treatment of Cardiovascular Disorders. Patent 2001/020100 A1, 6 September 2001.
4. Nagaraj, B.; Narasegowda, R.S.; Yathirajan, H.S.; Gaonkar, S.L.; Bolte, M. 5-[4′-(Bromomethyl)biphenyl-2-yl]-2-trityl-2*H*-tetrazole. *Acta Cryst.* **2005**, *E61*, o767–o768.
5. Suenaga, S.; Kawagucchi, H.; Kato, T. Process for Producing 4-Bromomethylbiphenyl Compounds. EP Patent 0553879 A2, 4 August 1993.
6. Agelis, G.; Resvani, A.; Durdagi, S.; Spyridaki, K.; Tumová, T.; Slaninová, J.; Giannopoulos, P.; Vlahakos, D.; Liapakis, G.; Mavromoustakos, T.; *et al*. The discovery of new potent non-peptide angiotensin II AT1 receptor blockers: A concise synthesis, molecular docking studies and biological evaluation of *N*-substituted 5-butylimidazole derivatives. *Eur. J. Med. Chem.* **2012**, *55*, 358–374.
7. Roberts, D.A.; Russell, S.T.; Pittam, J.D. Manufacturing Process. Patent EP 0470795 A1, 2 February 1992.
8. Carini, D.J.; Duncia, J.V.; Aldrich, P.E, Chiu, A.T.; Johnson, A.L.; Pierce, M.E.; Price, W.A.; Santella, J.B., III; Wells, G.J.; Wexler, R.R.; *et al*. Nonpeptide angiotensin II receptor antagonists: The discovery of a series of *N*-(biphenylmethyl)imidazoles as potent, orally active antihypertensives. *J. Med. Chem.* **1991**, *34*, 2525–2547.
9. Wang, G.X.; Sun, B.-P.; Ru, Z.-L. Synthesis and characterization of 4′-bromomethyl-2-(*N*-trityl-1*H*-tetrazol-5-yl) biphenyl. *Synth. Commun.* **2008**, *38*, 3577–3581.
10. Aldrich, P.E.; Pierce, M.E.; Duncia, J.J.V. Tetrazole Intermediates to Antihypertensive Compounds. EP Patent 0291969 A2, 23 November 1988.
11. Chao, S.-J.; Hui, X.-P.; Li, S.; Qiu, Z.-Z.; Xu, P.-F.; Zhang, Z.-Y.; Wang, Q.; Guan, Z.-W.; Chin, J. Synthesis and antibacterial activities of novel biphenyltetrazole derivatives bearing 1,3,4-oxadiazole. *J. Chin. Chem. Soc.* **2005**, *52*, 539–544.
12. Sun, D.-G.; Hui, X.-P.; Xu, P.-F.; Zhang, Z.-Y.; Guan, Z.-W. Synthesis of novel biphenyltetrazole derivatives containing 5-methylisoxazole substituted 1,2,4-triazole. *J. Chin. Chem. Soc.* **2007**, *54*, 795–801.
13. Alken, R.-G. Deuterated Biphenyl Derivatives. WO Patent 2003066565 A1, 14 August 2003.
14. Ardecky, R.J.; Carini, D.J.; Duncia, J.J.V.; Wong, P.C. Fused Aryl Substituted Imidazole Angiotensin II Receptor Inhibitors. U.S. Patent 5,254,546, 19 October 1993.
15. Ismail, M.A.H.; Aboul-Enein, M.N.; ElSatar El-Azzouny, A.A.; Abouzid, K.A.M.; Ismail, N.S.M. Design, synthesis, and antihypertensive evaluation of 2′-tetrazolyl and 2′- carboxy-biphenylmethyl-pyrrolidine scaffolds substituted at their N_1, C_3, and C_4 positions as potential angiotensin II AT_1 receptor antagonists. *Med. Chem. Res.* **2015**, *24*, 442–458.
16. Ripka, A.S.; Saunders, J.O.; Kamenecka, T.M.; Griffin, P.R. *N*-Arylmethylindazole Modulators of PPARG. WO Patent 2013078237, 30 May 2013.
17. Choi, S.-K.; Fatheree, P.R.; Hegde, S.; Hudson, R.; Jendza, K.; Marquess, D.; McKinnel, R.M.; Sasikumar, V. Dual-Acting Antihypertensive Agents. U.S. Patent 2008/0188533 A1, 7 August 2008.

18. Yanagisawa, H.; Shimoji, Y.; Fujimoto, K.; Kanazaki, T.; Anemiya, Y.; Koike, H.; Sada, T. 1-Biphenylimidazole Derivavtives, Their Preparation and Their Therapeutic Use. EP Patent 0503785 A1, 16 September 1992.
19. Yanagisawa, H.; Anemiya, Y.; Kanazaki, T.; Shimoji, Y.; Fujimoto, K.; Kitahara, Y.; Sada, T.; Mizuno, M.; Ikeda, M.; Miyamoto, S.; *et al*. Nonpeptide angiotensin II receptor antagonists: Synthesis, biological activities, and structure-activity relationships of imidazole-5-carboxylic acids bearing alkyl, alkenyl, and hydroxyalkyl substituents at the 4-position and their related compounds. *J. Med. Chem.* **1996**, *39*, 323–338.
20. Silvo, Z.; Pecavar, A.; Vrbinc, M.; Jakse, R. Process for the Preparation of Olmesartan Medoxomil. EP Patent 1816131 A1, 8 August 2007.
21. Zupancic, S.; Pecavar, A.; Vrbinc, M.; Osolnik, R. Process for the Preparation of Olmesartan Medoxomil. WO Patent 2007/017135 A1. 15 February 2007.
22. Pati, H.N.; Lahiri, S.; Sabbam, R.K.; Vangala, V.B.; Ramalingam, B.; Hiriyanna, S.G.; Bose P. A convenient and practical synthesis of olmesartan medoxomil methyl ether. *J. Heterocycl. Chem.* **2008**, *45*, 917–920.
23. Rao, B.H.; Rao, I.V.S.; Kanth, V.R.; Rao, K.V.V.P.; Krishna, K.B.; Sundar, B.S. A competent and commercially viable process for the synthesis of the antihypertensive drug olmesartan medoxomil. *Sci. Pharm.* **2015**, *83*, 465–478.
24. Modi, I.A.; Bagepalli, C.S.; Gurusamy, R.; Ponnaiah, R.; Khamar, B.M. An Improved Process for the Preparation of Olmesartan Medoxomil. EP Patent 1916246 A2, 30 April 2008.
25. Babu, K.S.; Reddy, M.S.; Tagore, A.R.; Reddy, G.S.; Sebastian, S.; Varma, M.S.; Venkateswarlu, G.; Bhattacharya, A.; Reddy, P.P.; Anand, R.V. Efficient synthesis of olmesartan medoxomil, an antihypertensive drug. *Synth. Commun.* **2009**, *39*, 291–298.
26. Zupancic, S. Process for Preparing Olmesartan Medoxomil Intermediate. WO Patent 2010026255 A1, 11 March 2010.
27. Raju, M.N.; Kolla, N.; Mukkanti, K.; Bandichhor, R. An efficient and large scale synthesis of olmesartan medoxomil: Antihypertensive drug. *Chem. Biol. Int.* **2013**, *3*, 26–37.
28. Radl, S.; Stach, J. A Method of Removing the Triphenylmethane Protecting Group from Precursors of Antihypertensive Drugs. WO Patent 2007048361 A1, 3 May 2007.
29. Kim, J.W.; Cha, Y.G.; Ryu, H.C.; Kim, S.J. Method of Removing the Triphenylmethane Protection Group. WO Patent 2010067913 A1, 17 June 2010.
30. Babu, K.S.; Tagore, A.R.; Reddy, G.S.; Venkateswarlu, G.; Reddy, P.P.; Anand, R.V. Synthesis of related substances of olmesartan medoxomil, antihypertensive drug. *ARKIVOC* **2010**, *2*, 292–302.
31. Sato, K.; Yagi, T.; Sakuratani, K.; Tani, Y. Method for Producing 1-Biphenylmethylimidazole Compound. EP Patent 2298763 A1, 23 March 2011.
32. Kim, B.C.; Ahn, S.C.; Shin, H.I.; Lee, I.S.; Kim, Y.K. Novel Zinc Azide Complex and a Process for Preparing Tetrazole Derivatives Using the Same. WO Patent 2012148148 A2, 1 November 2012.
33. Venkanna, G.; Madhusudhan, G.; Mukkanti, K.; Sankar, A.; Sampath Kumar, Y.; Venakata Narayana, G. Synthesis and characterization of process-related impurities of antihypertensive drug olmesartan medoxomil. *J. Chem.* **2013**, *2013*, 516459.

© 2015 by the authors; licensee MDPI, Basel, Switzerland. This article is an open access article distributed under the terms and conditions of the Creative Commons by Attribution (CC-BY) license (http://creativecommons.org/licenses/by/4.0/).
